# The genome of *Pelobacter carbinolicus* reveals surprising metabolic capabilities and physiological features

**DOI:** 10.1186/1471-2164-13-690

**Published:** 2012-12-10

**Authors:** Muktak Aklujkar, Shelley A Haveman, Raymond DiDonato, Olga Chertkov, Cliff S Han, Miriam L Land, Peter Brown, Derek R Lovley

**Affiliations:** 1 University of Massachusetts Amherst, Amherst, MA, 01003, USA; 2 Department of Energy, Joint Genome Institute, Walnut Creek, CA, 94598, USA; 3 Oak Ridge National Laboratory, Oak Ridge, TN, 37830, USA

**Keywords:** *Pelobacter*, Genome, Metabolism, Physiology, *Geobacter*, 2,3-butanediol

## Abstract

**Background:**

The bacterium *Pelobacter carbinolicus* is able to grow by fermentation, syntrophic hydrogen/formate transfer, or electron transfer to sulfur from short-chain alcohols, hydrogen or formate; it does not oxidize acetate and is not known to ferment any sugars or grow autotrophically. The genome of *P*. *carbinolicus* was sequenced in order to understand its metabolic capabilities and physiological features in comparison with its relatives, acetate-oxidizing *Geobacter* species.

**Results:**

Pathways were predicted for catabolism of known substrates: 2,3-butanediol, acetoin, glycerol, 1,2-ethanediol, ethanolamine, choline and ethanol. Multiple isozymes of 2,3-butanediol dehydrogenase, ATP synthase and [FeFe]-hydrogenase were differentiated and assigned roles according to their structural properties and genomic contexts. The absence of asparagine synthetase and the presence of a mutant tRNA for asparagine encoded among RNA-active enzymes suggest that *P*. *carbinolicus* may make asparaginyl-tRNA in a novel way. Catabolic glutamate dehydrogenases were discovered, implying that the tricarboxylic acid (TCA) cycle can function catabolically. A phosphotransferase system for uptake of sugars was discovered, along with enzymes that function in 2,3-butanediol production. Pyruvate:ferredoxin/flavodoxin oxidoreductase was identified as a potential bottleneck in both the supply of oxaloacetate for oxidation of acetate by the TCA cycle and the connection of glycolysis to production of ethanol. The *P*. *carbinolicus* genome was found to encode autotransporters and various appendages, including three proteins with similarity to the geopilin of electroconductive nanowires.

**Conclusions:**

Several surprising metabolic capabilities and physiological features were predicted from the genome of *P*. *carbinolicus*, suggesting that it is more versatile than anticipated.

## Background

*Pelobacter carbinolicus* is a bacterial species isolated from anoxic mud by anaerobic enrichment on the growth substrate 2,3-butanediol, an end product of fermentations [1]. *P*. *carbinolicus* was assigned to the genus *Pelobacter* of the *Deltaproteobacteria* on the basis of its ability to consume fermentatively alcohols such as 2,3-butanediol, acetoin and ethanol, but not sugars, with acetate plus ethanol and/or hydrogen as the end products [2]. Subsequently, *Pelobacter* species, which cannot oxidize acetate, were shown to be phylogenetically distributed throughout the order *Desulfuromonadales*[3,4], among species that grow by oxidation of acetate with either S° or Fe(III) but not sulfate as the electron acceptor. *P*. *carbinolicus* belongs to the family *Desulfuromonadaceae*[4-7] and *Pelobacter propionicus* to *Geobacteraceae*. The complete genome sequence of *P*. *carbinolicus* has led to the discoveries that it expresses *c*-type cytochromes [8] and that it utilizes Fe(III) as a terminal electron acceptor indirectly via reduction of S° [9]. *In silico* metabolic models have been constructed for *P*. *carbinolicus* and *P*. *propionicus*[10], their genomes have been compared to those of acetate-oxidizing, non-fermentative *Geobacteraceae*[11], and a shortage of histidyl-tRNA caused by the CRISPR locus has been proposed to account for the loss of some ancestral genes such as multiheme *c*-type cytochromes by the *P*. *carbinolicus* genome [12]. However, there are many features of the *P*. *carbinolicus* genome that these studies have not addressed. The aim of this paper is to present these features as they pertain to current assumptions and questions about the physiology and metabolism of *P*. *carbinolicus*, from substrate uptake to enzymology to electron transfer processes and outer surface features.

## Results and discussion

### Contents of the *P*. *carbinolicus* genome

The genome of *P*. *carbinolicus* was sequenced and the annotation was curated as detailed in the Methods section. The previous annotation consists of 3352 orfs, 33 pseudogenes, and 63 structural RNA genes. During curation, 89 false orfs and one pseudogene were removed, five pseudogenes were reclassified as orfs and one orf as a pseudogene, 46 orfs and 31 pseudogenes were added, one tRNA gene was reclassified as a mutant tRNA gene, and 448 nucleotide sequence features including riboswitches, CRISPR spacers and multicopy sequences were identified. The current annotation consists of 3313 orfs, 58 pseudogenes, 62 structural RNAs and 449 other nucleotide sequence features. The locations of multicopy nucleotide sequences of the *P*. *carbinolicus* genome relative to genes, their coordinates and their alignments can be found in the supplementary material (Additional file 1: Table S1; Additional file 2: Figure S1).

### The mutant tRNA

**Figure 1:**
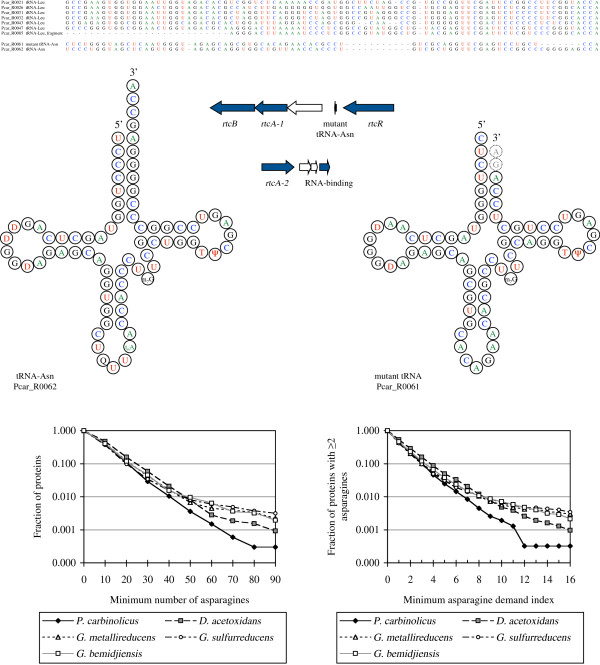
**The mutant tRNA**-**Asn of *P. carbinolicus *and patterns of asparagine usage in proteins.** The alignment (top) shows that Pcar_R0061 lacks features typical of the six tRNA-Leu species; it is a mutated copy of tRNA-Asn. The cloverleaf diagrams of tRNA-Asn (left) and the Pcar_R0061 transcript (right) illustrate that base-pairing is retained through reciprocal mutations and unlike the extended CCA-3^′^ end of tRNA-Asn, the 3^′^ end of a mature Pcar_R0061 transcript is predicted to be recessed and possibly longer by two bases. The Pcar_R0061 transcript may be modified similarly to tRNA-Asn, except for the queuosine and threonylcarbamyl modifications of the anticodon loop. The operon diagrams (middle) show that Pcar_R0061 is in one of two gene clusters encoding RNA 3^′^-phosphate cyclases. The graphs (bottom) show that the *P*. *carbinolicus* genome encodes fewer proteins with either more than 50 asparagine residues (left) or an asparagine demand index above 7.0 (right), compared to other *Desulfuromonadales*.

The tRNA gene that had to be reclassified, Pcar_R0061, was originally annotated as specific for leucine, but its sequence does not align with the six true tRNA-Leu genes of *P*. *carbinolicus* (Figure 1); it aligns with tRNA-Asn (Pcar_R0062) except that the asparagine anticodon GUU has mutated to a leucine anticodon CAG and a deletion of seven bases has buried the aminoacylation site (CCA-3^′^) within the acceptor stem. The deletion is expected to interfere with 3^′^ end trimming and aminoacylation of the mutant tRNA and prevent the mistranslation of CUG leucine codons as asparagine. Another indication that Pcar_R0061 may not function in protein translation is that the universally conserved frameshift control base U33 has mutated to A. Mutations at other positions in Pcar_R0061 are reciprocal (preserving base-pairing in the folded transcript), indicating that the mutant tRNA may be under selective pressure to maintain the cloverleaf fold (Figure 1) for some function; it is not a pseudogene.

The location of Pcar_R0061 suggests a function in RNA repair or editing (Figure 1). On its 3^′^ side, three genes transcribed in the same direction encode a stomatin-like multimeric membrane protein (Pcar_2837), an RNA 3^′^-phosphate cyclase (Pcar_2836 or *rtcA1*) [13] and an RNA 2^′^,3^′^-cyclic phosphate--5^′^-hydroxyl ligase (Pcar_2835 or *rtcB*) [14]. On the 5^′^ side of Pcar_R0061, transcribed divergently, is the transcriptional regulator of *rtcAB* (*rtcR* Pcar_2838) [15]. Accordingly, the mutant tRNA may be either a substrate or a guide for the RNA-active enzymes. Another RNA 3^′^-phosphate cyclase (Pcar_2495 or *rtcA2*) and RNA-binding protein (Pcar_2498) may also participate.

### Asparagine metabolism

The mutant tRNA might also have a role in the synthesis of asparagine, for which no asparagine synthetase was identified in *P*. *carbinolicus*[10]. *P*. *carbinolicus* is predicted to convert oxaloacetate to aspartate using both a nonspecific aminotransferase found in *Geobacteraceae* (Pcar_2772) and an aspartate-specific aminotransferase (Pcar_1573) with 30% sequence identity to that of *Thermus thermophilus*[16]. In *T*. *thermophilus*, which lacks asparagine synthetase and asparaginyl-tRNA synthetase, aspartate is attached to tRNA-Asn by a non-discriminating aspartyl-tRNA synthetase [17], then corrected to asparaginyl-tRNA by the amidotransferase system (homologous to Pcar_2167-Pcar_2169). In contrast, *P*. *carbinolicus* possesses an asparaginyl-tRNA synthetase (Pcar_0586) and a discriminating aspartyl-tRNA synthetase (Pcar_1040) similar to those of *Geobacteraceae*, but no non-discriminating aspartyl-tRNA synthetase. Therefore, either *P*. *carbinolicus* possesses an unidentified novel asparagine synthetase or its asparaginyl-tRNA synthetase can be modulated to accommodate aspartate in lieu of asparagine, with subsequent correction by the amidotransferase system. In the latter case, the role of the tRNA-Asn-derived mutant tRNA might be to modulate the asparaginyl-tRNA synthetase homodimer by binding to one subunit in a manner that allows the other subunit to react tRNA-Asn with aspartate.

If asparaginyl-tRNA synthesis is difficult in *P*. *carbinolicus*, one would expect the *P*. *carbinolicus* genome to encode fewer proteins with numerous closely spaced asparagine residues than the genomes of other *Desulfuromonadales*. A similar expectation for a histidyl-tRNA synthesis defect was previously validated [12]. When the total number of asparagine residues and the asparagine demand index (defined as the number of asparagines divided by the harmonic mean distance between them) were computed for every protein in *P*. *carbinolicus* and other *Desulfuromonadales*, the resulting patterns showed that proteins with numerous and closely spaced asparagine residues are in fact fewer in *P*. *carbinolicus* (Figure 1), as if asparaginyl-tRNA is limiting.

### Three 2,3-butanediol dehydrogenases

The following seven sections will focus on different growth substrates. The initial description of *P*. *carbinolicus* established that it consumes all three stereoisomers of 2,3-butanediol [1], whereas many other species are limited by the stereospecificities of their 2,3-butanediol dehydrogenases [18-20]. MDR family dehydrogenases that act on (*R*)-chiral hydroxyl groups interconvert (*2R3R*)-2,3-butanediol with (*R*)-acetoin and/or *meso*-2,3-butanediol with (*S*)-acetoin, while SDR family dehydrogenases that act on (*S*)-chiral hydroxyl groups interconvert (*2S3S*)-2,3-butanediol with (*S*)-acetoin and/or *meso*-2,3-butanediol with (*R*)-acetoin. Genome sequencing of *P*. *carbinolicus* revealed three 2,3-butanediol dehydrogenases (MDR *budX* Pcar_0330, SDR *budY* Pcar_0903, SDR *budZ* Pcar_2068), but the published studies have either noted only one [11] or assigned them to only two stereoisomers [10]. The correct assignment of all three enzymes to their substrates could have commercial value for the production of optically pure 2,3-butanediol [21,22]. The BudX protein has 39% sequence identity to enzymes of *Paenibacillus polymyxa*[19,20,23-25] and *Bacillus subtilis*[26,27] that have higher activity with (*2R3R*)-2,3-butanediol than with *meso*-2,3-butanediol. BudY and BudZ are most closely related to each other, and 40-47% identical to *meso*-2,3-butanediol dehydrogenase of *Klebsiella pneumoniae*[28] and (*2S3S*)-2,3-butanediol dehydrogenase of *Corynebacterium glutamicum*[29]. The active site of the *C*. *glutamicum* enzyme excludes *meso*-2,3-butanediol and is formed by eleven amino acid residues [30], all of which are conserved in BudY. Two of these residues are different in the *K*. *pneumoniae* enzyme that excludes (*2S3S*)-2,3-butanediol [31], and two residues are different in BudZ (Table 1). Therefore, BudY may be tentatively annotated as (*2S3S*)-2,3-butanediol dehydrogenase and BudZ as *meso*-2,3-butanediol dehydrogenase, assignments that will have to be validated experimentally. *P*. *carbinolicus* does not grow on (*2S3S*)-2,3-butanediol alone as it does with the other stereoisomers (S. Haveman, unpublished), suggesting that BudY may not be expressed constitutively and BudZ may have a strong preference for *meso*-2,3-butanediol.

**Table 1:** **Conservation of active site residues of *C***. ***glutamicum*** (***2S***,***3S***)-**2**,**3**-**butanediol dehydrogenase in Pcar**_**0903**, **Pcar**_**2068 and Ppro**_**3110**, **compared to *meso***-**2**,**3**-**butanediol dehydrogenase of *K***. ***pneumoniae***

***C***. ***glutamicum***	***K***. ***pneumoniae***	**Pcar**_**0903 BudY**	**Pcar**_**2068 BudZ**	**Ppro**_**3110**
S141	S139	S151	*C144*	S148
I142	*Q140*	I152	I145	I149
A143	A141	A153	A146	A150
F148	*N146*	F158	*L151*	F155
Y154	Y152	Y164	Y157	Y161
P184	P182	P194	P187	P191
G185	G183	G195	G188	G192
I186	I184	I196	I189	I193
M191	M189	M201	M194	M198
W192	W190	W202	W195	W199
I195	I193	I205	I198	I202

Mutations that could alter the substrate specificity from (*2S*,*3S*)-2,3-butanediol to *meso*-2,3-butanediol are indicated in italics.

Expression of *budX* and *budZ* is upregulated during growth of *P*. *carbinolicus* on racemic acetoin compared to growth on ethanol [9]. This may mean that the two enzymes act in concert to interconvert the stereoisomers of acetoin through *meso*-2,3-butanediol. The stereospecificity of acetoin dehydrogenase has not been determined experimentally, but *Neisseria winogradskyi* and *Micrococcus ureae* oxidize *meso*-2,3-butanediol through (*S*)-acetoin only [18,20]. If acetoin dehydrogenase prefers (*S*)-acetoin, *P*. *carbinolicus* could use first BudZ to reduce the carbonyl group of (*R*)-acetoin to an (*S*)-chiral hydroxyl group in *meso*-2,3-butanediol, then BudX to oxidize the (*R*)-chiral hydroxyl group to a carbonyl group in (*S*)-acetoin. Strains of *P*. *carbinolicus* growing on acetoin transiently accumulate *meso*-2,3-butanediol to a lesser extent than optically active 2,3-butanediol [32], consistent with conversion of (*R*)-acetoin via *meso*-2,3-butanediol to (*S*)-acetoin for degradation while (*2R3R*)-2,3-butanediol serves as an electron sink. Expression of *budY* does not change during growth on racemic acetoin [9], consistent with the prediction that BudY has no activity on *meso*-2,3-butanediol. The Pcar_2067 gene on the 3^′^ side of *budZ*, encoding an SDR family oxidoreductase, is also upregulated on acetoin and should be investigated for a possible role in acetoin/2,3-butanediol metabolism.

### The acetoin dehydrogenase gene cluster

**Figure 2:**
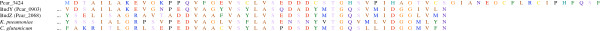
**Alignment of the protein sequence of Pcar_3424,a hypothetical protein encoded near *budX, *with the C-termini of BudY and BudZ of *P. carbinolicus *and their characterized homologs: *meso-*2,3-butanediol dehydrogenase of *K*. *pneumoniae *and(*2S*,*3S*)-2,3-butanediol dehydrogenase of *C. glutamicum***.

Genome sequencing revealed that the previously sequenced acetoin dehydrogenase genes *acoABCSL* of *P*. *carbinolicus*[33] are within a cluster of 28 genes (Additional file 3: Table S2) mostly upregulated during growth on acetoin and all transcribed in the same direction [9]. The third gene of this cluster is *budX* (Pcar_0330) and the seventh gene (Pcar_3424) encodes a small protein similar to the C-termini of BudY and BudZ (Figure 2), which might function as a modulator of 2,3-butanediol metabolism. The Pcar_0329 gene on the 5^′^ side of *budX* encodes a multitransmembrane protein that might facilitate transport of acetoin and 2,3-butanediol across the inner membrane, and Pcar_0334, the eighth gene of the cluster, encodes a possible modulator of transport, a protein of the DUF190 family distantly related to the GlnK protein that controls the ammonium transport channel. Five genes of the cluster (Pcar_0337, Pcar_0338, Pcar_0339, Pcar_0340, Pcar_0342) encode a partial set of enzymes for biosynthesis of thiamin, a cofactor of acetoin dehydrogenase; amidst them is the *acoX* gene of unknown function (Pcar_0341) that is typical of acetoin dehydrogenase gene clusters. The *P*. *carbinolicus* genome possesses seemingly redundant genes for each thiamin biosynthesis enzyme (Additional file 4: Table S3), and most are quite divergent in sequence from their homologs in *Geobacteraceae*, while *thiH* has not been identified in any *Geobacteraceae* genome. *P*. *carbinolicus* has only one source of lipoate, the other cofactor of acetoin dehydrogenase: the *lipB* (Pcar_0350) gene product transfers an octanoyl group from an acyl carrier protein to the enzyme and then the *acoS* (Pcar_0346) gene product converts it to a dihydrolipoyl group. *P*. *carbinolicus* lacks the *lplA* gene of *Geobacteraceae* to attach free octanoate or dihydrolipoate to enzymes, and although their *lipB* gene product sequences align well, *acoS* is very different in sequence from its counterpart in *Geobacteraceae*, *lipA*. Altogether, the ancillary enzymes of acetoin dehydrogenase appear to be a mosaic of genes of various origins.

AcoR, the activator of acetoin dehydrogenase gene expression [34,35], has three counterparts in *P*. *carbinolicus* encoded by *acoR1* (Pcar_0336) in the acetoin dehydrogenase gene cluster, *acoR2* (Pcar_0902) next to *budY*, and *acoR3* (Pcar_1734) next to a gene encoding an oxidoreductase of the aldo/keto reductase family (Pcar_1733) that should be investigated for a possible function in acetoin/2,3-butanediol metabolism. The three AcoR proteins share 52-73% sequence identity. Their multiplicity suggests that control of acetoin/2,3-butanediol metabolism in *P*. *carbinolicus* may be more complex than in other species. Indeed, our unpublished microarray data for *P*. *carbinolicus* growing by disproportionation of 2,3-butanediol to ethanol plus acetate indicate 4.5-fold and 9.7-fold upregulation of *acoR2* and *acoR3*, respectively, compared to growth by oxidation of 2,3-butanediol to acetate, and 5.6-fold and 9.2-fold upregulation, respectively, compared to growth by oxidation of ethanol to acetate. None of the three *acoR* genes changes expression during growth on acetoin.

**Figure 3:**
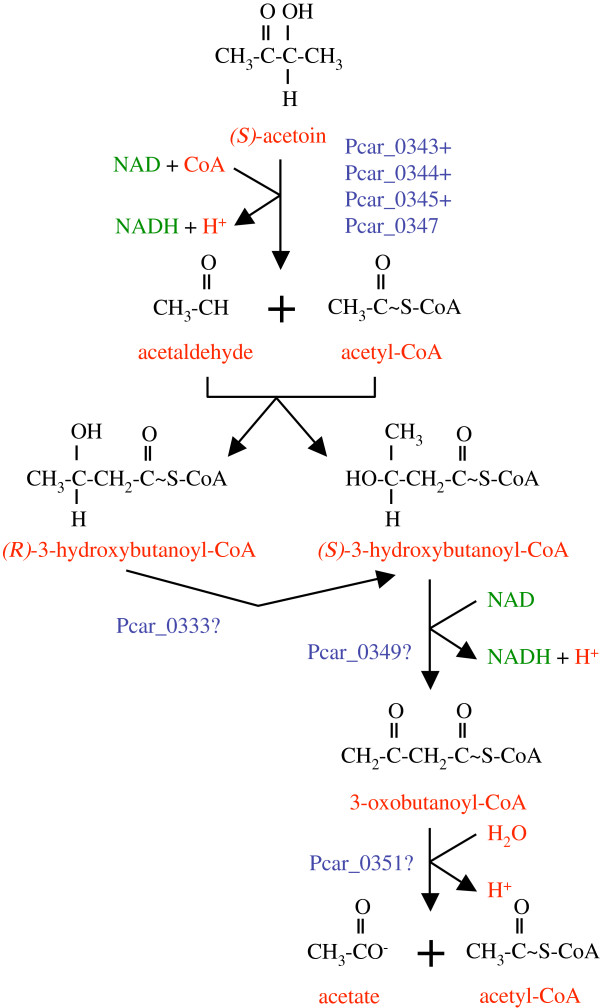
**Hypothesized roles of acyl-CoA-active genes of the acetoin dehydrogenase gene cluster in *P*. *carbinolicus*.** The products of acetoin dehydrogenase, acetaldehyde and acetyl-CoA, might accidentally undergo aldol addition before leaving the active site, forming either isomer of 3-hydroxybutanoyl-CoA. The methylmalonyl-CoA epimerase family protein (Pcar_0333) might convert the (*R*)-isomer to the (*S*)-isomer, which 3-hydroxyacyl-CoA dehydrogenase (Pcar_0349) can oxidize. A hydrolase/acyltransferase encoded in the cluster (Pcar_0351) might split 3-oxobutanoyl-CoA into acetate plus acetyl-CoA.

Other gene products of the cluster (Pcar_0333, Pcar_0349, Pcar_0351) are predicted to act on acyl-CoA substrates (Additional file 3: Table S2), which is surprising because there is not a single acyl-CoA dehydrogenase, enoyl-CoA hydratase, or thiolase gene in *P*. *carbinolicus*. These enzymes might degrade a byproduct of acetoin dehydrogenase formed by accidental aldol addition of acetyl-CoA to acetaldehyde (Figure 3). Consistent with this prediction, acetate is a minor product when *P*. *carbinolicus* oxidizes 1,3-butanediol to 3-hydroxybutanoate [32].

### Metabolism of glycerol and 1,3-propanediol

**Figure 4:**
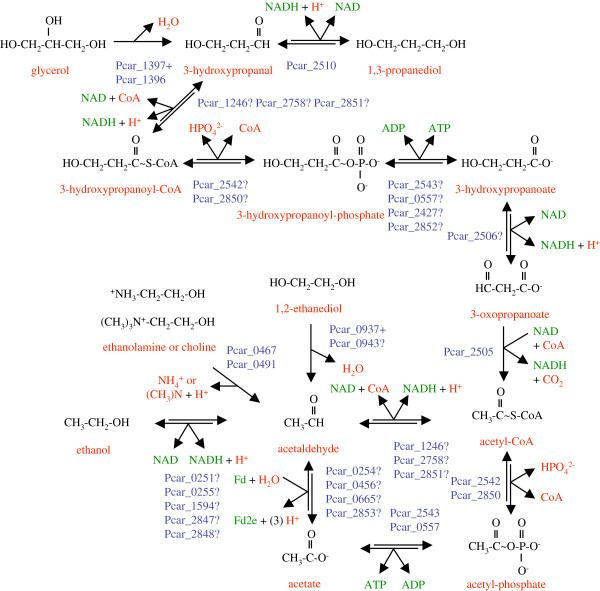
**Metabolic pathways for oxidation of glycerol,1,3-propanediol,1,2-ethanediol,ethanolamine,choline and ethanol by *P*. *carbinolicus*.** Each of these substrates is converted to acetaldehyde, which is either oxidized to acetyl-CoA to yield energy by substrate-level phosphorylation or reduced to ethanol to dispose of NADH or oxidized to acetate to supply doubly reduced ferredoxin (Fd2e).

*P*. *carbinolicus* was initially described as unable to degrade glycerol [1], but some strains in pure culture disproportionate glycerol to 1,3-propanediol plus 3-hydroxypropanoate with acetate as a carbon source [36] and the type strain utilizes glycerol with *Geobacter sulfurreducens* as a syntrophic partner (Z. Summers, personal communication). Therefore, an attempt was made to delineate the pathway of glycerol metabolism in *P*. *carbinolicus* based on its genome (Figure 4). The glycerol dehydratase (Pcar_1397) and activating enzyme (Pcar_1396) of *P*. *carbinolicus* are 57% and 38% identical to characterized homologs in *Clostridium butyricum*[37], respectively. The *C*. *butyricum* enzyme dehydrates both glycerol to 3-hydroxypropanal and 1,2-propanediol to propanal, consistent with utilization of 1,2-propanediol by a *P*. *carbinolicus* strain [32]. Oxidation of 3-hydroxypropanal to 3-hydroxypropanoate may yield one ATP if 3-hydroxypropanoyl-CoA is an intermediate. *P*. *carbinolicus* possesses multiple predicted isozymes of acetaldehyde dehydrogenase (Pcar_1246, Pcar_2758, Pcar_2851), phosphate acetyltransferase (Pcar_2542 and Pcar_2850) and acetate kinase (Pcar_2543 and Pcar_0557) that could nonspecifically catalyze these reactions. The final ATP-yielding step might also be catalyzed by propanoate kinase (Pcar_2427) or butanoate kinase (Pcar_2852). To oxidize 3-hydroxypropanoate to 3-oxopropanoate, a candidate alcohol dehydrogenase (Pcar_2506) is encoded next to the gene for the next enzyme, a decarboxylating 3-oxopropanoate/2-methyl-3-oxopropanoate dehydrogenase (Pcar_2505) with 41% sequence identity to that of *B*. *subtilis*[38]. This step produces acetyl-CoA, which yields one ATP upon conversion to acetate. Of the predicted energy yield of two ATP per glycerol molecule in syntrophic culture, a part must be expended to convert three NADH to hydrogen/formate molecules, which *G*. *sulfurreducens* consumes along with acetate.

*P*. *carbinolicus* possesses a 1,3-propanediol dehydrogenase (Pcar_2510) that is 66% identical to the characterized *K*. *pneumoniae* enzyme [39]. Thus, the machinery may be present for *P*. *carbinolicus* in pure culture to derive two ATP from fermentation of four glycerol molecules to three molecules of 1,3-propanediol and one of acetate (Figure 4). *P*. *carbinolicus* in syntrophic culture may also oxidize 1,3-propanediol to yield two ATP, part of which it must expend to convert four NADH to four hydrogen/formate molecules to transfer to a syntrophic partner [32]. Nearby the 3-oxopropanoate dehydrogenase gene are genes for a hydrogenase (*hndD1* Pcar_2502) and an NADPH oxidoreductase subunit (Pcar_2503) similar to SfrB of *Geobacteraceae*[40] that together may dispose of electrons from glycerol and 1,3-propanediol.

The glycerol dehydratase gene cluster and the 1,3-propanediol dehydrogenase gene cluster share several notable features (Additional file 3: Table S2). The Pcar_1398 gene on the 5^′^ side of the glycerol dehydratase genes and the Pcar_2509 gene on the 3^′^ side of the 1,3-propanediol dehydrogenase gene encode multitransmembrane proteins that share 47-54% sequence identity with the predicted acetoin/2,3-butanediol channel (Pcar_0329). These two proteins may facilitate diffusion of glycerol and 1,3-propanediol, respectively. The Pcar_2508 gene encodes a DUF190 family protein that may modulate one or both channels. Three outer membrane proteins sharing 49-56% sequence identity are encoded by Pcar_1395 on the 3^′^ side of the glycerol dehydratase genes, by Pcar_2512, which is transcribed divergently from the 1,3-propanediol dehydrogenase gene, and by Pcar_3009. They may facilitate diffusion across the outer membrane for glycerol, 1,3-propanediol, and acetoin/2,3-butanediol, respectively. Yet another triad of paralogous genes (Pcar_1394, Pcar_2515, Pcar_2884; 55-62% sequence identity) linked to these gene clusters encodes radical SAM domain oxidoreductases whose substrates are unknown.

### Metabolism of 1,2-ethanediol

*P*. *carbinolicus* can grow by disproportionation of 1,2-ethanediol to ethanol plus acetate [1], yielding 0.5 ATP (Figure 4). However, its genome does not encode a three-subunit adenosylcobalamin-dependent diol dehydratase [41] to convert 1,2-ethanediol to acetaldehyde. The 1,2-ethanediol dehydratase of *P*. *carbinolicus* strains seems to be more oxygen-sensitive [36]; it may be a glycyl radical enzyme encoded by Pcar_0937 (34% identity to glycerol dehydratase Pcar_1397), and Pcar_0943 (38% identity to Pcar_1396) may encode its activating enzyme. The intervening genes are of uncertain relevance to 1,2-ethanediol metabolism (Additional file 3: Table S2). The reactions of glycerol and 1,2-ethanediol metabolism are missing from the published metabolic model of *P*. *carbinolicus*[10], which attributes a pyruvate formate-lyase function to both dehydratases on the basis of similarity to an *Escherichia coli* protein for which such a function could not be substantiated [42]. Experimental validation of 1,2-ethanediol dehydratase function will surely prove valuable.

### Metabolism of ethanolamine and choline

Duplicate genes encoding ethanolamine ammonia-lyase (*eutBC1* Pcar_0491, *eutBC2* Pcar_0467, 70% identical in sequence), each being an unusual fusion of the large and small subunits, were found in the genome of *P*. *carbinolicus*, strains of which grow by splitting ethanolamine or choline into ammonia or trimethylamine plus acetaldehyde, which is disproportionated to ethanol plus acetate (Figure 4) [36]. The duplication suggests that ethanolamine ammonia-lyase and choline trimethylamine-lyase may be distinct enzymes. The genes surrounding the two lyase genes are also duplicates, and encode periplasmic substrate-binding proteins (Pcar_0492, Pcar_0468) and multitransmembrane proteins (Pcar_0490, Pcar_0466) that may mediate uptake of ethanolamine or choline, as well as proteins of unknown function (Pcar_0493, Pcar_0469) of which a third paralog (Pcar_1023) is encoded next to one of the two ammonium transporters. The ethanolamine ammonia-lyase gene cluster consists of 43 genes transcribed in the same direction (Additional file 3: Table S2), and encodes one of the four predicted acetaldehyde:ferredoxin oxidoreductases (*aorA2* Pcar_0456).

The genes between *eutBC1* and *eutBC2* have functions in biosynthesis of cobalamin, the cofactor of ethanolamine ammonia-lyase (Additional file 3: Table S2). Some of them are seemingly redundant with genes elsewhere in the genome (Additional file 4: Table S3), and some have diverged considerably from their counterparts in *Geobacteraceae*. Notably, there is no *eutA* gene in the *P*. *carbinolicus* genome that encodes an ATPase that replaces damaged cobalamin within ethanolamine ammonia-lyase [43], and the gene for cobalt-precorrin-6A reductase (*cbiJ* Pcar_0470) has no homolog in any *Geobacter* genome except on the plasmid of *Geobacter lovleyi*. On the 3^′^ side of the *eutBC2* genes are genes encoding an uncharacterized metal ABC transporter related to transporters for corrinoids such as cobalamin, several ligand-binding proteins of the VWFA superfamily, an ATPase, and an outer membrane channel for cobalamin. The cobalt ABC transporter genes *cbiMNOQ* of *Geobacteraceae* have no homologs in *P*. *carbinolicus*, and it is not apparent how cobalt uptake may occur other than by this putative cobalamin transport system. Overall, the gene organization implies that *P*. *carbinolicus* may coordinate cobalamin uptake and biosynthesis with the need to metabolize ethanolamine and choline.

### Ethanol as product and substrate

Ethanol and acetate are the end products of fermentation of 2,3-butanediol, acetoin, 1,2-ethanediol, ethanolamine or choline. However, in the presence of S° as an electron acceptor or electron shuttle to Fe(III), or with a hydrogen/formate-consuming partner, *P*. *carbinolicus* can oxidize ethanol to acetate [1,44]. Remarkably, the enzymes predicted to interconvert ethanol, acetate and acetyl-CoA with acetaldehyde are each encoded by multiple genes in *P*. *carbinolicus* (Figure 4), but *Geobacter* species typically have one homolog per genome. This redundancy may reflect high flux through these reactions in *P*. *carbinolicus* in different growth modes.

Two putative ethanol dehydrogenases (Pcar_0251, Pcar_0255) are upregulated during growth by ethanol oxidation compared to ethanol-producing fermentation of acetoin [9], suggesting that they may be specialized for oxidation, along with a third isozyme (Pcar_1594) that shares 94-97% sequence identity with them. Two more isozymes (Pcar_2847, Pcar_2848), with 63% identity to each other and >54% identity to the first three, might be dedicated to ethanol production. Consistent with this hypothesis, the candidate ethanol dehydrogenases of ethanol-oxidizing *Geobacter* species (which do not produce ethanol) have greater sequence identity with the first three isozymes. During 2,3-butanediol oxidation, ethanol dehydrogenase is not required in either direction, but only two isozymes, Pcar_0251 and Pcar_0255, are downregulated (2.1-fold and 5.7-fold, respectively) compared to 2,3-butanediol fermentation (our unpublished microarray data). An ethanol dehydrogenase function has been hypothesized also for Pcar_2506 [10], but as noted above, this gene may be 3-hydroxypropanoate dehydrogenase.

Acetyl-CoA reductase has a catabolic function in 2,3-butanediol fermentation, when half of the acetyl-CoA produced by acetoin dehydrogenase is reduced to acetaldehyde and then to ethanol to dispose of electrons. This function is absent in acetoin fermentation because all electrons are donated to the acetaldehyde produced by acetoin dehydrogenase. Acetyl-CoA reductase also works in concert with acetaldehyde:ferredoxin oxidoreductase to maintain oxidized NAD and doubly reduced ferredoxin (Fd2e) at levels that drive catabolic reactions forward. During oxidation of ethanol or 2,3-butanediol to acetate and in the early stage of acetoin fermentation when acetoin is the electron acceptor instead of acetaldehyde [32], acetyl-CoA reductase functions oxidatively as acetaldehyde dehydrogenase. Of the three predicted acetyl-CoA reductase isozymes (49-57% sequence identity), Pcar_1246 is downregulated during 2,3-butanediol fermentation 4.5-fold compared to ethanol oxidation and 3.4-fold compared to 2,3-butanediol oxidation (our unpublished microarray data), suggesting that its function is oxidative; Pcar_2758 is upregulated during ethanol oxidation 7.2-fold compared to acetoin fermentation [9] and 4.2-fold compared to 2,3-butanediol fermentation, but downregulated during 2,3-butanediol oxidation 4.6-fold compared to 2,3-butanediol fermentation (our unpublished microarray data), indicating both oxidative and reductive roles; and the third isozyme, Pcar_2851, is not differentially expressed.

Four genes encode putative acetaldehyde:ferredoxin oxidoreductases (*aorA1* Pcar_0254, *aorA2* Pcar_0456, *aorA3* Pcar_0665, and *aorA4* Pcar_2853) sharing 72-79% sequence identity. Transcription of *aorA1*, *aorA2* and *aorA3* is predicted to be controlled by riboswitches that bind “molybdenum cofactor,” of which one variant is the cofactor of acetaldehyde:ferredoxin oxidoreductase, *bis*-(molybdopterin guanine dinucleotide)-tungsten. These three isozymes are differentially expressed according to the mode of growth: *aorA1* is upregulated during 2,3-butanediol fermentation 3.8-fold compared to 2,3-butanediol oxidation; *aorA2* is upregulated during ethanol oxidation 11.6-fold compared to 2,3-butanediol fermentation and 15.3-fold compared to acetoin fermentation [9]; and *aorA3* is upregulated during acetoin fermentation 2.9-fold compared to ethanol oxidation [9]. No riboswitch was found on the 5^′^ side of *aorA4*, which is not differentially expressed. Given the high sequence identity of these four isozymes and the presence of homologs in many *Geobacteraceae*, it seems incorrect that the acetaldehyde:ferredoxin oxidoreductase reaction was assigned to only one *aorA* in the metabolic model of *P*. *carbinolicus* and omitted from the metabolic models of *G*. *metallireducens* and *P*. *propionicus*[10].

The biochemical characterization of these enzymes, whose substrate specificity was predicted based on gene copy number and differential expression rather than sequence identity with characterized enzymes, is an important topic for future research.

### Oxidation of 1-propanol and 1-butanol

With acetate as a carbon source, *P*. *carbinolicus* can utilize 1-propanol and 1-butanol as electron donors, either by transferring hydrogen/formate to a syntrophic partner [1] or by using S° as an electron acceptor or shuttle to Fe(III) [44]. Of the two enzymes that were assigned a 1-propanol dehydrogenase function in the metabolic model [10], one (Pcar_0257) has homologs in *Geobacteraceae* that utilize 1-propanol but the other (Pcar_2510) is the 1,3-propanediol dehydrogenase described above. The two 1-butanol dehydrogenases (Pcar_1085, Pcar_1095) predicted from the *P*. *carbinolicus* genome [10] have 34% and 41% sequence identity, respectively, to the BdhA and BdhB isozymes of *Clostridium acetobutylicum*[45], but Pcar_1085 appears to be frameshifted. Following oxidation of 1-propanol and 1-butanol to propanal and butanal, oxidation to propanoyl-CoA and butanoyl-CoA may be catalyzed by the acetaldehyde dehydrogenases (Pcar_1246, Pcar_2758, Pcar_2851) and conversion to propanoyl-phosphate and butanoyl-phosphate by the phosphate acetyltransferases (Pcar_2542, Pcar_2850). Production of ATP by substrate-level phosphorylation is catalyzed by propanoate kinase (Pcar_2427) and butanoate kinase (Pcar_2852).

**Figure 5:**
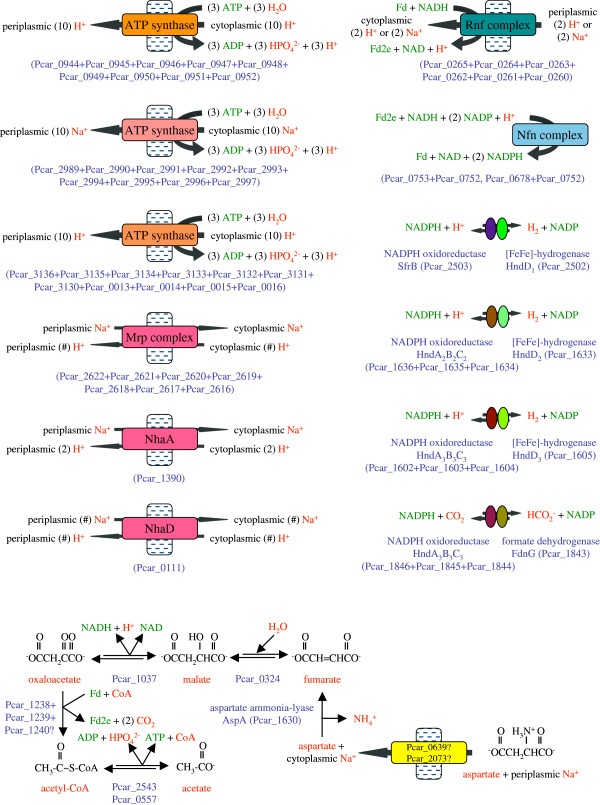
**The hydrogen/formate production pathway in *P*. *carbinolicus*.** ATP derived from substrate-level phosphorylation is used to generate transmembrane gradients of protons and sodium ions. This energy is used to exchange NADH for doubly reduced ferredoxin (Fd2e) so that NADH and Fd2e can be exchanged for NADPH, which donates electrons to hydrogenases and formate dehydrogenase. The lower part of the figure depicts how the aspartate ammonia-lyase encoded among hydrogenase assembly genes might function in an ATP-producing pathway with electrons released as hydrogen or formate. An uncharacterized 2-oxoacid:ferredoxin oxidoreductase complex is hypothesized to be a novel enzyme, oxaloacetate:ferredoxin oxidoreductase, analogous to enzymes that convert 2-oxoglutarate to succinyl-CoA and pyruvate to acetyl-CoA.

Partial oxidation of ethanol, 1-propanol or 1-butanol to acetate, propanoate or butanoate produces two NADH and one ATP. Growth of *P*. *carbinolicus* using these electron donors with hydrogen/formate transfer to a syntrophic partner implies that the energetic cost of exchange of two NADH for two hydrogen/formate molecules must be less than one ATP. The candidate enzymes for this process (Figure 5) are the ATP synthases that hydrolyze ATP to pump protons or sodium ions, the Rnf complex that exploits the transmembrane potential to reduce ferredoxin to Fd2e with electrons from NADH [46], the Nfn complex that exchanges one NADH plus one Fd2e for two NADPH [47], and NADPH oxidoreductases that form complexes with cytoplasmic [FeFe]-hydrogenases [48] or formate dehydrogenase. The overall pathway requires the Rnf complex to exchange one NADH for Fd2e by passing fewer protons or sodium ions than the number pumped by hydrolysis of one ATP. The number of protons or sodium ions pumped by hydrolysis of three ATP is equal to the number of C subunits in ATP synthase, which varies between 10 and 15 depending on the protein sequence of the C subunit [49]. The number of C subunits in each ATP synthase of *P*. *carbinolicus* is unknown, but can be estimated from the results of an experiment that compared the yield of *P*. *carbinolicus* cells with either ethanol or hydrogen as the electron donor, S° as an electron shuttle, and either soluble or insoluble Fe(III) as the terminal electron acceptor [9]. Assuming that the amount of energy expended per cell produced is invariant and that electron transfer from NADH to S° yields no energy, a comparison of the numbers of cells produced per Fe(III) reduced during growth on ethanol *versus* growth on hydrogen is a comparison of the energy yields that accompany reduction of NAD by oxidation of ethanol *versus* oxidation of hydrogen. The same [FeFe]-hydrogenases that are predicted to produce hydrogen from NADPH should also oxidize hydrogen and reduce NADP; the Nfn complex can function in reverse to exchange two NADPH for one NADH and one Fd2e; and the Rnf complex can function in reverse to produce a second NADH with electrons from Fd2e, pumping fewer protons or sodium ions than can be used to make one ATP. Oxidation of ethanol also produces two NADH but yields one ATP. Thus, the number of protons or sodium ions pumped by hydrolysis of one ATP (which is the number of C subunits divided by three), divided by the number of protons or sodium ions pumped by the Rnf complex, should be equal to the ratio of cell yields of *P*. *carbinolicus* with ethanol and with hydrogen. Growth of *P*. *carbinolicus* on ethanol compared to growth on hydrogen produced approximately 1.49 times as many cells per soluble Fe(III) reduced and 1.83 times as many cells per insoluble Fe(III) reduced [9]. The best fit to these data is a model in which hydrolysis of three ATP pumps ten protons or sodium ions and the Rnf complex pumps two protons or sodium ions as electrons pass from Fd2e to NAD. Thus, for each ethanol/1-propanol/1-butanol molecule oxidized to yield one ATP and requiring transfer of four electrons as hydrogen/formate to a syntrophic partner, the cell is predicted to expend 0.6 ATP to pump two protons or sodium ions that are returned through the Rnf complex, for a net energy yield of 0.4 ATP.

The next two sections will describe what the genome of *P*. *carbinolicus* reveals about its multiple ATP synthases, hydrogenases, formate dehydrogenase and associated NADPH oxidoreductases.

### ATP synthases and cation gradients

As *P*. *carbinolicus* derives ATP by substrate-level phosphorylation in every mode of growth except when it transfers electrons from hydrogen/formate to S° [44], its ATP synthases almost always function in reverse, generating proton or sodium ion gradients by ATP hydrolysis. *P*. *carbinolicus* has duplicate ancestral sets of ATP synthase genes and a third, acquired set [11]. Interestingly, in both ancestral sets, the B’ stalk subunit (*atpX* gene product) that communicates between the proton channel and the ATP-binding sites differs from those of *Geobacter* species; its predicted isoelectric point is 5.07, several pH units more acidic than those of *Geobacter* species, which range from 9.80 to 10.32. This difference may reflect the need to transport protons in opposite directions.

The three gene sets are in notable locations (Additional file 3: Table S2). One copy of the ancestral genes is divided into two divergent operons flanking the chromosomal origin of replication at distances of 9 kbp (the *atpX*_*3*_*F*_*3*_*H*_*3*_*A*_*3*_*G*_*3*_*D*_*3*_*C*_*3*_ operon) and 18 kbp (the *atpZIB*_*3*_*E*_*3*_ operon), locations that favour constitutively high expression. This may be important because the end product of *P*. *carbinolicus* metabolism is acetate, which can diffuse across the inner membrane and re-enter as acetic acid, dissipating the proton gradient [50]. This gene set includes the *atpZ* and *atpI* genes that are found in *Geobacteraceae* and implicated in import of magnesium [51].

The second ancestral copy (the *atpX*_*1*_*F*_*1*_*H*_*1*_*A*_*1*_*G*_*1*_*D*_*1*_*C*_*1*_*B*_*1*_*E*_*1*_ operon) is located next to the previously described CRISPR locus [12]. A sequence on the 5^′^ side of this operon (Pcar_R0094 in Additional file 2: Figure S1) contains overlapping predicted binding sites (consensus YTNACNNTTTTTTSAC, solid boxes in Figure S1) for the transcriptional regulator ColR [52], followed by inverted repeats (dotted boxes in Figure S1). It aligns with the Pcar_R0095 sequence located on the 5^′^ side of *eptA* (Pcar_1724), encoding lipid A phosphoethanolamine transferase, an enzyme that may modify the outer membrane to increase resistance to acid and cationic peptides [53]. ColR (Pcar_1726) and its cognate sensor kinase ColS (Pcar_1725) share 65% and 37% sequence identity, respectively, with homologs that regulate outer membrane modification in *Pseudomonas putida*[52]. Therefore, whenever the metabolism of *P*. *carbinolicus* causes acetic acid to accumulate in its surroundings, it is likely to use ColR signalling to impermeabilize the outer membrane and to overexpress ATP synthase so as to regulate the acidity of the periplasm.

The laterally acquired ATP synthase (encoded by the *atpD*_*2*_*C*_*2*_*QRB*_*2*_*E*_*2*_*F*_*2*_*A*_*2*_*G*_*2*_ operon) is of the N-type, which translocates sodium ions rather than protons [54]. These genes are transcribed divergently from a mechanosensitive ion channel gene (Pcar_2988), suggesting that expression of this ATP synthase may be controlled to ensure rapid restoration of the sodium gradient dissipated by opening of the channel. Compared to its homologs in *G*. *sulfurreducens* and *Geobacter metallireducens*, Pcar_2988 has an N-terminal extension of ~500 amino acid residues comprised of a predicted periplasmic domain and eight additional transmembrane segments of unknown function. Six other mechanosensitive channels are encoded by the genome of *P*. *carbinolicus* (Pcar_0306, Pcar_0812, Pcar_1009, Pcar_2451, Pcar_2469, and Pcar_2715), of which only Pcar_1009 has a homolog in any *Geobacter* species, in *Geobacter daltonii*. It might be valuable to investigate the roles of these ion channels in the physiology of *P*. *carbinolicus*.

Interestingly, all genes of the N-type ATP synthase operon are upregulated during fermentation of 2,3-butanediol compared to oxidation of either 2,3-butanediol or ethanol with Fe(III) as the electron acceptor (our unpublished microarray data), but are not upregulated during fermentation of acetoin. In contrast, several genes of the other two ATP synthases are downregulated during fermentation of 2,3-butanediol but not acetoin.

*P*. *carbinolicus* may interconvert the sodium ion and proton gradients using at least three antiporters (Additional file 3: Table S2): an Mrp complex of seven subunits with homologs in various species [55]; NhaA, with 52% sequence identity to an *E*. *coli* antiporter that is activated at high pH [56]; and NhaD, with 54% sequence identity to an *Alkalimonas amylolytica* anitporter that functions at high sodium ion concentrations and high pH [57]. *P*. *carbinolicus* may establish a potassium ion gradient by symport with a sodium ion through a Ktr transporter (Pcar_0086+Pcar_0085) with 37-38% sequence identity to characterized homologs in *B*. *subtilis*[58]. In contrast, potassium uptake in *Geobacteraceae* may occur through ATP-dependent Kdp and proton gradient-dependent Kup transporters, consistent with their growth at lower salt concentrations. The salt tolerance of *P*. *carbinolicus* may be due to production of an osmolyte, *N*-epsilon-acetyl-beta-lysine, by L-lysine 2,3-aminomutase (*ablA* Pcar_1401) followed by beta-lysine *N*-epsilon-acetyltransferase (*ablB* Pcar_1402), as in methanogens [59].

### Electron transfer to hydrogen and formate

The *P*. *carbinolicus* genome encodes three [FeFe]-hydrogenases (*hndD1* Pcar_2502; *hndD2* Pcar_1633; *hndD3* Pcar_1605) in different gene clusters (Additional file 3: Table S2), which may indicate their roles in various growth modes. Hydrogenase HndD-1, encoded near enzymes of the glycerol/1,3-propanediol oxidation pathway, may receive electrons from this pathway via an SfrB-like protein (Pcar_2503) as its NADPH-oxidizing partner. Hydrogenases HndD-2 and HndD-3 may each form a complex with a three-subunit NADPH oxidoreductase encoded next to them (*hndA*_*2*_*B*_*2*_*C*_*2*_, *hndA*_*3*_*B*_*3*_*C*_*3*_), as is the case in *Desulfovibrio fructosovorans*[48]. The hypothesis that HndD-1 has a different partner than HndD-2 and HndD-3 is consistent with the fact that HndD-2 and HndD-3 share 83% sequence identity with each other but only 41-43% with HndD-1. Two of the maturation factors required by [FeFe]-hydrogenases, namely the [FeFe]-cluster assembly scaffold GTPase HydF [60] and the cyanide/carbon monoxide ligand-forming enzyme HydG [61], are encoded by the *hndD2* gene cluster, but the third factor, HydE, hypothesized to synthesize the dithiolate ligand, is genetically triplicate (*hydE1* Pcar_1722 in its own operon; *hydE2* on the 3^′^ side of *hndD2*; *hydE3* on the 3^′^ side of *hndD3*) and may be unique to each hydrogenase.

The *hndD2* gene cluster encodes aspartate ammonia-lyase between *hydG* and *hydF*, suggesting that *P*. *carbinolicus* may coordinate disposal of electrons as hydrogen with use of aspartate as a nitrogen source. The fate of fumarate made by aspartate ammonia-lyase is uncertain because *P*. *carbinolicus* does not utilize fumarate as an electron donor [1] and has no homolog of the dicarboxylate exchange transporter that *Geobacter* species require to use fumarate as an electron acceptor and excrete succinate [62]. Nevertheless, *P*. *carbinolicus* possesses a sodium/dicarboxylate symporter (Pcar_2073) with 39% sequence identity to a *Staphylococcus aureus* protein that imports fumarate, succinate and malate [63], as well as a more distantly related homolog (Pcar_0639). If *P*. *carbinolicus* can take up aspartate through these transporters and degrade it to acetate plus hydrogen/formate to yield an estimated 0.7 ATP (Figure 5), it would explain the aspartate ammonia-lyase gene’s location in the *hndD2* gene cluster.

The *hndD*-*3* gene cluster encodes an oxidoreductase (Pcar_1606) with a CCG domain pair typical of enzymes with quinone and/or disulfide substrates. An enzyme with 69% sequence identity to this oxidoreductase is encoded by Pcar_0048 next to a transcriptional regulator of the ArsR family (Pcar_0047) and a membrane protein of the DUF318 family (Pcar_0049), which is encoded next to arsenate reductase in *G*. *sulfurreducens* and *G*. *metallireducens*. Moreover, proteins of unknown function that share 39% sequence identity (Pcar_2603, Pcar_2707) are encoded next to one of the two arsenate reductases of *P*. *carbinolicus* (Pcar_1772, Pcar_2602) and next to an NADPH oxidoreductase (*hndC*-*5* Pcar_2708) with ~75% sequence identity to the hydrogenase-associated HndC proteins. These arrangements suggest that electron transfer to arsenate by *P*. *carbinolicus* may be mechanistically similar to hydrogen production.

A third set of NADPH oxidoreductase subunit genes (*hndA*_*1*_*B*_*1*_*C*_*1*_) is located next to a formate dehydrogenase catalytic subunit gene (Additional file 3: Table S2). This gene cluster encodes neither a hydrogenase nor the iron-sulfur cluster-binding and cytochrome *b* subunits of formate dehydrogenase, but it encodes formate dehydrogenase biogenesis proteins as well as carbonic anhydrase and a putative formate transporter, implying that these gene products work in concert to extract carbon dioxide from cytosolic bicarbonate, reduce it to formate with electrons from NADPH, and excrete it, perhaps in exchange for periplasmic bicarbonate. Thus, the genome of *P*. *carbinolicus* explains its ability to dispose of electrons as either hydrogen or formate depending on the uptake capabilities of its syntrophic partner [64]. In the presence of S° as an electron acceptor or shuttle to Fe(III), *P*. *carbinolicus* utilizes hydrogen and formate as electron donors [44], which implies that these enzymes also function in reverse to produce NADPH.

A fourth NADPH oxidoreductase complex with fused subunits and an extra ferredoxin-like cluster (*hndA*_*4*_*BC*_*4*_) is encoded next to an iron-sulfur cluster-binding protein that resembles a noncatalytic portion of formate dehydrogenase (Additional file 3: Table S2). This putative complex may partner with the iron-sulfur-oxygen hybrid cluster protein encoded nearby (*hcp*-*1* Pcar_0837) to counter oxidative/nitrosative stress.

In addition to hydrogen and formate, *P*. *carbinolicus* may also dispose of electrons as carbon monoxide, as observed in *Desulfovibrio vulgaris*[65]. Two carbon monoxide dehydrogenases and their predicted pyridine nucleotide-disulfide oxidoreductase partners are encoded by the *P*. *carbinolicus* genome (Additional file 3: Table S2). One gene set is near the chromosomal origin of replication, suggestive of constitutively high expression.

### Electron transfer to S° and to the outer surface

**Figure 6:**
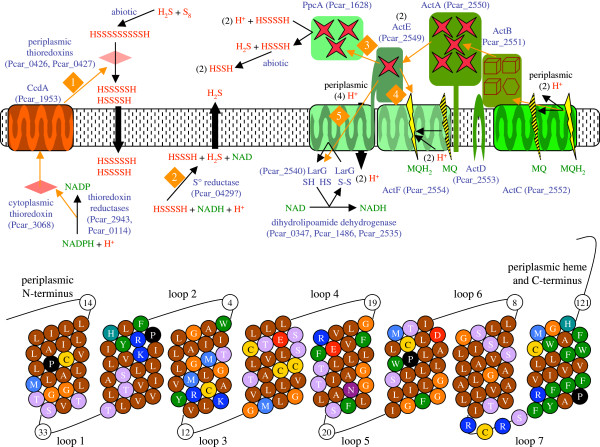
**Electron transfer pathways to S**° **and from menaquinol in *P*. *carbinolicus.*** Abiotic reaction with H_2_S may reduce sulfur (S_8_) to polysulfides. Electron transfer (orange arrows) may take various pathways (numbered) to reduce polysulfides to H_2_S. (**1**) Periplasmic thioredoxins that derive electrons from NADPH may reduce polysulfides to smaller, more soluble S° species. (**2**) A cytoplasmic S° reductase may extract individual sulfur atoms from a chain and reduce them to H_2_S with NADH. (**3**) Alternatively, S° may be reduced by PpcA, a periplasmic triheme *c*_*7*_-type cytochrome that receives electrons from menaquinol (MQH_2_) through the Act complex. Reduction of S° by MQH_2_ requires reverse electron transport, but the source of energy is unknown. The ActC subunit of the Act complex oxidizes MQH_2_, releasing two protons to the periplasm. In a Q loop, both electrons pass to iron-sulfur cluster-binding protein ActB, pentaheme *c*-type cytochrome ActA, monoheme *c*-type cytochrome ActE, and other *c*-type cytochromes such as PpcA. (**4**) In a Q cycle, only one electron takes this route while the other passes to menaquinone (MQ) with proton uptake from the cytoplasm, perhaps involving the ActF subunit. (**5**) ActE could form a channel lined with eight cysteine residues for reverse electron transport to a disulfide-based cytoplasmic electron carrier such as lipoyl carrier protein LarG. Dihydrolipoamide dehydrogenase reoxidizes dihydrolipoyl-LarG and reduces NAD. The bottom part of the figure depicts in one-letter amino acid code the transmembrane segments and one cytoplasmic loop of the unique ActE of *P*. *carbinolicus*.

Although *P*. *carbinolicus* is best known for fermentative and syntrophic growth, recent studies have offered clues regarding its use of S° as an electron acceptor and shuttle for electron transfer to Fe(III), and more details have emerged from the curated genome annotation. Electron transfer to S° is thought to involve two periplasmic thioredoxins (Pcar_0426, Pcar_0427), an outer membrane protein (Pcar_0428), and a cytoplasmic oxidoreductase (Pcar_0429) encoded by the most highly upregulated genes [9]. As the elemental form of S°, circular S_8_, is insoluble, it is thought to react extracellularly with sulfide, the end product of reduction, and to be reduced to linear polysulfides that are the true substrates of S° reductase [66]. The periplasmic thioredoxins might reduce polysulfides further until the molecules are small enough to diffuse into the cytoplasm (Figure 6). Periplasmic thioredoxins are reduced by CcdA (Pcar_1953), a membrane protein that receives electrons from cytoplasmic thioredoxin, reduced in turn by NADPH (Figure 6). When *P*. *carbinolicus* reduces S° with hydrogen as the electron donor [9], the use of NADP-reducing hydrogenases and the thioredoxin pathway would yield no energy. A more economical S° reductase must exist: either a cytoplasmic NADH-dependent enzyme (Pcar_0429) or a periplasmic *c*_*7*_-type cytochrome (*ppcA* Pcar_1628), which reduces S° in the related species *Desulfuromonas acetoxidans*[67]. Another role of CcdA is to reduce apocytochrome *c* disulfide reductase ResA (Pcar_1954), but although *resA* and *ccdA* are co-transcribed, only *ccdA* is upregulated during growth on S° [9], indicating a role for periplasmic electron carriers but not necessarily *c*-type cytochromes.

The enzyme encoded by Pcar_0429 has an FAD-dependent pyridine nucleotide-disulfide oxidoreductase domain, a persulfide-forming rhodanese-like domain, and two persulfide relay domains (TusA-like and DsrE-like). This combination suggests that the rhodanese-like domain displaces a sulfur atom from a substrate, forming a persulfide that is transferred to the TusA-like and DsrE-like domains, disulfide bond formation releases the sulfur atom as sulfide, and an electron pair transferred from NADH via FAD reduces the disulfide bond. If this cytoplasmic enzyme is the terminal S° reductase, it is likely to be peripherally associated with the inner membrane and oriented so that sulfide, a toxic product, is immediately protonated and diffuses outward (Figure 6).

When *P*. *carbinolicus* was first reported to express *c*-type cytochromes, four genes were predicted to encode its menaquinol:ferricytochrome *c* oxidoreductase [8]. Since that time, similar oxidoreductases encoded by *act* genes have been studied biochemically in *Rhodothermus marinus*[68] and *Chloroflexus aurantiacus*[69]. Six genes are now thought to encode the Act complex of *P*. *carbinolicus* (Figure 6, Additional file 3: Table S2). Oxidation of menaquinol typically releases protons to the periplasm, conserving energy as a proton gradient as electrons pass to *c*-type cytochromes. In a simple Q loop, both electrons are transferred to *c*-type cytochromes and the gradient generated is of one proton per electron. However, menaquinol:ferricytochrome *c* oxidoreductases may also perform a more complicated Q cycle in which one electron passes to *c*-type cytochromes while the other reduces another molecule of menaquinone with uptake of two protons from the cytoplasm, generating a gradient of two protons per electron. It is not known whether the Act complex operates a Q loop or a Q cycle. In some species, it has only one menaquinol-binding subunit, ActC, but in others including *P*. *carbinolicus* there is a second ActC-like subunit, ActF, with the potential to bind another molecule of menaquinone. In either case, electrons pass from menaquinol to *c*-type cytochromes at higher redox potential, and because the redox potential of S° is lower than that of menaquinone, *D*. *acetoxidans* and other species that use a *c*-type cytochrome to reduce S° must have a mechanism of reverse electron transport that has not yet been elucidated.

ActE, the monoheme cytochrome *c* subunit that may be present in two copies per Act complex as in *C*. *aurantiacus*[69], has eight predicted transmembrane segments in *P*. *carbinolicus* but only one in other species. This unique structure of ActE, potentially a channel lined with eight cysteine residues (Figure 6), suggests that the Act complex could transfer electrons from menaquinol not only to *c*-type cytochromes but to a disulfide-based electron carrier in the cytoplasm, a reverse electron transport process that could be driven by passage of protons through the channel (Figure 6). Nearby the *act* operon are genes that encode dihydrolipoamide dehydrogenase and a lipoyl carrier protein, LarG, similar to that of the glycine cleavage complex (Additional file 3: Table S2), indicating the existence of a pathway in which an electron pair reduces the disulfide bond of lipoyl-LarG, which is regenerated by reduction of NAD. Investigation of electron transfer and proton translocation by the Act complex in *P*. *carbinolicus* would improve the metabolic model and the understanding of Act complex diversity across species.

The number of predicted *c*-type cytochromes in *P*. *carbinolicus* now stands at sixteen (Additional file 5: Table S4): in addition to those identified previously [8], two gene products with corrected start sites (Pcar_1961, Pcar_2767) have signal peptides for translocation to the periplasm where heme is attached, and a sensor/regulator protein (Pcar_0181) is predicted to bind heme in its sensory domain. The designation of a glutamate synthase (Pcar_2944) as a *c*-type cytochrome by Haveman *et al*. is dubious because it is a cytoplasmic enzyme and the imagined heme-binding motif CXXCH within the flavin-binding domain is mutated to CXXCQ in other species. Although *c*-type cytochromes are few in *P*. *carbinolicus*, it has multiple cytochrome *c* biogenesis factors (Additional file 5: Table S4), as in *Geobacter* genomes [70], which may attach heme to different *c*-type cytochromes. Ligand-gated outer membrane channels (Pcar_0151, Pcar_0160, Pcar_0195), which perform active transport using the energy of the proton gradient transduced by periplasmic TonB-like proteins (Additional file 5: Table S4), are encoded near two *c*-type cytochrome genes (Pcar_0152, Pcar_0192), while TonB-like proteins (Pcar_2541, Pcar_0453, Pcar_0845, Pcar_2389, Pcar_2976) are encoded near the *act* genes and other outer membrane channel genes for uptake of cobalamin, Fe(III) and two unidentified solutes (Pcar_0454, Pcar_0852, Pcar_2397 and Pcar_2970, respectively). All seven ligand-gated channel genes are near genes for periplasmic metal-binding proteins (Pcar_0148, Pcar_0153, Pcar_2399) or molybdopterin-binding proteins (Pcar_0191, Pcar_0192) or riboswitches responsive to cobalamin and molybdopterin, indicating that they may transport metals. Three tetrapyrrole methyltransferases similar to those of cobalamin biosynthesis (Additional file 5: Table S4), one of which is also a *c*-type cytochrome, are encoded next to ligand-gated channels and may participate in biosynthesis of novel porphyrins that ligate metals other than Fe(II) and Co(II). Together, these features indicate that *P*. *carbinolicus* may employ *c*-type cytochromes in processes relevant to metals, although differently from its *Geobacter* relatives.

### Appendages and secretion systems

**Figure 7:**
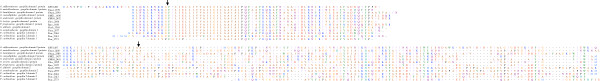
**Alignment of geopilin protein sequences.** In many *Geobacteraceae*, geopilins are split into two proteins that are thought to be translocated to the periplasm by separate systems and assembled there. Arrows indicate signal peptide cleavage sites: Pil-type for domain 1 and Sec-type for domain 2. In contrast, the duplicate geopilins and a membrane protein containing a geopilin-like sequence in *P*. *carbinolicus* each contain both domain 1 and domain 2, as do the geopilins of two *Geobacteraceae*.

*G*. *sulfurreducens* possesses metallic-like electroconductive pili [71] that are polymers of a unique subtype of type IVa pilin known as geopilin [72]. These pili enhance current production in fuel cells [73] and have been implicated in direct interspecies electron transfer within syntrophic aggregates [74,75]. *P*. *carbinolicus* does not produce current [76] and does not engage in direct interspecies electron transfer with syntrophic partners that have lost the ability to accept hydrogen and formate [64], but nevertheless possesses genes for several kinds of pili and other appendages that will be described in this section. Unlike the geopilin pilus biogenesis genes of *Geobacteraceae*, which occupy distant chromosomal locations, those of *P*. *carbinolicus* are found in one location (Additional file 5: Table S4). *P*. *carbinolicus* has only one set of genes for the minor components of the pilus (FimU, PilV, PilW, PilX, PilE) and the assembly factor PilY1, which are very different in sequence from the multiple versions in *G*. *sulfurreducens*, *G*. *metallireducens* and *Geobacter bemidjiensis* (not shown). Surprisingly, the geopilin gene is tandemly duplicated in *P*. *carbinolicus* and a geopilin-like sequence is part of another protein (Pcar_2773) predicted to have two transmembrane segments. Both geopilins of *P*. *carbinolicus* and Pcar_2773 contain both the conserved core domain 1 and the variable domain 2, which are split into two genes in many *Geobacteraceae* (Figure 7). One geopilin (Pcar_2144) that is upregulated during ethanol oxidation 3.0-fold relative to acetoin fermentation [9] and 6.6-fold relative to 2,3-butanediol fermentation (our unpublished microarray data) might increase the length of the pilus for attachment or electron transfer to the extracellular electron acceptor S°; the other geopilin gene adjacent to it (Pcar_2143) is not differentially expressed, nor is the Pcar_2773 gene. In contrast, the genes *pilE*, *pilM*, *pilN* and *pilQ* are upregulated 2.0-to-3.5-fold during 2,3-butanediol fermentation relative to oxidation of either 2,3-butanediol or ethanol (our unpublished microarray data), possibly adding more pilus biogenesis structures to the cell wall so that more numerous but shorter pili may be made from a lesser or equal supply of geopilin. Notably, these genes were not upregulated during acetoin fermentation. It would be interesting to study whether pili allow biofilms to form and insulate cells from ethanol, which is produced at a 1.5-fold higher level from 2,3-butanediol than from the less reduced substrate acetoin.

**Figure 8:**
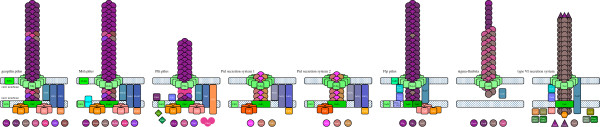
**Nonflagellar appendages and secretion systems of *P***. ***carbinolicus.*** The duplicate geopilins may assemble into the same pilus or different pili. Five minor pilins that assist in geopilin assembly may be incorporated into the pilus at intervals. The Msh pilus has one major pilin and five minor pilins. The Pih pilus has its own putative peptidase, PihH instead of PilD, three large minor pilins, and a major pilin without the motif cleaved by PilD (GFXXXE followed by a hydrophobic segment). Two type II Pul secretion systems may contain two or three pseudopilins each. The Flp pilus has its own putative peptidase, TadV, two major pilins and one minor pilin. The sigma-fimbria pilin CsuE is shorter than its orthologs, so the structural unit may be a heterodimer of CsuE and CsuF. The sigma-fimbria fibrillum may contain both adhesins together or one at a time. The type VI secretion system may incorporate both syringe proteins at once or one at a time.

*P*. *carbinolicus* also possesses unique type IVa pilus biogenesis systems that are not found in *Geobacter* genomes, called Msh and Pih (Figure 8, Additional file 5: Table S4). Several *msh* genes are upregulated during S° reduction compared to 2,3-butanediol fermentation, particularly the major pilin gene *mshA* (Pcar_0391) that is 5.1-fold higher during ethanol oxidation and 7.9-fold higher during 2,3-butanediol oxidation. *mshA* is the only *msh* gene upregulated (2.31-fold) during ethanol oxidation compared to acetoin fermentation [9]. Thus, the Msh pilus may promote utilization of the extracellular electron acceptor S°. The Pih pilus in both *P*. *carbinolicus* and *P*. *propionicus* is unusual in that the major pilin, PihA, lacks the motif recognized by the peptidase/methyltransferase PilD, suggesting that PihA is either translocated without subsequent cleavage or processed by a putative nonmethylating peptidase among the Pih proteins, PihH. Three minor components of the pilus (PihD, PihO, PihQ) retain the PilD recognition motif and are much larger than those of the other pili, underscoring the uniqueness of the Pih pilus.

Type IVa pili are evolutionarily related to type II secretion systems. One type II secretion system of *Geobacteraceae*, the Gsp system, is absent from *P*. *carbinolicus*, but the other, the Pul system that is required for secretion of OmpB, a laccase family multicopper oxidase with a role in the reduction of insoluble Fe(III) and Mn(IV) oxides [77], is entirely duplicated, including pseudopilins PulG, OxpG and TklG (Figure 8, Additional file 5: Table S4). There is no *ompB*-like gene in *P*. *carbinolicus*, but these systems may secrete other proteins. The proximity of both *pul* gene sets to genes of cell division and DNA uptake and metabolism is notable, as in *Geobacter* genomes.

*P*. *carbinolicus* also possesses genes for a more distantly related type IVb Flp pilus (Figure 8), in which the *flp* gene encoding the major pilin is duplicated, and for a sigma-fimbria [78] with duplicate adhesins (Additional file 5: Table S4). The Flp pilus biogenesis genes are all highly upregulated during 2,3-butanediol fermentation (our unpublished microarray data), with *flp1* expression increased 22.7-fold and 25.6-fold compared to 2,3-butanediol oxidation and ethanol oxidation, respectively, and *flp2* expression increased 27.2-fold compared to 2,3-butanediol oxidation. (Curiously, the only Flp pilus biogenesis gene not upregulated during 2,3-butanediol fermentation compared to ethanol oxidation is *flp2*, and none of the genes is upregulated during acetoin fermentation.) In contrast, decreased expression during 2,3-butanediol fermentation compared to oxidation of 2,3-butanediol or ethanol was observed for the sigma-fimbria adhesin genes *csuA* (7.3-fold and 11.2-fold, respectively) and *csuB* (4.4-fold and 2.3-fold, respectively) and chaperone gene *csuC* (2.5-fold and 3.0-fold, respectively). It would be interesting to study how different appendages contribute to the fitness of *P*. *carbinolicus* in growth modes with different substrates and products.

**Figure 9:**
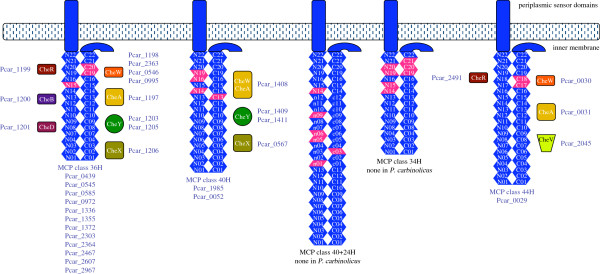
**Chemotaxis**-**like signalling systems in *P*. *carbinolicus.*** The cytoplasmic domain of each chemoreceptor (or MCP) is a coiled coil of amino acid residue heptads (blue, numbered), looped in the middle. The number of heptads and the positions of methylation sites (pink) are unique to each class of MCP. Methylation by CheR and demethylation by CheB and CheD modulate the ability of MCP to bind CheW (or CheV), which activates CheA. Phosphorylation by CheA and dephosphorylation by CheX cause CheY to inhibit or stimulate flagellar rotation and other processes. Twelve MCP of *P*. *carbinolicus* are of class 36H, in contrast with one per genome in *G*. *metallireducens*, *G*. *bemidjiensis*, *G*. *daltonii* and *Geobacter uraniireducens*, and none in *G*. *sulfurreducens*, *G*. *lovleyi* or *P*. *propionicus*. Most MCP of *Geobacteraceae* belong to classes 40H (nine to eighteen per genome), 40+24H (one to eight per genome), and 34H (two to eight per genome), but *P*. *carbinolicus* has only two of class 40H. *P*. *carbinolicus*, like its relatives except *G*. *metallireducens*, has one MCP gene of class 44H, predicted to be co-transcribed with a gene for outer membrane lipoprotein carrier/sorting protein LolA (Pcar_0032). These minor MCP appear to belong to rudimentary signalling systems that must rely on the methyltransferases and demethylases of the major MCP for fine-tuning.

*P*. *carbinolicus* possesses flagellar biogenesis genes (Additional file 5: Table S4), and has multiple flagellin genes *fliC* as do *G*. *bemidjiensis* and *G*. *lovleyi*. Both *fliC1* and *fliC2* are highly upregulated during ethanol oxidation compared to fermentation of 2,3-butanediol (20.9-fold and 12.9-fold, respectively; our unpublished microarray data) or acetoin (12.3-fold and 9.5-fold, respectively) [9], whereas *fliC3*, encoding a longer flagellin, is not differentially expressed. Flagellins of *P*. *carbinolicus* are likely to be glycosylated to impart a negative charge by enzymes encoded among the flagellar biogenesis genes. Different enzymes are encoded at the corresponding locations in *Geobacter* genomes. Flagellar motility may be controlled by a chemotactic signalling system encoded among flagellar biogenesis genes. Multiple chemotaxis-like signalling systems have been found in *Geobacter* genomes [79], but *P*. *carbinolicus* has only one complete and two rudimentary systems (Figure 9). The chemoreceptors called methyl-accepting chemotaxis proteins (MCP) associated with different systems are classified according to the number of heptads of amino acid residues in the cytoplasmic domain, which determines the locations of the methylation sites [80]. Twelve of the fifteen MCP of *P*. *carbinolicus* belong to class 36H (Figure 9), in contrast with at most one MCP of this class in *Geobacteraceae*. Conversely, MCP of classes 40H, 40+24H and 34H, which predominate in *Geobacteraceae*, are few or absent in *P*. *carbinolicus*, indicating near-total dissimilarity in chemotactic signalling.

The *P*. *carbinolicus* genome encodes four type V secretion systems in which a single protein called an autotransporter inserts its carrier domain into the outer membrane and extrudes its passenger domain (Additional file 5: Table S4). No autotransporters have been found in any *Geobacter* genome, indicating that the need for proteins that reach the outer surface by this process may be specific to *P*. *carbinolicus*. A part of the passenger domain of Pcar_0046 is a predicted cysteine peptidase, but the functions of the passenger domains are otherwise unknown. Autotransporter Pcar_1176 is 4.2-fold upregulated during 2,3-butanediol fermentation relative to oxidation of either 2,3-butanediol or ethanol (our unpublished microarray data).

The type VI secretion system of *P*. *carbinolicus* (Additional file 5: Table S4), whereby proteins may be injected into nearby bacterial or eukaryotic cells, consists of components that distantly resemble those of *Geobacteraceae*. Notably, the syringe protein TssI, which forms the tip of the needlelike appendage, has been duplicated in *P*. *carbinolicus*. Altogether, the appendages and secretion systems of *P*. *carbinolicus* appear more variegated than those of *Geobacteraceae*. Duplicated pilins, pseudopilins, adhesins, flagellins and syringe proteins may allow *P*. *carbinolicus* to present different features on the outer surface and to evolve these features rapidly.

### The defect in acetate oxidation

With S° as an electron acceptor or shuttle to Fe(III), *P*. *carbinolicus* excretes acetate instead of oxidizing it through the TCA cycle. Sun *et al*. have speculated that it does so because it lacks an unspecified ATP-driven reaction between succinate oxidation and S° reduction [10]. In more specific terms, succinate dehydrogenase of the TCA cycle reduces menaquinone, and Sun *et al*. modelled hydrogen and NADPH as the only electron donors to S°, which would require reverse electron transport. However, succinate oxidation with S° reduction was shown to be proton-gradient-dependent but NAD(P)-independent for *D*. *acetoxidans*[81], a relative of *P*. *carbinolicus*, and PpcA (Pcar_1628) is homologous to a periplasmic *c*_*7*_-type cytochrome of *D*. *acetoxidans* that reduces S° [67]. *P*. *carbinolicus* expresses PpcA specifically during growth with Fe(III), along with the Act complex pentaheme cytochrome subunit ActA [8], implying that it may perform reverse electron transport from menaquinol to a low-redox-potential *c*-type cytochrome that can reduce S°. As there is no evidence of a defect in reverse electron transport, one must consider what else could prevent acetate oxidation by *P*. *carbinolicus*.

The idea that any of the TCA cycle enzymes might be poorly active is disfavoured by the presence of two NAD-specific glutamate dehydrogenases in *P*. *carbinolicus*: GdhB (Pcar_1237) is 29% identical to a *Thermotoga maritima* enzyme [82] and GdhC (Pcar_1831) is 31% identical to a *Streptomyces clavuligerus* enzyme with multiple allosteric effectors [83]. The presence of these catabolic enzymes and not the NADP-specific GdhA of *Geobacteraceae* implies that *P*. *carbinolicus* has evolved to utilize glutamate as an electron donor, oxidizing 2-oxoglutarate through a partial TCA cycle to succinate to yield ATP. As there is no candidate transporter for *P*. *carbinolicus* to excrete succinate, further oxidation with reverse electron transport from menaquinol to an electron acceptor or syntrophic partner seems more likely. Excretion of fumarate, malate or oxaloacetate poses the same problem, but oxaloacetate might be oxidized by a 2-oxoacid:ferredoxin oxidoreductase of uncharacterized substrate specificity that is encoded next to *gdhB* (Pcar_1238+Pcar_1239+Pcar_1240), producing malonyl-CoA or acetyl-CoA. Thus, catabolic oxidation of glutamate would imply high activity of five of the eight TCA cycle enzymes, while the ability to make glutamate for biosynthetic purposes from other growth substrates suggests that the other three enzymes are also active. The presence of catabolic glutamate dehydrogenases and aspartate ammonia-lyase and the absence of known asparagine synthetases suggest that *P*. *carbinolicus* is accustomed to take up glutamate, aspartate and asparagine from its environment. If it operates the TCA cycle catabolically with oxaloacetate derived from these amino acids, its ability to make its own oxaloacetate may have diminished due to relaxed selective pressure.

During growth on any of its known substrates, *P*. *carbinolicus* must convert acetyl-CoA to oxaloacetate for biosynthetic purposes through pyruvate:ferredoxin/flavodoxin oxidoreductase (Por) and pyruvate carboxylase. If either of these reactions is too slow, excess oxaloacetate will not accumulate to a level that can sustain a catabolic TCA cycle. Both Por isozymes (Pcar_0377, Pcar_1034) and the pyruvate carboxylase (Pcar_1957) of *P*. *carbinolicus* share 68-75% sequence identity with their counterparts in *Geobacter* species and *D*. *acetoxidans*, which does not suggest major differences in activity, but interestingly, the ferredoxins and flavodoxins that could donate electrons to Por are very different between *P*. *carbinolicus* and its relatives (Additional file 6: Figure S2). Whereas *Geobacteraceae* have multiple single-4Fe4S-cluster ferredoxins similar to the preferred partner of Por in *Desulfovibrio africanus*[84], which suit the radical chemistry of acetyl-CoA reduction by Por by donating one electron at a time, *P*. *carbinolicus* has two double-4Fe4S-cluster ferredoxins that can carry two electrons, and six flavodoxin-like proteins. This difference suggests that electron transfer to Por from its ferredoxin/flavodoxin partners might be inefficient and limit production of oxaloacetate in *P*. *carbinolicus*, thereby preventing oxidation of acetate. This hypothesis offers a new direction for investigation of the unique metabolism of *P*. *carbinolicus*.

### Production of 2,3-butanediol from sugar substrates

**Figure 10:**
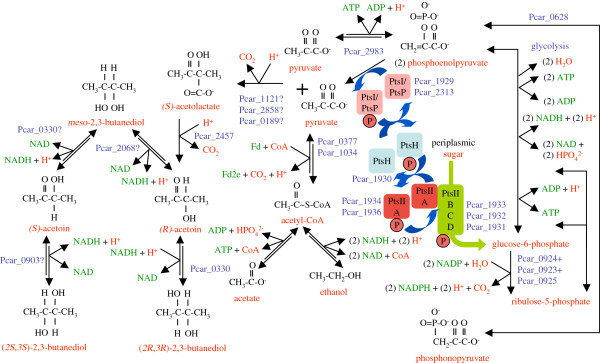
**Metabolism of sugars and 2,3-butanediol production by *P*. *carbinolicus.*** The phosphotransferase system (right) consists of two enzymes (PtsI, PtsP) that take a phosphate (circled P) from phosphoenolpyruvate, a phosphocarrier protein (PtsH), two signal output proteins (PtsII A), and a tripartite transporter complex (PtsII BCD) that imports and phosphorylates sugars yet to be identified. Glycolysis of a sugar (e.g. glucose-6-phosphate) yields NADH, ATP and two phosphoenolpyruvate molecules, which can either bring in two sugar molecules or yield two ATP or enter a third pathway through phosphonopyruvate (bottom right). The oxidative pentose-phosphate pathway from glucose-6-phosphate to ribulose-5-phosphate (which is recycled into glycolytic intermediates) yields no ATP, but by making NADPH instead of NADH, it may facilitate production of hydrogen/formate. If pyruvate:ferredoxin/flavodoxin oxidoreductase does not function catabolically in *P*. *carbinolicus* (middle), there will be insufficient acetyl-CoA for ethanol production to dispose of NADH from glycolysis, and the only source of doubly reduced ferredoxin (Fd2e) for hydrogen/formate production will be the Rnf complex. Production of acetoin and 2,3-butanediol (left), which *P*. *carbinolicus* can degrade to acetyl-CoA later, disposes of half the NADH from glycolysis.

The *P*. *carbinolicus* genome encodes an (*S*)-α-acetolactate decarboxylase (Pcar_2457) with 37% sequence identity to the characterized enzyme of *B*. *subtilis*[85], an indication that acetoin and 2,3-butanediol are not only growth substrates of *P*. *carbinolicus* but possibly end products of fermentation (Figure 10). (*S*)-α-acetolactate, a precursor of valine and leucine, can be made from two molecules of pyruvate by a biosynthetic-type synthase (Pcar_1910) with a regulatory subunit (Pcar_1909), but a fermentative pathway requires an additional catabolic-type (*S*)-α-acetolactate synthase. Three uncharacterized thiamin-dependent enzymes (Pcar_0189, Pcar_1121, Pcar_2858) are candidates for this function. It would be valuable to determine the functions of these three enzymes.

The presence of (*S*)-α-acetolactate decarboxylase is unexpected because none of the known growth substrates of *P*. *carbinolicus* is catabolized through pyruvate. (Glycolytic oxidation of glycerol is not possible in the absence of glycerol kinase.) An inability to ferment sugars through glycolysis is thought to be a defining characteristic of *Pelobacter* species [86]. Nevertheless, the *P*. *carbinolicus* genome encodes a full set of phosphotransferase system proteins for sugar uptake (Figure 10). In *Geobacteraceae* this phosphotransferase system is vestigial, comprised of signalling proteins orthologous to the two phosphoenolpyruvate--protein phosphotransferases PtsI (Pcar_1929) and PtsP (Pcar_2313), the phosphocarrier protein PtsH (Pcar_1930), and one or both signal output proteins IIA (Pcar_1934, Pcar_1936) of *P*. *carbinolicus*, plus a third protein IIA that is absent from *P*. *carbinolicus*. In addition to the signalling proteins, the *P*. *carbinolicus* genome encodes a set of sugar uptake proteins IIB, IIC and IID (Pcar_1933, Pcar_1932 and Pcar_1931, respectively) that have no homologs in *Geobacteraceae*. Interestingly, the *hprK* gene encoding a kinase/phosphatase to modulate PtsH activity is missing in *P*. *carbinolicus*, but an uncharacterized kinase (Pcar_1935) is encoded among phosphotransferase system components in *P*. *carbinolicus* and *Geobacteraceae*. It would be valuable to investigate whether any sugars can be taken up by *P*. *carbinolicus*.

The penultimate intermediate of glycolysis, phosphoenolpyruvate, carries a high-energy phosphate that may be used to activate an incoming sugar by the phosphotransferase system or to make ATP. In *P*. *carbinolicus*, but not in *Geobacter* species, phosphoenolpyruvate may also be rearranged to phosphonopyruvate by a phosphomutase (Pcar_0628) with 63% sequence identity to the characterized enzyme of *Mytilus edulis*[87], possibly to dispose of excess glycolytic intermediates when the ATP-to-ADP ratio is high. The synthetic and degradative polyphosphate kinases of *Geobacter* species are absent from *P*. *carbinolicus*, indicating an inability to transfer high-energy phosphates from excess ATP to a storage polymer. No characterized phosphonopyruvate decarboxylase has a homolog in *P*. *carbinolicus*; the assignment of an archaeal-type 3-phosphoglycerate mutase (Pcar_0824) to this function in the metabolic model [10] is doubtful. Speculatively, a 3-phosphoglycerate dehydrogenase-related protein (Pcar_0629) encoded by the same operon as the phosphomutase might reduce phosphonopyruvate to phosphonoglycerate, but it is not clear where this pathway leads.

Assuming that *P*. *carbinolicus* can convert a sugar substrate to glucose-6-phosphate, oxidize it to pyruvate, and make acetoin or 2,3-butanediol as end products, two questions arise: why would *P*. *carbinolicus* dispose of just half the NADH made from glycolysis by making 2,3-butanediol (or none by making acetoin) if it could dispose of all the NADH by making ethanol, and why does it possess an oxidative pentose-phosphate pathway to make NADPH when it can use the Nfn complex to exchange NADH from glycolysis plus Fd2e from Por for NADPH? The answer to both questions may be in the ability of Por to interact with ferredoxin. If electron transfer from Por to ferredoxin is too inefficient for catabolic oxidation of pyruvate to acetyl-CoA that can be reduced to ethanol - a hypothesis consistent with the observation that *P*. *carbinolicus* does not utilize pyruvate as a fermentative substrate [1,32], pyruvate from glycolysis would have to be converted to acetoin or 2,3-butanediol with the excess NADH converted via Fd2e and NADPH to hydrogen/formate. The Rnf complex can exchange NADH for Fd2e at an estimated cost of 0.6 ATP, but more energetically costly reactions may be required to ensure that NADH does not accumulate to inhibitory levels. (With non-sugar substrates, the four acetaldehyde:ferredoxin oxidoreductases fulfil this function.) The oxidative pentose-phosphate pathway essentially allows three carbon dioxide molecules and six NADPH to be made instead of pyruvate and NADH at a cost of one ATP. Thus, *P*. *carbinolicus* appears well-equipped to ferment sugars with a syntrophic partner.

*P*. *carbinolicus* possesses enzymes for degradation of 2-deoxyribose: if import via the phosphotransferase system activates this sugar to 2-deoxyribose-1-phosphate, it may be converted to 2-deoxyribose-5-phosphate by a phosphopentomutase (*deoB* Pcar_2320) with 41% sequence identity to the *E*. *coli* enzyme [88] and split into acetaldehyde plus glyceraldehyde-3-phosphate by a 2-deoxyribose-5-phosphate aldolase (*deoC* Pcar_2321) with 38% sequence identity to the *Mycoplasma pneumoniae* enzyme [89]. The predicted end products of this pathway are ethanol, acetate and 2,3-butanediol in a 1:1:1 ratio (although fermentative consumption of 2,3-butanediol may occur), yielding 1.5 ATP per molecule of 2-deoxyribose. The same enzymes may metabolize ribose, making glycolaldehyde instead of acetaldehyde. If any of the five ethanol dehydrogenases can reduce glycolaldehyde to 1,2-ethanediol, the end products would be 1,2-ethanediol and acetoin in a 2:1 ratio (both of which may be fermented further) and the yield would be 1 ATP per molecule of ribose. There is also a D-ribulose-1-phosphate/L-fuculose-1-phosphate aldolase (*fucA* Pcar_3030) with 38% sequence identity to the *E*. *coli* enzyme [90], by which *P*. *carbinolicus* could metabolize ribulose to glycolaldehyde plus glycerone-phosphate. The end products and ATP yield from ribulose would be the same as for ribose. Future studies should determine whether *P*. *carbinolicus* can metabolize these or other sugars without a syntrophic partner.

### Production of one-carbon units

**Figure 11:**
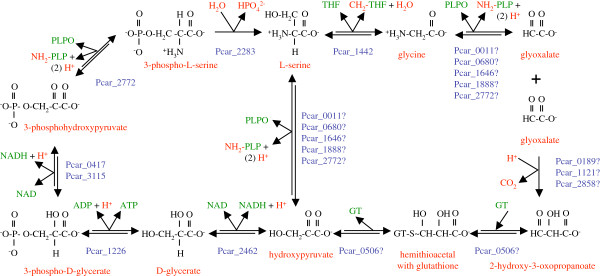
**One-carbon unit production in *P*. *carbinolicus*.** One-carbon units carried by tetrahydrofolate (THF) derive from serine, producing glycine. In the absence of the glycine cleavage complex, excess glycine might be deaminated to glyoxalate by aminotransferases using pyridoxal-5^′^-phosphate (PLPO). Fusion of two glyoxalate molecules by glyoxalate carboligase, rearrangement to hydroxypyruvate with the aid of glutathione (GT), and amination would regenerate serine. An alternative pathway involving phosphorylation allows hydroxypyruvate to enter glycolysis to be catabolized.

*Geobacteraceae* derive one-carbon units carried by tetrahydrofolate in two ways: from the hydroxymethyl group of serine as it is converted to glycine and by cleavage of excess glycine to release ammonia and carbon dioxide. *P*. *carbinolicus* has a serine hydroxymethyltransferase (Pcar_1442) but lacks the genes of the glycine cleavage system. Therefore, the genome of *P*. *carbinolicus* was searched for an alternative pathway to dispose of excess glycine. The discovery of glycerate 3-kinase (Pcar_1226) led to the hypothesis that two glycine molecules are deaminated, fused, and funnelled into glycolysis through glycerate (Figure 11). Three uncharacterized thiamin diphosphate-dependent enzymes (Pcar_0189, Pcar_1121 and Pcar_2858) are candidates for glyoxalate carboligase [91] to perform the fusion, and the fusion product is speculated to be rearranged to hydroxypyruvate by a hemithioacetal isomerase (Pcar_0506) with the aid of glutathione. Enzymes for glutathione synthesis have not been identified in *Geobacteraceae*, but *P*. *carbinolicus* possesses at least the first enzyme, gamma-glutamylcysteine synthetase (Pcar_3126), with 35% sequence identity to the characterized *Brassica juncea* enzyme [92]. Another hemithioacetal isomerase (Pcar_1477) has 56% sequence identity to the characterized *Neisseria meningitidis* enzyme [93] that serves to detoxify methylglyoxal, a byproduct of glycolysis formed by spontaneous dephosphorylation of glyceraldehyde-3-phosphate, by rearranging it to *S*-lactylglutathione. The presence of this enzyme in *P*. *carbinolicus* but not *Geobacteraceae* supports the idea that glycolysis is a catabolic pathway in *P*. *carbinolicus*. The Pcar_0506 hemithioacetal isomerase shares only 31% sequence identity with the *N*. *meningitidis* enzyme, so a different function such as isomerization of 2-hydroxy-3-oxopropanoate is plausible. *S*-lactylglutathione hydrolase (tentatively assigned to Pcar_3076 due to domain homology) is required to release lactate from glutathione, but isomerization of a hemithioacetal of 2-hydroxy-3-oxopropanoate should release glutathione automatically. If indeed *P*. *carbinolicus* is able to convert glycine to hydroxypyruvate, it should also be able to catabolize glycine taken up from its environment, as suggested by the presence of a putative glycine/alanine uptake transporter (Pcar_2492). Whereas hydroxypyruvate might be recycled to serine directly by various aminotransferases (Figure 11), recycling through glycerate 3-kinase costs 1 ATP, but allows hydroxypyruvate to enter a catabolic pathway of glycolysis. It will be interesting to study how *P*. *carbinolicus* disposes of excess glycine and what exogenous amino acids it can utilize.

## Conclusions

In this study, a curated genome annotation of *P*. *carbinolicus* was used to predict its metabolic pathways and physiological features. Candidate enzymes, some with structural innovations, were identified for catabolism of 2,3-butanediol, acetoin, glycerol, 1,2-ethanediol, ethanolamine, choline and ethanol, and newly predicted substrates: 1,3-propanediol, aspartate, glutamate and sugars. Pathways for energy transduction, electron transfer to S°, and production of hydrogen and formate were described. Remarkable features such as the mutant tRNA, cellular appendages and autotransporters were noted. The genome contents suggested that limited activity of Por could account for both the failure of *P*. *carbinolicus* to oxidize acetate through the TCA cycle and the presence of enzymes to ferment sugars to 2,3-butanediol rather than ethanol. Altogether, this work reveals that *P*. *carbinolicus* may be metabolically and physiologically more versatile than anticipated and possesses several unique features that deserve further investigation.

## Methods

### Sequence analysis and annotation

The genome of *P*. *carbinolicus* strain DSM 2380 (or Gra Bd 1) [1] was sequenced at the Joint Genome Institute (JGI) using a combination of 3 kb, 6 kb and 35 kb DNA libraries. Inserts were sequenced from both ends using the standard Sanger method. All three libraries provided 11x coverage of the genome. The Phred/Phrap/Consed software package ( http://www.phrap.com) was used for sequence assembly and quality assessment [94-96]. After the shotgun stage, 95,458 reads were assembled with parallel phrap (High Performance Software, LLC). Possible mis-assemblies were corrected with transposon bombing of bridging clones (Epicentre Biotechnologies, Madison, WI). Gaps between contigs were closed by editing in Consed, by custom primer walks, or by PCR amplification (Roche Applied Science, Indianapolis, IN). A total of 2484 additional reactions were necessary to close gaps and to raise the quality of the finished sequence. The completed genome sequence of *P*. *carbinolicus* DSM 2380 contains 97,942 reads, achieving an average of 11-fold sequence coverage per base with an error rate less than 1 in 100,000.

Genes were identified using two gene modeling programs, Glimmer [97] and Critica [98], as part of the Oak Ridge National Laboratory genome annotation pipeline. The two sets of gene calls were combined using Critica as the preferred start call for genes with the same stop codon. Genes with less than 80 amino acids that were predicted by only one of the gene callers and had no Blast hit in the KEGG database at 1e-05 were deleted. This was followed by a round of manual curation to eliminate obvious overlaps. The predicted CDSs were translated and used to search the National Center for Biotechnology Information (NCBI) nonredundant database, UniProt, TIGRFam, Pfam, PRIAM, KEGG, COG, and InterPro databases. These data sources were combined to assert a product description for each predicted protein. Non-coding genes and miscellaneous features were predicted using tRNAscan-SE [99], TMHMM [100] and SignalP [101].

### Manual curation

The automated genome annotation of *P*. *carbinolicus* and the manually curated genome annotations of *G*. *bemidjiensis*[102], *G*. *sulfurreducens* and *G*. *metallireducens*[70] were queried reciprocally with the protein BLAST algorithm [103] as implemented by OrthoMCL [104] using the default inflation parameter value (1.5), to identify mutual best hits as potential orthologs. The functional annotations of *P*. *carbinolicus* genes were emended for consistency with their counterparts in *G*. *bemidjiensis*, *G*. *sulfurreducens* and *G*. *metallireducens*. The coordinates of numerous genes were adjusted according to the criteria of full-length alignment, plausible ribosome-binding sites, and minimal overlap between genes on opposite DNA strands. The annotations of *P*. *carbinolicus* genes that were not matched to genes in *G*. *bemidjiensis*, *G*. *sulfurreducens* or *G*. *metallireducens* were checked by BLAST searches of NR and the Swiss-Prot database. Functional annotations were updated to match the experimental characterization of highly similar full-length homologs, with extensive reference to the EcoSal online textbook ( http://www.ecosal.org) and the MetaCyc database [105]. Genes that had no protein-level homologs in NR were checked (together with flanking intergenic sequences) by translated nucleotide BLAST in all six reading frames, and by nucleotide BLAST to ensure that conserved protein-coding or non-protein-coding features had not been missed. All intergenic regions of 30 bp or larger were also checked, which led to the annotation of numerous conserved nucleotide sequences. The curated annotation was submitted to the GenBank database (Accession No. CP000142.2).

## Abbreviations

ABC: ATP-binding cassette; ADP: Adenosine-5^′^-diphosphate; ATP: Adenosine-5^′^-triphosphate; ATPase: Adenosine-5^′^-triphosphate phosphohydrolase; CH_2_-THF: Methylenetetrahydrofolate; CoA: Coenzyme A; CRISPR: Clustered regularly interspaced short palindromic repeats; DNA: Deoxyribonucleic acid; FAD: Flavin adenine dinucleotide; Fd: Oxidized ferredoxin; Fd2e: Doubly reduced ferredoxin; GT: Glutathione; GTPase: Guanosine-5^′^-triphosphate phosphohydrolase; MCP: Methyl-accepting chemotaxis proteins; MDR: Medium-chain dehydrogenase/reductase; NAD(H): Nicotinamide adenine dinucleotide (reduced); NADP(H): Nicotinamide adenine dinucleotide 2^′^-phosphate (reduced); NH_2_-PLP: Pyridoxamine-5^′^-phosphate; PLPO: Pyridoxal-5^′^-phosphate; rRNA: Ribosomal RNA; RNA: Ribonucleic acid; SDR: Short-chain dehydrogenase/reductase; TCA: Tricarboxylic acid; THF: Tetrahydrofolate; tRNA: Transfer RNA; tRNA-Asn: tRNA for asparagine; tRNA-Leu: tRNA for leucine; UDP: Uridine-5^′^-diphosphate; VWFA: von Willebrand factor A.

## Competing interests

The authors declare that they have no competing interests.

## Authors' contributions

CH supervised the genome sequencing, OC performed genome sequence finishing, ML performed automated annotation, and PB provided bioinformatic support during curation. MA performed manual curation of the genome annotation, noted observations, and wrote the manuscript. SH and RD contributed to the curation and analysis of the genome and provided microarray data. The project was conceived and guided by DRL. All authors read, assisted with editing, and approved the final manuscript.

## Supplementary Material

Additional file 1 **Table S1.** Locations of multicopy nucleotide sequences in the *P.carbinolicus* genome. For exact coordinates and sequence alignments, see Additional file 2: Figure S1. Click here for file

Additional file 2 **Figure S1.** Alignments of multicopy nucleotide sequences of the *P.carbinolicus* genome. For the locus tags and functional annotations of adjacent genes, see Additional file 1: Table S1. Click here for file

Additional file 3 **Table S2.** Gene sets of *P.carbinolicus* for the catabolism of acetoin/2,3-butanediol, glycerol, 1,3-propanediol, 1,2-ethanediol, ethanolamine and choline, for proton/sodium pumping, hydrogen/formate production and electron transport. Click here for file

Additional file 4 **Table S3.** Thiamin and cobalamin biosynthesis genes of *P.carbinolicus.* Click here for file

Additional file 5 **Table S4.** Cytochrome *c* proteins and biogenesis factors, TonB-dependent transport systems, tetrapyrrole methyltransferases, and outer surface features of *P.carbinolicus:* genes for biogenesis of geopilin pili, Msh pili, Pih pili, type II secretion systems, Flp pili, sigma-fimbriae, flagella, autotransporters, and the type VI secretion system. Click here for file

Additional file 6 **Figure S2.** Alignments of ferredoxin and flavodoxin-like protein sequences of *P.carbinolicus* and its relatives. Ferredoxins of *D*. *africanus*, for which rates of electron transfer are known, and flavodoxins of *E*. *coli* and *D*. *africanus* are shown for comparison. Only double-4Fe4S-cluster ferredoxins have been detected in the partial genome sequence of *D*. *acetoxidans*, the closest relative of *P*. *carbinolicus*, which is able to operate a catabolic TCA cycle, but one of them has serine and aspartate in lieu of cysteine as two of the predicted ligands to iron atoms, and the other has two additional cysteine residues, which could affect their electron transfer properties. The existence of single-4Fe4S-cluster ferredoxins in *D*. *acetoxidans* cannot be ruled out. Click here for file

## References

[B1] Schink B Fermentation of 2,3-butanediol by *Pelobacter carbinolicus* sp. nov. and *Pelobacter propionicus* sp. nov., and evidence for propionate formation from C^2^ compounds Arch Microbiol 1984 137 1 33 41 10.1007/BF00425804

[B2] Krieg NR Holt JG Bergey’s manual of systematic bacteriology 1984 Baltimore: Williams and Wilkins

[B3] Holmes DE Nevin KP Lovley DR Comparison of 16S rRNA, *nifD*, *recA*, *gyrB*, *rpoB* and *fusA* genes within the family *Geobacteraceae* fam. nov Int J Syst Evol Microbiol 2004 54 Pt 5 1591 1599 15388715 10.1099/ijs.0.02958-0

[B4] Lonergan DJ Jenter HL Coates JD Phillips EJ Schmidt TM Lovley DR Phylogenetic analysis of dissimilatory Fe(III)-reducing bacteria J Bacteriol 1996 178 8 2402 2408 8636045 10.1128/jb.178.8.2402-2408.1996 PMC177952

[B5] Liesack W Finster K Phylogenetic analysis of five strains of gram-negative, obligately anaerobic, sulfur-reducing bacteria and description of *Desulfuromusa* gen. nov., including *Desulfuromusa kysingii* sp. nov., *Desulfuromusa bakii* sp. nov., and *Desulfuromusa succinoxidans* sp. nov Int J Syst Bacteriol 1994 44 753 758 10.1099/00207713-44-4-753

[B6] Lovley DR Lovley DR Fe(III) and Mn(IV) reduction Environmental Microbe–Metal Interactions 2000 Washington DC: American Society for Microbiology 3 29

[B7] Stackebrandt E Wehmeyer U Schink B The phylogenetic status of *Pelobacter acidigallici*, *Pelobacter venetianus*, and *Pelobacter carbinolicus* Syst Appl Microbiol 1989 11 257 260 10.1016/S0723-2020(89)80022-0

[B8] Haveman SA Holmes DE Ding YH Ward JE DiDonato RJ Jr Lovley DR c-Type cytochromes in *Pelobacter carbinolicus* Appl Environ Microbiol 2006 72 11 6980 6985 10.1128/AEM.01128-06 16936056 PMC1636167

[B9] Haveman SA DiDonato RJ Jr Villanueva L Shelobolina ES Postier BL Xu B Liu A Lovley DR Genome-wide gene expression patterns and growth requirements suggest that *Pelobacter carbinolicus* reduces Fe(III) indirectly via sulfide production Appl Environ Microbiol 2008 74 14 4277 4284 10.1128/AEM.02901-07 18515480 PMC2493185

[B10] Sun J Haveman SA Bui O Fahland TR Lovley DR Constraint-based modeling analysis of the metabolism of two *Pelobacter* species BMC Syst Biol 2010 4 174 10.1186/1752-0509-4-174 21182788 PMC3022650

[B11] Butler JE Young ND Lovley DR Evolution from a respiratory ancestor to fill syntrophic and fermentative niches: comparative fenomics of six *Geobacteraceae* species BMC Genomics 2009 10 103 10.1186/1471-2164-10-103 19284579 PMC2669807

[B12] Aklujkar M Lovley DR Interference with histidyl-tRNA synthetase by a CRISPR spacer sequence as a factor in the evolution of *Pelobacter carbinolicus* BMC Evol Biol 2010 10 230 10.1186/1471-2148-10-230 20667132 PMC2923632

[B13] Genschik P Billy E Swianiewicz M Filipowicz W The human RNA 3′-terminal phosphate cyclase is a member of a new family of proteins conserved in Eucarya, Bacteria and Archaea EMBO J 1997 16 10 2955 2967 10.1093/emboj/16.10.2955 9184239 PMC1169903

[B14] Tanaka N Shuman S RtcB is the RNA ligase component of an *Escherichia coli* RNA repair operon J Biol Chem 2011 286 10 7727 7731 10.1074/jbc.C111.219022 21224389 PMC3048659

[B15] Genschik P Drabikowski K Filipowicz W Characterization of the Escherichia coli RNA 3′-terminal phosphate cyclase and its sigma54-regulated operon J Biol Chem 1998 273 39 25516 25526 10.1074/jbc.273.39.25516 9738023

[B16] Okamoto A Kato R Masui R Yamagishi A Oshima T Kuramitsu S An aspartate aminotransferase from an extremely thermophilic bacterium, *Thermus thermophilus* HB8 J Biochem 1996 119 1 135 144 10.1093/oxfordjournals.jbchem.a021198 8907187

[B17] Min B Pelaschier JT Graham DE Tumbula-Hansen D Soll D Transfer RNA-dependent amino acid biosynthesis: an essential route to asparagine formation Proc Natl Acad Sci USA 2002 99 5 2678 2683 10.1073/pnas.012027399 11880622 PMC122407

[B18] Aubert JP Gavard R [Degradation metabolism of 2–3 butanediol and of acetoin by microorganisms; considerations on *Neisseria winogradskyi.* I. Investigations on 2–3 butanediol dehydrogenase] Ann Inst Pasteur (Paris) 1953 84 4 735 744 13124973

[B19] Stanier RY Fratkin SB Studies on the bacterial oxidation of 2,3-butanediol and related compounds Can J Res 1944 22b 5 140 153 10.1139/cjr44b-018

[B20] Taylor MB Juni E Stereoisomeric specificities of 2,3-butanediol dehydrogenases Biochim Biophys Acta 1960 39 448 457 10.1016/0006-3002(60)90197-9 13837186

[B21] Celinska E Grajek W Biotechnological production of 2,3-butanediol–current state and prospects Biotechnol Adv 2009 27 6 715 725 10.1016/j.biotechadv.2009.05.002 19442714

[B22] Ji XJ Huang H Ouyang PK Microbial 2,3-butanediol production: a state-of-the-art review Biotechnol Adv 2011 29 3 351 364 10.1016/j.biotechadv.2011.01.007 21272631

[B23] Hohn-Bentz H Radler F Bacterial 2,3-butanediol dehydrogenases Arch Microbiol 1978 116 2 197 203 10.1007/BF00406037 25056

[B24] Ui S Masuda T Masuda H Muraki H Mechanism for the formation of 2,3-butanediol stereoisomers in *Bacillus polymyxa* J Ferment Technol 1986 64 6 481 486 10.1016/0385-6380(86)90070-1

[B25] Yu B Sun J Bommareddy RR Song L Zeng AP Novel (2R,3R)-2,3-butanediol dehydrogenase from potential industrial strain *Paenibacillus polymyxa* ATCC 12321 Appl Environ Microbiol 2011 77 12 4230 4233 10.1128/AEM.02998-10 21531839 PMC3131630

[B26] Nicholson WL The *Bacillus subtilis ydjL (bdhA)* gene encodes acetoin reductase/2,3-butanediol dehydrogenase Appl Environ Microbiol 2008 74 22 6832 6838 10.1128/AEM.00881-08 18820069 PMC2583490

[B27] Yan Y Lee CC Liao JC Enantioselective synthesis of pure (R,R)-2,3-butanediol in *Escherichia coli* with stereospecific secondary alcohol dehydrogenases Org Biomol Chem 2009 7 19 3914 3917 10.1039/b913501d 19763290

[B28] Ui S Okajima Y Mimura A Kanai H Kobayashi T Kudo T Sequence analysis of the gene for and characterization of D-acetoin forming *meso*-2,3-butanediol dehydrogenase of *Klebsiella pneumoniae* expressed in Escherichia coli J Ferment Bioeng 1997 83 1 32 37 10.1016/S0922-338X(97)87323-0

[B29] Ui S Otagiri M Mimura A Dohmae N Takio K Ohkuma M Kudo T Cloning, expression and nucleotide sequence of the L-2,3-butanediol dehydrogenase gene from *Brevibacterium saccharolyticum* C-1012 J Ferment Bioeng 1998 86 290 295 10.1016/S0922-338X(98)80132-3

[B30] Otagiri M Ui S Takusagawa Y Ohtsuki T Kurisu G Kusunoki M Structural basis for chiral substrate recognition by two 2,3-butanediol dehydrogenases FEBS Lett 2010 584 1 219 223 10.1016/j.febslet.2009.11.068 19941855

[B31] Otagiri M Kurisu G Ui S Takusagawa Y Ohkuma M Kudo T Kusunoki M Crystal structure of *meso*-2,3-butanediol dehydrogenase in a complex with NAD+ and inhibitor mercaptoethanol at 1.7 A resolution for understanding of chiral substrate recognition mechanisms J Biochem 2001 129 2 205 208 10.1093/oxfordjournals.jbchem.a002845 11173520

[B32] Dubourguier HC Samain E Prensier G Albagnac G Characterization of two strains of *Pelobacter carbinolicus* isolated from anaerobic digesters Arch Microbiol 1986 145 248 253 10.1007/BF00443653

[B33] Oppermann FB Steinbuchel A Identification and molecular characterization of the aco genes encoding the *Pelobacter carbinolicus* acetoin dehydrogenase enzyme system J Bacteriol 1994 176 2 469 485 8110297 10.1128/jb.176.2.469-485.1994 PMC205071

[B34] Ali NO Bignon J Rapoport G Debarbouille M Regulation of the acetoin catabolic pathway is controlled by sigma L in *Bacillus subtilis* J Bacteriol 2001 183 8 2497 2504 10.1128/JB.183.8.2497-2504.2001 11274109 PMC95166

[B35] Kruger N Steinbuchel A Identification of *acoR*, a regulatory gene for the expression of genes essential for acetoin catabolism in *Alcaligenes eutrophus* H16 J Bacteriol 1992 174 13 4391 4400 1378052 10.1128/jb.174.13.4391-4400.1992 PMC206224

[B36] Eichler B Schink B Fermentation of primary alcohols and diols and pure culture of syntrophically alcohol-oxidizing anaerobes Arch Microbiol 1985 143 60 66 10.1007/BF00414769

[B37] Raynaud C Sarcabal P Meynial-Salles I Croux C Soucaille P Molecular characterization of the 1,3-propanediol (1,3-PD) operon of *Clostridium butyricum* Proc Natl Acad Sci USA 2003 100 9 5010 5015 10.1073/pnas.0734105100 12704244 PMC154289

[B38] Stines-Chaumeil C Talfournier F Branlant G Mechanistic characterization of the MSDH (methylmalonate semialdehyde dehydrogenase) from *Bacillus subtilis* Biochem J 2006 395 1 107 115 10.1042/BJ20051525 16332250 PMC1409689

[B39] Yuanyuan Z Yang C Baishan F Cloning and sequence analysis of the *dhaT* gene of the 1,3-propanediol regulon from *Klebsiella pneumoniae* Biotechnol Lett 2004 26 3 251 255 15049372 10.1023/b:bile.0000013715.04456.0a

[B40] Coppi MV O’Neil RA Leang C Kaufmann F Methe BA Nevin KP Woodard TL Liu A Lovley DR Involvement of *Geobacter sulfurreducens* SfrAB in acetate metabolism rather than intracellular, respiration-linked Fe(III) citrate reduction Microbiology 2007 153 Pt 10 3572 3585 17906154 10.1099/mic.0.2007/006478-0

[B41] Lee HA Jr Abeles RH Purification and properties of dioldehydrase, an enzyme requiring a cobamide coenzyme J Biol Chem 1963 238 2367 2373 13929077

[B42] Zhu J Shimizu K The effect of *pfl* gene knockout on the metabolism for optically pure D-lactate production by *Escherichia coli* Appl Microbiol Biotechnol 2004 64 3 367 375 10.1007/s00253-003-1499-9 14673546

[B43] Mori K Bando R Hieda N Toraya T Identification of a reactivating factor for adenosylcobalamin-dependent ethanolamine ammonia lyase J Bacteriol 2004 186 20 6845 6854 10.1128/JB.186.20.6845-6854.2004 15466038 PMC522198

[B44] Lovley DR Phillips EJ Lonergan DJ Widman PK Fe(III) and S^0^ reduction by *Pelobacter carbinolicus* Appl Environ Microbiol 1995 61 6 2132 2138 7793935 10.1128/aem.61.6.2132-2138.1995 PMC167486

[B45] Walter KA Bennett GN Papoutsakis ET Molecular characterization of two *Clostridium acetobutylicum* ATCC 824 butanol dehydrogenase isozyme genes J Bacteriol 1992 174 22 7149 7158 1385386 10.1128/jb.174.22.7149-7158.1992 PMC207405

[B46] Schmehl M Jahn A Meyer zu Vilsendorf A Hennecke S Masepohl B Schuppler M Marxer M Oelze J Klipp W Identification of a new class of nitrogen fixation genes in *Rhodobacter capsulatus*: a putative membrane complex involved in electron transport to nitrogenase Mol Gen Genet 1993 241 5–6 602 615 8264535 10.1007/BF00279903

[B47] Wang S Huang H Moll J Thauer RK NADP+ reduction with reduced ferredoxin and NADP+ reduction with NADH are coupled via an electron-bifurcating enzyme complex in *Clostridium kluyveri* J Bacteriol 2010 192 19 5115 5123 10.1128/JB.00612-10 20675474 PMC2944534

[B48] Malki S Saimmaime I De Luca G Rousset M Dermoun Z Belaich JP Characterization of an operon encoding an NADP-reducing hydrogenase in *Desulfovibrio fructosovorans* J Bacteriol 1995 177 10 2628 2636 7751270 10.1128/jb.177.10.2628-2636.1995 PMC176931

[B49] Pogoryelov D Reichen C Klyszejko AL Brunisholz R Muller DJ Dimroth P Meier T The oligomeric state of c rings from cyanobacterial F-ATP synthases varies from 13 to 15 J Bacteriol 2007 189 16 5895 5902 10.1128/JB.00581-07 17545285 PMC1952053

[B50] Baronofsky JJ Schreurs WJ Kashket ER Uncoupling by acetic acid limits growth of and acetogenesis by *Clostridium thermoaceticum* Appl Environ Microbiol 1984 48 6 1134 1139 16346677 10.1128/aem.48.6.1134-1139.1984 PMC241699

[B51] Hicks DB Wang Z Wei Y Kent R Guffanti AA Banciu H Bechhofer DH Krulwich TA A tenth *atp* gene and the conserved *atpI* gene of a *Bacillus atp* operon have a role in Mg2+ uptake Proc Natl Acad Sci USA 2003 100 18 10213 10218 10.1073/pnas.1832982100 12917488 PMC193541

[B52] Kivistik PA Kivi R Kivisaar M Horak R Identification of ColR binding consensus and prediction of regulon of ColRS two-component system BMC Mol Biol 2009 10 46 10.1186/1471-2199-10-46 19445690 PMC2689224

[B53] Lee H Hsu FF Turk J Groisman EA The PmrA-regulated *pmrC* gene mediates phosphoethanolamine modification of lipid A and polymyxin resistance in *Salmonella enterica* J Bacteriol 2004 186 13 4124 4133 10.1128/JB.186.13.4124-4133.2004 15205413 PMC421605

[B54] Dibrova DV Galperin MY Mulkidjanian AY Characterization of the N-ATPase, a distinct, laterally transferred Na+−translocating form of the bacterial F-type membrane ATPase Bioinformatics 2010 26 12 1473 1476 10.1093/bioinformatics/btq234 20472544 PMC2881411

[B55] Swartz TH Ikewada S Ishikawa O Ito M Krulwich TA The Mrp system: a giant among monovalent cation/proton antiporters? Extremophiles 2005 9 5 345 354 10.1007/s00792-005-0451-6 15980940

[B56] Taglicht D Padan E Schuldiner S Overproduction and purification of a functional Na+/H+ antiporter coded by *nhaA (ant)* from *Escherichia coli* J Biol Chem 1991 266 17 11289 11294 1645730

[B57] Liu J Xue Y Wang Q Wei Y Swartz TH Hicks DB Ito M Ma Y Krulwich TA The activity profile of the NhaD-type Na+(Li+)/H+ antiporter from the soda lake haloalkaliphile *Alkalimonas amylolytica* is adaptive for the extreme environment J Bacteriol 2005 187 22 7589 7595 10.1128/JB.187.22.7589-7595.2005 16267283 PMC1280297

[B58] Holtmann G Bakker EP Uozumi N Bremer E KtrAB and KtrCD: two K+ uptake systems in *Bacillus subtilis* and their role in adaptation to hypertonicity J Bacteriol 2003 185 4 1289 1298 10.1128/JB.185.4.1289-1298.2003 12562800 PMC142857

[B59] Pflüger K Baumann S Gottschalk G Lin W Santos H Muller V Lysine-2,3-aminomutase and beta-lysine acetyltransferase genes of methanogenic archaea are salt induced and are essential for the biosynthesis of Nepsilon-acetyl-beta-lysine and growth at high salinity Appl Environ Microbiol 2003 69 10 6047 6055 10.1128/AEM.69.10.6047-6055.2003 14532061 PMC201229

[B60] Shepard EM McGlynn SE Bueling AL Grady-Smith CS George SJ Winslow MA Cramer SP Peters JW Broderick JB Synthesis of the 2Fe subcluster of the [FeFe]-hydrogenase H cluster on the HydF scaffold Proc Natl Acad Sci USA 2010 107 23 10448 10453 10.1073/pnas.1001937107 20498089 PMC2890834

[B61] Nicolet Y Martin L Tron C Fontecilla-Camps JC A glycyl free radical as the precursor in the synthesis of carbon monoxide and cyanide by the [FeFe]-hydrogenase maturase HydG FEBS Lett 2010 584 19 4197 4202 10.1016/j.febslet.2010.09.008 20837009

[B62] Butler JE Glaven RH Esteve-Nunez A Nunez C Shelobolina ES Bond DR Lovley DR Genetic characterization of a single bifunctional enzyme for fumarate reduction and succinate oxidation in *Geobacter sulfurreducens* and engineering of fumarate reduction in *Geobacter metallireducens* J Bacteriol 2006 188 2 450 455 10.1128/JB.188.2.450-455.2006 16385034 PMC1347312

[B63] Hall JA Pajor AM Functional characterization of a Na(+)-coupled dicarboxylate carrier protein from *Staphylococcus aureus* J Bacteriol 2005 187 15 5189 5194 10.1128/JB.187.15.5189-5194.2005 16030212 PMC1196027

[B64] Rotaru A-E Shrestha PM Liu F Ueki T Nevin K Summers ZM Lovley DR Interspecies electron transfer via H2 and formate rather than direct electrical connections in co-cultures of *Pelobacter carbinolicus* and *Geobacter sulfurreducens* Appl Environ Microbiol 2012 78 21 7645 7651 10.1128/AEM.01946-12 22923399 PMC3485699

[B65] Voordouw G Carbon monoxide cycling by *Desulfovibrio vulgaris* Hildenborough J Bacteriol 2002 184 21 5903 5911 10.1128/JB.184.21.5903-5911.2002 12374824 PMC135394

[B66] Hedderich R Klimmek O Kröger A Dirmeier R Keller M Stetter KO Anaerobic respiration with elemental sulfur and with disulfides FEMS Microbiol Rev 1999 22 353 381

[B67] Pereira IA Pacheco I Liu MY Legall J Xavier AV Teixeira M Multiheme cytochromes from the sulfur-reducing bacterium *Desulfuromonas acetoxidans* Eur J Biochem 1997 248 2 323 328 10.1111/j.1432-1033.1997.00323.x 9346284

[B68] Pereira MM Refojo PN Hreggvidsson GO Hjorleifsdottir S Teixeira M The alternative complex III from *Rhodothermus marinus* - a prototype of a new family of quinol:electron acceptor oxidoreductases FEBS Lett 2007 581 25 4831 4835 10.1016/j.febslet.2007.09.008 17888426

[B69] Gao X Xin Y Bell PD Wen J Blankenship RE Structural analysis of alternative complex III in the photosynthetic electron transfer chain of *Chloroflexus aurantiacus* Biochemistry 2010 49 31 6670 6679 10.1021/bi100858k 20614874 PMC2914828

[B70] Aklujkar M Krushkal J DiBartolo G Lapidus A Land ML Lovley DR The genome sequence of *Geobacter metallireducens*: features of metabolism, physiology and regulation common and dissimilar to *Geobacter sulfurreducens* BMC Microbiol 2009 9 109 10.1186/1471-2180-9-109 19473543 PMC2700814

[B71] Malvankar NS Vargas M Nevin KP Franks AE Leang C Kim BC Inoue K Mester T Covalla SF Johnson JP Tunable metallic-like conductivity in microbial nanowire networks Nat Nanotechnol 2011 6 9 573 579 10.1038/nnano.2011.119 21822253

[B72] Reguera G McCarthy KD Mehta T Nicoll JS Tuominen MT Lovley DR Extracellular electron transfer via microbial nanowires Nature 2005 435 7045 1098 1101 10.1038/nature03661 15973408

[B73] Reguera G Nevin KP Nicoll JS Covalla SF Woodard TL Lovley DR Biofilm and nanowire production leads to increased current in *Geobacter sulfurreducens* fuel cells Appl Environ Microbiol 2006 72 11 7345 7348 10.1128/AEM.01444-06 16936064 PMC1636155

[B74] Malvankar NS Lovley DR Microbial nanowires: a new paradigm for biological electron transfer and bioelectronics ChemSusChem 2012 5 6 1039 1046 10.1002/cssc.201100733 22614997

[B75] Summers ZM Fogarty HE Leang C Franks AE Malvankar NS Lovley DR Direct exchange of electrons within aggregates of an evolved syntrophic coculture of anaerobic bacteria Science 2010 330 6009 1413 1415 10.1126/science.1196526 21127257

[B76] Richter H Lanthier M Nevin KP Lovley DR Lack of electricity production by *Pelobacter carbinolicus* indicates that the capacity for Fe(III) oxide reduction does not necessarily confer electron transfer ability to fuel cell anodes Appl Environ Microbiol 2007 73 16 5347 5353 10.1128/AEM.00804-07 17574993 PMC1950970

[B77] Mehta T Childers SE Glaven R Lovley DR Mester T A putative multicopper protein secreted by an atypical type II secretion system involved in the reduction of insoluble electron acceptors in *Geobacter sulfurreducens* Microbiology 2006 152 Pt 8 2257 2264 16849792 10.1099/mic.0.28864-0

[B78] Nuccio SP Baumler AJ Evolution of the chaperone/usher assembly pathway: fimbrial classification goes Greek Microbiol Mol Biol Rev 2007 71 4 551 575 10.1128/MMBR.00014-07 18063717 PMC2168650

[B79] Tran HT Krushkal J Antommattei FM Lovley DR Weis RM Comparative genomics of *Geobacter* chemotaxis genes reveals diverse signaling function BMC Genomics 2008 9 471 10.1186/1471-2164-9-471 18844997 PMC2577667

[B80] Alexander RP Zhulin IB Evolutionary genomics reveals conserved structural determinants of signaling and adaptation in microbial chemoreceptors Proc Natl Acad Sci USA 2007 104 8 2885 2890 10.1073/pnas.0609359104 17299051 PMC1797150

[B81] Paulsen J Kröger A Thauer RK ATP-driven succinate oxidation in the catabolism of *Desulfuromonas acetoxidans* Arch Microbiol 1986 144 78 83 10.1007/BF00454960

[B82] Kort R Liebl W Labedan B Forterre P Eggen RI de Vos WM Glutamate dehydrogenase from the hyperthermophilic bacterium *Thermotoga maritima*: molecular characterization and phylogenetic implications Extremophiles 1997 1 1 52 60 9680336 10.1007/s007920050014

[B83] Minambres B Olivera ER Jensen RA Luengo JM A new class of glutamate dehydrogenases (GDH). Biochemical and genetic characterization of the first member, the AMP-requiring NAD-specific GDH of *Streptomyces clavuligerus* J Biol Chem 2000 275 50 39529 39542 10.1074/jbc.M005136200 10924516

[B84] Pieulle L Charon MH Bianco P Bonicel J Petillot Y Hatchikian EC Structural and kinetic studies of the pyruvate-ferredoxin oxidoreductase/ferredoxin complex from *Desulfovibrio africanus* Eur J Biochem 1999 264 2 500 508 10.1046/j.1432-1327.1999.00648.x 10491097

[B85] Renna MC Najimudin N Winik LR Zahler SA Regulation of the *Bacillus subtilis alsS*, *alsD*, and *alsR* genes involved in post-exponential-phase production of acetoin J Bacteriol 1993 175 12 3863 3875 7685336 10.1128/jb.175.12.3863-3875.1993 PMC204803

[B86] Schink B Dworkin M, Falkow S, Rosenberg E, Schleifer K-H, Stackebrandt E The Genus *Pelobacter* The Prokaryotes 2006 3 Springer 5 11

[B87] Kim A Kim J Martin BM Dunaway-Mariano D Isolation and characterization of the carbon-phosphorus bond-forming enzyme phosphoenolpyruvate mutase from the mollusk *Mytilus edulis* J Biol Chem 1998 273 8 4443 4448 10.1074/jbc.273.8.4443 9468496

[B88] Hammer-Jespersen K Munch-Petersen A Phosphodeoxyribomutase from *Escherichia coli*. Purification and some properties Eur J Biochem 1970 17 3 397 407 10.1111/j.1432-1033.1970.tb01179.x 4992818

[B89] Loechel S Inamine JM Hu PC Nucleotide sequence of the *deoC* gene of *Mycoplasma pneumoniae* Nucleic Acids Res 1989 17 2 801 10.1093/nar/17.2.801 2492658 PMC331626

[B90] Lu Z Lin EC The nucleotide sequence of *Escherichia coli* genes for L-fucose dissimilation Nucleic Acids Res 1989 17 12 4883 4884 10.1093/nar/17.12.4883 2664711 PMC318048

[B91] Chang YY Wang AY Cronan JE Jr Molecular cloning, DNA sequencing, and biochemical analyses of *Escherichia coli* glyoxylate carboligase. An enzyme of the acetohydroxy acid synthase-pyruvate oxidase family J Biol Chem 1993 268 6 3911 3919 8440684

[B92] Hothorn M Wachter A Gromes R Stuwe T Rausch T Scheffzek K Structural basis for the redox control of plant glutamate cysteine ligase J Biol Chem 2006 281 37 27557 27565 10.1074/jbc.M602770200 16766527

[B93] Kizil G Wilks K Wells D Ala’Aldeen DA Detection and characterisation of the genes encoding glyoxalase I and II from *Neisseria meningitidis* J Med Microbiol 2000 49 7 669 673 10882093 10.1099/0022-1317-49-7-669

[B94] Ewing B Green P Base-calling of automated sequencer traces using phred. II. Error probabilities Genome Res 1998 8 3 186 194 9521922

[B95] Ewing B Hillier L Wendl MC Green P Base-calling of automated sequencer traces using phred. I. Accuracy assessment Genome Res 1998 8 3 175 185 9521921 10.1101/gr.8.3.175

[B96] Gordon D Abajian C Green P Consed: a graphical tool for sequence finishing Genome Res 1998 8 3 195 202 9521923 10.1101/gr.8.3.195

[B97] Delcher AL Bratke KA Powers EC Salzberg SL Identifying bacterial genes and endosymbiont DNA with Glimmer Bioinformatics 2007 23 6 673 679 10.1093/bioinformatics/btm009 17237039 PMC2387122

[B98] Badger JH Olsen GJ CRITICA: coding region identification tool invoking comparative analysis Mol Biol Evol 1999 16 4 512 524 10.1093/oxfordjournals.molbev.a026133 10331277

[B99] Lowe TM Eddy SR tRNAscan-SE: a program for improved detection of transfer RNA genes in genomic sequence Nucleic Acids Res 1997 25 5 955 964 9023104 10.1093/nar/25.5.955 PMC146525

[B100] Krogh A Larsson B von Heijne G Sonnhammer EL Predicting transmembrane protein topology with a hidden Markov model: application to complete genomes J Mol Biol 2001 305 3 567 580 10.1006/jmbi.2000.4315 11152613

[B101] Bendtsen JD Nielsen H von Heijne G Brunak S Improved prediction of signal peptides: SignalP 3.0 J Mol Biol 2004 340 4 783 795 10.1016/j.jmb.2004.05.028 15223320

[B102] Aklujkar M Young ND Holmes D Chavan M Risso C Kiss HE Han CS Land ML Lovley DR The genome of *Geobacter bemidjiensis*, exemplar for the subsurface clade of *Geobacter* species that predominate in Fe(III)-reducing subsurface environments BMC Genomics 2010 11 490 10.1186/1471-2164-11-490 20828392 PMC2996986

[B103] Altschul SF Gish W Miller W Myers EW Lipman DJ Basic local alignment search tool J Mol Biol 1990 215 3 403 410 2231712 10.1016/S0022-2836(05)80360-2

[B104] Li L Stoeckert CJ Jr Roos DS OrthoMCL: identification of ortholog groups for eukaryotic genomes Genome Res 2003 13 9 2178 2189 10.1101/gr.1224503 12952885 PMC403725

[B105] Caspi R Foerster H Fulcher CA Kaipa P Krummenacker M Latendresse M Paley S Rhee SY Shearer AG Tissier C The MetaCyc Database of metabolic pathways and enzymes and the BioCyc collection of Pathway/Genome Databases Nucleic Acids Res 2008 36 Database issue D623 D631 17965431 10.1093/nar/gkm900 PMC2238876

