# Supplementary material for: The genome of Pelobacter carbinolicus reveals surprising metabolic capabilities and physiological features
Source: BMC Genomics. 2012 Dec 10;13:690. doi: 10.1186/1471-2164-13-690 (PMC3543383; doi:10.1186/1471-2164-13-690)
Supplement: Additional file 1 — Table S1. Locations of multicopy nucleotide sequences in the P.carbinolicus genome. For exact coordinates and sequence alignments, see Additional file 2: Figure S1. [file 1471-2164-13-690-S1.pdf]

Additional file 1: Table S1. Locations of multicopy nucleotide sequences in the *P. carbinolicus* genome.

**Locus tag**	**Nearest genes**	**Annotations of nearest genes**
**Sequences containing putative ColR-binding sites**		
Pcar_R0094	3' of Pcar_3264<br>5' of Pcar_0944	hypothetical protein;<br>ATP synthase F0, B' subunit
Pcar_R0095	5' of Pcar_1724<br>3' of Pcar_1725	lipid A phosphoethanolamine transferase, putative;<br>sensor histidine kinase ColS (HAMP, HisKA, HATPase_c)
**5' regions of group II introns**		
Pcar_R0096	3' of Pcar_0652<br>5' of Pcar_0653	outer membrane channel, OmpJ-related;<br>winged-helix phosphate transcriptional response regulator (REC, trans_reg_C)
Pcar_R0097	3' of Pcar_0840<br>5' of Pcar_0841	conserved hypothetical protein;<br>RNA-directed DNA polymerase and maturase, group II intron origin
Pcar_R0098	5' of Pcar_3279<br>3' of Pcar_3281	hypothetical protein;<br>hypothetical protein
Pcar_R0099	3' of Pcar_1315<br>5' of Pcar_1316	ferritin-like domain protein;<br>RNA-directed DNA polymerase and maturase, group II intron origin
Pcar_R0100	3' of Pcar_R0026<br>5' of Pcar_1335	tRNA-Leu;<br>RNA-directed DNA polymerase and maturase, group II intron origin
Pcar_R0101	3' of Pcar_1372<br>5' of Pcar_1373	methyl-accepting chemotaxis sensory transducer, class 36H;<br>RNA-directed DNA polymerase and maturase, group II intron origin
Pcar_R0102	3' of Pcar_2865<br>3' of Pcar_2866	acetyltransferase, putative;<br>cytochrome *c* nitrite and sulfite reductase, catalytic subunit, 5 heme-binding sites
**3' regions of group II introns**		
Pcar_R0103	3' of Pcar_0841<br>5' of Pcar_0842	RNA-directed DNA polymerase and maturase, group II intron origin;<br>conserved hypothetical protein
Pcar_R0104	3' of Pcar_1335<br>5' of Pcar_3286	RNA-directed DNA polymerase and maturase, group II intron origin;<br>hypothetical protein
Pcar_R0105	3' of Pcar_1373<br>5' of Pcar_1374	RNA-directed DNA polymerase and maturase, group II intron origin;<br>DNA repair exonuclease SbcCD, D subunit, putative
Pcar_R0106	3' of Pcar_2865<br>3' of Pcar_2866	acetyltransferase, putative;<br>cytochrome *c* nitrite and sulfite reductase, catalytic subunit lipoprotein, 5 heme-binding sites
**Pcar_R10## family sequences and adjacent Pcar_R11## sequences**		

Pcar_R1001<br>Pcar_R1101<br>Pcar_R1002<br>Pcar_R1102<br>Pcar_R1003<br>Pcar_R1103<br>Pcar_R1004	3' of Pcar_0278<br>5' of Pcar_0279	tRNA (5-carboxymethylaminomethyl-2-seleno-U34)-2-selenouridine synthase;<br>protein of unknown function, DUF2779-containing
Pcar_R1005<br>Pcar_R1006<br>Pcar_R1007	5' of Pcar_0290<br>3' of Pcar_0291	sensor histidine kinase (Hist_Kin_Sens, PAS, PAS, HisKA, HATPase_c);<br>molybdopterin synthase, large subunit
Pcar_R1104<br>Pcar_R1008<br>Pcar_R1105	5' of Pcar_0291<br>5' of Pcar_0292	molybdopterin synthase, large subunit;<br>protein of unknown function, DUF2726 and zinc finger-containing
Pcar_R1009<br>Pcar_R1106	3' of Pcar_0293<br>5' of Pcar_3181	peptidylprolyl *cis-trans* isomerase, FKBP-type;<br>hypothetical protein
Pcar_R1010<br>Pcar_R1107<br>Pcar_R1011	3' of Pcar_0309<br>5' of Pcar_0310	oxidoreductase, aldo/keto reductase family;<br>nucleoside deaminase family protein
Pcar_R1012<br>Pcar_R1013	3' of Pcar_0314<br>5' of Pcar_0315	nitrogenase molybdenum-iron cofactor biosynthesis protein NifN;<br>nitrogenase molybdenum-iron cofactor biosynthesis protein NifUQ
Pcar_R1014<br>Pcar_R1108<br>Pcar_R1109<br>Pcar_R1015	3' of Pcar_0316<br>5' of Pcar_0318	menaquinone biosynthesis polyprenyltransferase, putative;<br>menaquinone biosynthesis decarboxylase, putative
Pcar_R1016	3' of Pcar_0320<br>5' of Pcar_0321	dehypoxanthinylfutalosine cyclase, putative;<br>histidinol-phosphate aminotransferase
Pcar_R1017	5' of Pcar_3203<br>3' of Pcar_0480	hypothetical protein;<br>cobalt-precorrin-8X methylmutase
Pcar_R1018	5' of Pcar_3230<br>3' of Pcar_0588	hypothetical protein;<br>tryptophanyl-tRNA synthetase
Pcar_R1019	5' of Pcar_1764<br>5' of Pcar_1765	heat shock protein Hsp90;<br>thioredoxin domain protein YyaL
Pcar_R1020	3' of Pcar_1772<br>3' of Pcar_1773	arsenate reductase and protein tyrosine phosphatase;<br>hypothetical protein
Pcar_R1021	3' of Pcar_2095<br>5' of Pcar_2096	carboxynorspermidine/carboxyspermidine dehydrogenase;<br>carboxynorspermidine/carboxyspermidine decarboxylase
Pcar_R1022	5' of Pcar_2294<br>5' of Pcar_2295	conserved hypothetical protein;<br>FAD-dependent pyridine nucleotide-disulfide oxidoreductase family protein
Pcar_R1023	5' of Pcar_2302<br>3' of Pcar_2303	conserved hypothetical protein;<br>methyl-accepting chemotaxis sensory transducer, class 36H

Pcar_R1024	3' of Pcar_2308<br>5' of Pcar_2309	glutamine amidotransferase, putative;<br>protein of unknown function DUF785
Pcar_R1110	5' of Pcar_2731<br>3' of Pcar_2732	DNA adenine methyltransferase, internal stop codon;<br>ribonucleoside triphosphate reductase-activating enzyme
Pcar_R1025	3' of Pcar_2941<br>3' of Pcar_2942	2-oxoglutarate dehydrogenase, E2 protein, dihydrolipoamide succinyltransferase;<br>molybdopterin synthase, large subunit
Pcar_1026<br>Pcar_1111<br>Pcar_1027<br>Pcar_1028	5' of Pcar_3061<br>3' of Pcar_3062	porphobilinogen synthase;<br>uroporphyrinogen III *C2*,*C7*-methyltransferase and uroporphyrinogen III synthase
**Pcar_R2### family sequences**		
Pcar_R2001	3' of Pcar_0218<br>3' of Pcar_3169	iron-molybdenum cluster-binding protein;<br>hypothetical protein
Pcar_R2002	5' of Pcar_0479<br>3' of Pcar_3203	radical SAM domain iron-sulfur cluster-binding oxidoreductase;<br>hypothetical protein
Pcar_R2003	3' of Pcar_0514<br>5' of Pcar_0515	SAM-dependent methyltransferase, type 11;<br>ion transport protein
Pcar_R2004	3' of Pcar_0783<br>5' of Pcar_0784	membrane protein, major facilitator superfamily;<br>transporter, RhtB family
Pcar_R2005	5' of Pcar_3259<br>5' of Pcar_0904	hypothetical protein;<br>tRNA dihydrouridine synthase-like flavin mononucleotide-binding domain protein
Pcar_R2006	5' of Pcar_1148<br>3' of Pcar_1149	flagellar hook-filament junction protein FlgL;<br>flagellar hook-associated protein FlgK
Pcar_R2007	3' of Pcar_1482<br>5' of Pcar_1483	NADPH-dependent glutamate synthase, glutamine amidotransferase and FMN-binding subunit;<br>NADPH-dependent glutamate synthase, NADPH oxidoreductase subunit
Pcar_R2008	3' of Pcar_1525<br>5' of Pcar_1526	undecaprenyl-diphospho-oligosaccharide flippase;<br>UDP-*N*-acetylglucosamine--lipopolysaccharide galacturonate 4-*N*-acetylglucosaminyltransferase, putative
Pcar_R2009	5' of Pcar_1649<br>3' of Pcar_1650	aminopeptidase N;<br>glutamyl-tRNA synthetase, non-discriminating
Pcar_R2010	5' of Pcar_1704<br>3' of Pcar_1705	zinc-dependent amidohydrolase, putative;<br>conserved hypothetical protein
Pcar_R2011	5' of Pcar_1762<br>3' of Pcar_1763	conserved hypothetical protein;<br>conserved hypothetical protein
Pcar_R2012	5' of Pcar_1865<br>3' of Pcar_1866	sodium-dependent transporter, SNF family;<br>conserved hypothetical protein
Pcar_R2013	5' of Pcar_2068<br>3' of Pcar_3465	*meso*-2,3-butanediol/*(2S,3S)*-2,3-butanediol dehydrogenase, putative;<br>hypothetical protein

Pcar_R2014	5' of Pcar_2305<br>3' of Pcar_3471	glycosyltransferase;<br>hypothetical protein
Pcar_R2015	3' of Pcar_2531<br>3' of Pcar_2532	ion channel, putative;<br>hydrolase, putative
Pcar_R2016	5' of Pcar_2735<br>3' of Pcar_2736	conserved hypothetical protein;<br>NUDIX hydrolase, ADP-ribose pyrophosphatase family
Pcar_R2017	3' of Pcar_3379<br>3' of Pcar_2759	hypothetical protein;<br>sensor histidine kinase (GAF, HisKA, HATPase_c)
Pcar_R2018	3' of Pcar_3379<br>3' of Pcar_2759	hypothetical protein;<br>sensor histidine kinase (GAF, HisKA, HATPase_c)
Pcar_R2019	5' of Pcar_2945<br>3' of Pcar_2946	isoprenoid biosynthesis amidotransferase-like protein ElbB;<br>helix-turn-helix transcriptional regulator with cupin domain
Pcar_R2020	3' of Pcar_3489<br>5' of Pcar_2969	hypothetical protein;<br>nitrilase/amidohydrolase superfamily protein, class 7
Pcar_R2021	3' of Pcar_3012<br>3' of Pcar_3013	flavin and coenzyme A sequestration protein dodecin;<br>membrane protein DUF81, putative
**Pcar_R3### family sequences**		
Pcar_R3001	5' of Pcar_0604<br>5' of Pcar_0605	membrane protein (UPF0126, UPF0126);<br>rhomboid-related membrane protein
Pcar_R3002	5' of Pcar_0883<br>5' of Pcar_3438	FeoA family protein;<br>conserved hypothetical protein
Pcar_R3003<br>Pcar_R3004	3' of Pcar_R0030<br>3' of Pcar_1432	tRNA-Arg;<br>nucleoside triphosphate pyrophosphohydrolase MazG
Pcar_R3005<br>Pcar_R3006	5' of Pcar_1673<br>3' of Pcar_1674	periplasmic carboxy-terminal processing protease lipoprotein;<br>zinc metalloendopeptidase M23 domain protein
Pcar_R3007	5' of Pcar_2559<br>5' of Pcar_2560	conserved hypothetical protein<br>sodium/phosphate symporter, putative
Pcar_R3008<br>Pcar_R3009	3' of Pcar_2960<br>5' of Pcar_2961	radical SAM domain iron-sulfur cluster-binding oxidoreductase, TIGR01212 family;<br>ATP-dependent DNA helicase Rep
**Pcar_R4### family sequences**		
Pcar_R4001	3' of Pcar_1739<br>5' of Pcar_1740	transcriptional repressor, LexA family;<br>DNA polymerase IV
Pcar_R4002<br>Pcar_R4003<br>Pcar_R4004	5' of Pcar_1769<br>3' of Pcar_1770	sulfate adenylyltransferase, subunit 2;<br>adenosine-5'-phosphosulfate reductase, glutathione-dependent
Pcar_R4005	3' of Pcar_1775<br>3' of Pcar_1776	VacJ family lipoprotein;<br>shikimate kinase
Pcar_R4006	5' of Pcar_1776<br>3' of Pcar_1777	shikimate kinase;<br>toxin, RelE family

Pcar_R4007<br>Pcar_R4008<br>Pcar_R4009<br>Pcar_R4010<br>Pcar_R4011<br>Pcar_R4012	5' of Pcar_3453<br>3' of Pcar_1779	DNA-binding protein of IS*Pca*10, N-terminal fragment;<br>phosphomannomutase
Pcar_R4013	5' of Pcar_1791<br>3' of Pcar_1792	transposase of IS*Pca*5, Y1_Tnp domain-containing;<br>phosphomannomutase
Pcar_R4014	5' of Pcar_1804<br>3' of Pcar_1805	UDP-glucose/UDP-*N*-acetylglucosamine 4-epimerase;<br>glutamine--fructose-6-phosphate aminotransferase
Pcar_R4015<br>Pcar_R4016<br>Pcar_R4017<br>Pcar_R4018	5' of Pcar_1805<br>3' of Pcar_3456	glutamine--fructose-6-phosphate aminotransferase;<br>UDP-glucose/UDP-*N*-acetylglucosamine 4-epimerase, C-terminal fragment
**Sequences of miscellaneous families (1)**		
Pcar_R5001	3' of Pcar_0161<br>5' of Pcar_0162	membrane protein, MarC family;<br>hypothetical protein
Pcar_R5002	5' of Pcar_3222<br>5' of Pcar_0542	hypothetical protein;<br>integron integrase
Pcar_R5003	5' of Pcar_1377<br>5' of Pcar_3445	integron integrase;<br>transposase of IS*Pca*3, IS481 family, N-terminal fragment
Pcar_R5004	5' of Pcar_3042<br>3' of Pcar_3043	hypothetical protein;<br>protein of unknown function DUF1847
Pcar_R5005	5' of Pcar_3409<br>3' of Pcar_3053	conserved hypothetical protein;<br>protein of unknown function DUF1847
**Sequences of miscellaneous families (2)**		
Pcar_R5006	3' of Pcar_0164<br>5' of Pcar_0165	hypothetical protein;<br>iron-sulfur cluster-binding protein, pyridoxine-5'-phosphate oxidase superfamily domain-containing
Pcar_R5007	3' of Pcar_2424<br>3' of Pcar_2425	hydrolase, putative;<br>transposase of IS*Pca*12
Pcar_R5008	5' of Pcar_2425<br>5' of Pcar_2426	transposase of IS*Pca*12;<br>conserved hypothetical protein
Pcar_R5009	3' of Pcar_2426<br>5' of Pcar_2427	conserved hypothetical protein;<br>propionate kinase, putative
Pcar_R5010	5' of Pcar_3048<br>3' of Pcar_3049	conserved hypothetical protein;<br>transposase of IS*Pca*14, Y1_Tnp domain-containing
**Sequences of miscellaneous families (3)**		
Pcar_R5011	3' of Pcar_0161<br>5' of Pcar_0162	membrane protein, MarC family;<br>hypothetical protein
Pcar_R5012	3' of Pcar_2424<br>3' of Pcar_2425	hydrolase, putative;<br>transposase of IS*Pca*12
Pcar_R5013	5' of Pcar_3042<br>3' of Pcar_3044	hypothetical protein;<br>conserved hypothetical protein

Pcar_R5014	5' of Pcar_3409<br>3' of Pcar_3053	conserved hypothetical protein;<br>protein of unknown function DUF1847
**Sequences of miscellaneous families (4)**		
Pcar_R5015	3' of Pcar_3159<br>5' of Pcar_0163	conserved hypothetical protein;<br>conserved hypothetical protein
Pcar_R5016	5' of Pcar_0529<br>3' of Pcar_0530	transposase of IS*Pca*6, IS4 family;<br>SAM-dependent methyltransferase, type 12
Pcar_R5017	5' of Pcar_0530<br>3' of Pcar_3212	SAM-dependent methyltransferase, type 12;<br>hypothetical protein
Pcar_R5018	5' of Pcar_3426<br>3' of Pcar_3222	hypothetical protein;<br>hypothetical protein
**Sequences of miscellaneous families (5)**		
Pcar_R5019	5' of Pcar_3177<br>5' of Pcar_0283	hypothetical protein;<br>conserved hypothetical protein
Pcar_R5020	5' of Pcar_3266<br>3' of Pcar_0980	hypothetical protein;<br>hypothetical protein
Pcar_R5021	5' of Pcar_2192<br>3' of Pcar_2193	shikimate 5-dehydrogenase;<br>NADH-dependent flavin oxidoreductase, Oye family, and FAD-dependent pyridine nucleotide-disulfide oxidoreductase family protein
Pcar_R5022	3' of Pcar_2494<br>5' of Pcar_3364	protein of unknown function DUF190;<br>hypothetical protein
**Sequences of miscellaneous families (6)**		
Pcar_R5023	5' of Pcar_3217<br>3' of Pcar_3218	hypothetical protein;<br>hypothetical protein
Pcar_R5024	5' of Pcar_3220<br>3' of Pcar_3426	hypothetical protein;<br>hypothetical protein
Pcar_R5025	5' of Pcar_3405<br>3' of Pcar_3041	hypothetical protein;<br>hypothetical protein
Pcar_R5026	5' of Pcar_3041<br>3' of Pcar_3491	hypothetical protein;<br>hypothetical protein
**Sequences of miscellaneous families (7)**		
Pcar_R5027	3' of Pcar_0531<br>5' of Pcar_0532	conserved hypothetical protein;<br>transposase of IS*Pca*4
Pcar_R5028	3' of Pcar_3297<br>5' of Pcar_1386	hypothetical protein;<br>metal-dependent phosphohydrolase, putative
Pcar_R5029	5' of Pcar_3491<br>3' of Pcar_3407	hypothetical protein;<br>hypothetical protein
**Sequences of miscellaneous families (8)**		
Pcar_R5030	5' of Pcar_3214<br>3' of Pcar_3215	hypothetical protein;<br>hypothetical protein
Pcar_R5031	5' of Pcar_3215<br>3' of Pcar_0533	hypothetical protein;<br>hypothetical protein
Pcar_R5032	5' of Pcar_0540<br>3' of Pcar_3220	DNA-binding protein of IS*Pca*10;<br>hypothetical protein

**Sequences of miscellaneous families (9)**		
Pcar_R5033	3' of Pcar_0983<br>3' of Pcar_0984	hypothetical protein;<br>EthD family protein
Pcar_R5034	5' of Pcar_0985<br>5' of Pcar_0986	protein of unknown function DUF159;<br>transposase of IS*Pca*1, IS4 family
Pcar_R5035	3' of Pcar_0987<br>5' of Pcar_0988	hypothetical protein;<br>chromosome partitioning ATPase Soj-related protein
**Sequences of miscellaneous families (10)**		
Pcar_R5036	3' of Pcar_3287<br>3' of Pcar_1341	hypothetical protein;<br>response regulator (REC)
Pcar_R5037	3' of Pcar_3365<br>5' of Pcar_2495	hypothetical protein;<br>RNA 3'-phosphate cyclase, class II
Pcar_R5038	3' of Pcar_2865<br>3' of Pcar_2866	acetyltransferase, putative;<br>cytochrome *c* nitrite and sulfite reductase, catalytic subunit lipoprotein, 5 heme-binding sites
**Sequences of miscellaneous families (11)**		
Pcar_R5039	3' of Pcar_1353<br>3' of Pcar_1354	conserved hypothetical protein;<br>cation-translocating P-type ATPase
Pcar_R5040	5' of Pcar_1354<br>3' of Pcar_1355	cation-translocating P-type ATPase;<br>methyl-accepting chemotaxis sensory transducer, class 36H
Pcar_R5041	3' of Pcar_2494<br>5' of Pcar_3364	protein of unknown function DUF190;<br>hypothetical protein
**Sequences of miscellaneous families (12)**		
Pcar_R5042	5' of Pcar_1785<br>3' of Pcar_1786	transposase of IS*Pca*5, Y1_Tnp domain-containing;<br>antitoxin, XRE family
Pcar_R5043	5' of Pcar_1787<br>5' of Pcar_1788	toxin, RelE family;<br>transposase of IS*Pca*9, ISL3 family
Pcar_R5044	5' of Pcar_3320<br>3' of Pcar_3321	transposase of IS*Pca*5, Y1_Tnp domain-containing, N-terminal fragment;<br>transposase of IS*Pca*5, Y1_Tnp domain-containing, N-terminal fragment
**Sequences of miscellaneous families (13)**		
Pcar_R5045	5' of Pcar_2644<br>3' of Pcar_3376	transposase of IS*Pca*6, IS4 family, frameshifted;<br>hypothetical protein
Pcar_R5046	5' of Pcar_3403<br>3' of Pcar_3039	conserved hypothetical protein;<br>conserved hypothetical protein
Pcar_R5047	5' of Pcar_3407<br>3' of Pcar_3492	hypothetical protein;<br>hypothetical protein
**Sequences of miscellaneous families (14)**		
Pcar_R5048	3' of Pcar_0283<br>5' of Pcar_3423	conserved hypothetical protein;<br>conserved hypothetical protein, N-terminal fragment
Pcar_R5049	5' of Pcar_3490<br>5' of Pcar_3400	transposase of IS*Pca*14, Y1_Tnp domain-containing, N-terminal fragment, frameshifted;<br>hypothetical protein

**Sequences of miscellaneous families (15)**		
Pcar_R5050	5' of Pcar_3216<br>3' of Pcar_0535	conserved hypothetical protein;<br>conserved hypothetical protein
Pcar_R5051	5' of Pcar_3039<br>3' of Pcar_3040	conserved hypothetical protein;<br>protein of unknown function DUF2321
**Sequences of miscellaneous families (16)**		
Pcar_R5052	3' of Pcar_3237<br>3' of Pcar_0791	hypothetical protein;<br>ATP/GTP-binding transposition helper
Pcar_R5053	3' of Pcar_R0034<br>3' of Pcar_3448	tRNA-Pro;<br>transposase of IS*Pca*7, C-terminal fragment
**Sequences of miscellaneous families (17)**		
Pcar_R5054	5' of Pcar_0809<br>3' of Pcar_R0073	conserved hypothetical protein;<br>tRNA-Thr, fragment
Pcar_R5055	5' of Pcar_0811<br>3' of Pcar_R0074	flagellin;<br>tRNA-Thr, fragment
**Sequences of miscellaneous families (18)**		
Pcar_R5056	5' of Pcar_3439<br>5' of Pcar_0913	transposase, C-terminal fragment<br>NADH oxidoreductase, HoxE-like subunit, putative
Pcar_R5057	3' of Pcar_2494<br>5' of Pcar_3364	protein of unknown function DUF190;<br>hypothetical protein
**Sequences of miscellaneous families (19)**		
Pcar_R5058<br>Pcar_R5059	3' of Pcar_0965<br>5' of Pcar_0972	CRISPR-associated endoribonuclease Cas2;<br>methyl-accepting chemotaxis sensory transducer, class 36H, Cache_1 domain-containing
**Sequences of miscellaneous families (20)**		
Pcar_R5060	3' of Pcar_1343<br>5' of Pcar_1344	response receiver sensor diguanylate cyclase/phosphodiesterase (REC, PAS, GAF, PAS, PAS, PAS, GGDEF, EAL);<br>ADP-ribosyl-glycohydrolase superfamily protein
Pcar_R5061	5' of Pcar_1354<br>3' of Pcar_1355	cation-translocating P-type ATPase;<br>methyl-accepting chemotaxis sensory transducer, class 36H
**Sequences of miscellaneous families (21)**		
Pcar_R5062	3' of Pcar_1472<br>5' of Pcar_1473	conserved hypothetical protein;<br>transposase of IS*Pca*11, Y1_Tnp domain-containing
Pcar_R5063	5' of Pcar_2731<br>3' of Pcar_2732	DNA adenine methyltransferase, internal stop codon;<br>ribonucleoside triphosphate reductase-activating enzyme
**Sequences of miscellaneous families (22)**		
Pcar_R5064	3' of Pcar_1472<br>5' of Pcar_1473	conserved hypothetical protein;<br>transposase of IS*Pca*11, Y1_Tnp domain-containing
Pcar_R5065	3' of Pcar_1473<br>5' of Pcar_1474	transposase of IS*Pca*11, Y1_Tnp domain-containing;<br>hypothetical protein
**Sequences of miscellaneous families (23)**		
Pcar_R5066	3' of Pcar_3314	hypothetical protein;

	3' of Pcar_1735	ABC transporter, ATP-binding protein
Pcar_R5067	3' of Pcar_2842<br>3' of Pcar_2843	protein of unknown function MJ1255;<br>hydroperoxide peroxidase OsmC
**Sequences of miscellaneous families (24)**		
Pcar_R5068<br>Pcar_R5069	5' of Pcar_2280<br>3' of Pcar_2281	universal stress protein Usp;<br>protein of unknown function DUF795
**Sequences of miscellaneous families (25)**		
Pcar_R5070	5' of Pcar_3035<br>5' of Pcar_3036	hypothetical protein;<br>pyrophosphatase PpaX, putative
Pcar_R5071	3' of Pcar_3036<br>3' of Pcar_3037	pyrophosphatase PpaX, putative;<br>conserved hypothetical protein, N-terminal fragment, frameshifted
**Sequences of miscellaneous families (26)**		
Pcar_R5072	5' of Pcar_3042<br>3' of Pcar_3043	hypothetical protein;<br>protein of unknown function DUF1847
Pcar_R5073	5' of Pcar_3409<br>3' of Pcar_3053	conserved hypothetical protein;<br>protein of unknown function DUF1847
**Sequences of miscellaneous families (27)**		
Pcar_R5074	5' of Pcar_3043<br>3' of Pcar_3044	protein of unknown function DUF1847;<br>conserved hypothetical protein
Pcar_R5075	5' of Pcar_3053<br>3' of Pcar_3054	protein of unknown function DUF1847;<br>conserved hypothetical protein
**Sequences of miscellaneous families (28)**		
Pcar_R5076	5' of Pcar_3044<br>3' of Pcar_3045	conserved hypothetical protein;<br>diguanylate cyclase/phosphodiesterase (GGDEF, EAL)
Pcar_R5077	5' of Pcar_3054<br>3' of Pcar_3055	conserved hypothetical protein;<br>diguanylate cyclase/phosphodiesterase (PAS, PAS, PAS, GGDEF, EAL)
**Pcar_R6### family sequences**		
Pcar_R6001<br>Pcar_R6002<br>Pcar_R6003	3' of Pcar_1480<br>5' of Pcar_1481	translation initation factor-related protein YciH;<br>conserved hypothetical protein
Pcar_R6004<br>Pcar_R6005<br>Pcar_R6006<br>Pcar_R6007	3' of Pcar_1481<br>5' of Pcar_1482	conserved hypothetical protein;<br>NADPH-dependent glutamate synthase, glutamine amidotransferase and FMN-binding subunit
**Heptanucleotide repeats Pcar_R70## and adjacent Pcar_71##/Pcar_R72## sequences**		
Pcar_R7001	5' of Pcar_0087<br>3' of Pcar_0088	excinuclease ABC, A subunit;<br>4-hydroxythreonine-4-phosphate dehydrogenase
Pcar_R7201<br>Pcar_R7202<br>Pcar_R7002	3' of Pcar_0266<br>3' of Pcar_0267	glutamate-1-semialdehyde 2,1-aminomutase;<br>ABC transporter, membrane protein
Pcar_R7003	3' of Pcar_0359<br>3' of Pcar_0360	ATP-dependent chaperone ClpB;<br>winged-helix transcriptional response regulator

		(REC, trans_reg_C)
Pcar_R7203	3' of Pcar_0431<br>5' of Pcar_0432	DNA-binding ATPase Uup;<br>membrane protein of unknown function DUF2238
Pcar_R7209<br>Pcar_R7101<br>Pcar_R7004<br>Pcar_R7102	5' of Pcar_0473<br>3' of Pcar_0474	cobalt-precorrin-4 *C11*-methyltransferase;<br>cobalt-precorrin-6B *C5*,*15*-methyltransferase, CbiE subunit, putative
Pcar_R7204<br>Pcar_R7103<br>Pcar_R7005<br>Pcar_R7104	5' of Pcar_0476<br>3' of Pcar_0477	cobalt-sirohydrochlorin *C20*-methyltransferase;<br>conserved hypothetical protein
Pcar_R7205<br>Pcar_R7107	5' of Pcar_0478<br>3' of Pcar_0479	sirohydrochlorin cobaltochelatase, putative;<br>radical SAM domain iron-sulfur cluster-binding oxidoreductase
Pcar_R7206<br>Pcar_R7105<br>Pcar_R7006<br>Pcar_R7106	5' of Pcar_0482<br>3' of Pcar_0483	cob(I)yrinate *a*,*c*-diamide adenosyltransferase;<br>adenosylcobinamide-phosphate synthase
Pcar_R7007	3' of Pcar_0507<br>3' of Pcar_0508	cardiolipin synthase, putative;<br>hemerythrin family protein
Pcar_R7008	5' of Pcar_0606<br>3' of Pcar_0607	membrane protein;<br>carboxythiazole phosphate tautomerase
Pcar_R7009	3' of Pcar_0942<br>5' of Pcar_0943	winged-helix DNA-binding domain protein;<br>1,2-ethanediol dehydratase-activating enzyme, putative
Pcar_R7010	5' of Pcar_1052<br>3' of Pcar_1053	conserved hypothetical protein;<br>phosphoribosylglycinamide formyltransferase, formate-dependent
Pcar_R7210	3' of Pcar_1070<br>3' of Pcar_1071	iron-sulfur cluster-binding oxidoreductase;<br>nicotinate phosphoribosyltransferase
Pcar_R7011	3' of Pcar_1107<br>3' of Pcar_1108	response receiver-modulated diguanylate cyclase (REC, GGDEF);<br>PilZ domain protein
Pcar_R7012<br>Pcar_R7013	5' of Pcar_1152<br>3' of Pcar_1153	flagellar rod-binding protein FlgJ;<br>flagellar P-ring protein FlgI
Pcar_R7014	5' of Pcar_1153<br>3' of Pcar_1154	flagellar P-ring protein FlgI;<br>flagellar L-ring lipoprotein FlgH
Pcar_R7015	3' of Pcar_1360<br>3' of Pcar_1361	FAD-dependent oxidoreductase, BBE domain-containing;<br>ABC transporter, ATP-binding protein, duplicated domain
Pcar_R7016	3' of Pcar_1374<br>5' of Pcar_1375	DNA repair exonuclease SbcCD, D subunit, putative;<br>DNA repair exonuclease SbcCD, C subunit, putative
Pcar_R7017	5' of Pcar_1738<br>5' of Pcar_1739	ABC transporter, periplasmic substrate-binding protein;

		transcriptional repressor, LexA family
Pcar_R7018<br>Pcar_R7019	3' of Pcar_1740<br>5' of Pcar_1741	DNA polymerase IV;<br>DNA polymerase III, alpha subunit
Pcar_R7207	5' of Pcar_1766<br>3' of Pcar_1767	phosphoenolpyruvate carboxykinase, GTP-dependent;<br>nitrite/sulfite reductase domain protein
Pcar_R7020	3' of Pcar_1772<br>3' of Pcar_1773	arsenate reductase and protein tyrosine phosphatase;<br>hypothetical protein
Pcar_R7021	3' of Pcar_1780<br>3' of Pcar_1781	conserved hypothetical protein;<br>mannose-1-phosphate guanylyltransferase and mannose-6-phosphate isomerase, C-terminal fragment
Pcar_R7022	3' of Pcar_2106<br>5' of Pcar_2107	ABC transporter, ATP-binding protein;<br>nitrogenase molybdenum-iron cofactor biosynthesis radical SAM domain iron-sulfur cluster-binding oxidoreductase
Pcar_R7023	3' of Pcar_2162<br>3' of Pcar_2163	membrane protein, putative;<br>conserved hypothetical protein
Pcar_R7024	5' of Pcar_2231<br>3' of Pcar_2232	phosphoribosylamine--glycine ligase;<br>phosphoribosylaminoimidazolecarboxamide formyltransferase and IMP cyclohydrolase
Pcar_R7025	3' of Pcar_2369<br>3' of Pcar_2370	RNA methyltransferase, TrmA family;<br>cold shock DNA/RNA-binding protein
Pcar_R7026	3' of Pcar_2374<br>5' of Pcar_2375	ABC transporter, membrane protein;<br>ABC transporter, ATP-binding protein
Pcar_R7027	3' of Pcar_2612<br>3' of Pcar_2613	flavodoxin, putative;<br>3'-to-5' exonuclease, putative
Pcar_R7208	5' of Pcar_3487<br>5' of Pcar_2853	hypothetical protein;<br>aldehyde:ferredoxin oxidoreductase, tungsten-containing
Pcar_R7028<br>Pcar_R7029	5' of Pcar_2943<br>3' of Pcar_2944	thioredoxin reductase;<br>ferredoxin-dependent glutamate synthase
Pcar_R7030	3' of Pcar_3108<br>3' of Pcar_3109	protein of unknown function DUF500;<br>protein of unknown function UPF0153
**Octanucleotide repeat-containing Pcar_R80## and adjacent Pcar_81## sequences**		
Pcar_R8001<br>Pcar_R8002	3' of Pcar_0063<br>3' of Pcar_0064	hypothetical protein;<br>peptidase, U32 family
Pcar_R8003<br>Pcar_R8004	3' of Pcar_0415<br>3' of Pcar_0416	NADH pyrophosphatase;<br>alanine dehydrogenase
Pcar_R8005<br>Pcar_R8006	5' of Pcar_0543<br>3' of Pcar_0544	peptidoglycan transglycosylase and transpeptidase PbpC;<br>alpha-2-macroglobulin domain protein
Pcar_R8007<br>Pcar_R8008	3' of Pcar_0948<br>5' of Pcar_0949	ATP synthase F1, gamma subunit;<br>ATP synthase F1, beta subunit
Pcar_R8009<br>Pcar_R8010	5' of Pcar_1470<br>5' of Pcar_1471	hypothetical protein;<br>nitrite/sulfite reductase domain protein

Pcar_R8011<br>Pcar_R8012	5' of Pcar_1715<br>3' of Pcar_1716	metal ion efflux pump, RND family, membrane fusion protein;<br>metal ion efflux pump, RND family, outer membrane protein
Pcar_R8013<br>Pcar_R8014<br>Pcar_R8015<br>Pcar_R8016<br>Pcar_R8017<br>Pcar_R8018	3' of Pcar_1739<br>5' of Pcar_1740	transcriptional repressor, LexA family;<br>DNA polymerase IV
Pcar_R8019<br>Pcar_R8020<br>Pcar_R8021<br>Pcar_R8101<br>Pcar_R8022<br>Pcar_R8023<br>Pcar_R8102<br>Pcar_R8024	3' of Pcar_1765<br>3' of Pcar_1766	thioredoxin domain protein YyaL;<br>phosphoenolpyruvate carboxykinase, GTP-dependent
Pcar_R8025<br>Pcar_R8026	5' of Pcar_1769<br>3' of Pcar_1770	sulfate adenylyltransferase, subunit 2;<br>adenosine-5'-phosphosulfate reductase, glutathione-dependent
Pcar_R8027<br>Pcar_R8028<br>Pcar_R8029<br>Pcar_R8030	5' of Pcar_3452<br>5' of Pcar_1772	hypothetical protein;<br>arsenate reductase and protein tyrosine phosphatase
Pcar_R8031<br>Pcar_R8103<br>Pcar_R8032<br>Pcar_R8033<br>Pcar_R8104<br>Pcar_R8034	3' of Pcar_1775<br>3' of Pcar_1776	VacJ family lipoprotein;<br>shikimate kinase
Pcar_R8035<br>Pcar_R8036	5' of Pcar_1776<br>3' of Pcar_1777	shikimate kinase;<br>toxin, RelE family
Pcar_R8037<br>Pcar_R8038<br>Pcar_R8039<br>Pcar_R8105<br>Pcar_R8040	5' of Pcar_3453<br>3' of Pcar_1779	DNA-binding protein of IS*Pca*10, N-terminal fragment;<br>phosphomannomutase
Pcar_R8041<br>Pcar_R8106<br>Pcar_R8042	5' of Pcar_1791<br>3' of Pcar_1792	transposase of IS*Pca*5, Y1_Tnp domain-containing;<br>phosphomannomutase
Pcar_R8043<br>Pcar_R8044<br>Pcar_R8045	5' of Pcar_1807<br>5' of Pcar_3325	UDP-glucose/UDP-*N*-acetylglucosamine 4-epimerase;<br>hypothetical protein
Pcar_R8046	5' of Pcar_2069;<br>5' of Pcar_2070	protein disulfide bond isomerase DsbC/DsbG;<br>protein of unknown function DUF204

Pcar_R8047	3' of Pcar_2070<br>3' of Pcar_2071	protein of unknown function DUF204;<br>protein of unknown function DUF477
Pcar_R8048<br>Pcar_R8049	5' of Pcar_2071<br>3' of Pcar_2072	protein of unknown function DUF477;<br>23S rRNA (2-*N*-methyl-G2445)-methyltransferase, putative
Pcar_R8050<br>Pcar_R8051	5' of Pcar_2072<br>3' of Pcar_2073	23S rRNA (2-*N*-methyl-G2445)-methyltransferase, putative;<br>sodium/dicarboxylate symporter
Pcar_R8052	3' of Pcar_2422<br>5' of Pcar_2423	dihydrodipicolinate reductase;<br>L,L-diaminopimelate aminotransferase
Pcar_R8053<br>Pcar_R8054	3' of Pcar_2612<br>3' of Pcar_2613	flavodoxin, putative;<br>3'-to-5' exonuclease, putative
Pcar_R8055<br>Pcar_R8056	5' of Pcar_2844<br>3' of Pcar_2845	conserved hypothetical protein;<br>zinc metallopeptidase, SprT family
Pcar_R8057<br>Pcar_R8058	5' of Pcar_3131<br>3' of Pcar_3132	ATP synthase F1, beta subunit;<br>ATP synthase F1, gamma subunit
