# Supplementary material for: The genome of Pelobacter carbinolicus reveals surprising metabolic capabilities and physiological features
Source: BMC Genomics. 2012 Dec 10;13:690. doi: 10.1186/1471-2164-13-690 (PMC3543383; doi:10.1186/1471-2164-13-690)
Supplement: Additional file 3 — Table S2. Gene sets of P.carbinolicus for the catabolism of acetoin/2,3-butanediol, glycerol, 1,3-propanediol, 1,2-ethanediol, ethanolamine and choline, for proton/sodium pumping, hydrogen/formate production and electron transport. [file 1471-2164-13-690-S3.pdf]

Additional file 3: Table S2. Gene sets of *P. carbinolicus* for the catabolism of acetoin/2,3-butanediol, glycerol, 1,3-propanediol, 1,2-ethanediol, ethanolamine and choline, for proton/sodium pumping, hydrogen/formate production and electron transfer.

Locus tag	Gene symbol	Annotation
**2,3-butanediol dehydrogenase *budX* and acetoin dehydrogenase gene cluster**		
Pcar_3188		hypothetical protein
Pcar_0329		membrane protein, putative
Pcar_0330	*budX*	*(2R,3R)*-2,3-butanediol/*meso*-2,3-butanediol dehydrogenase, putative
Pcar_0331		hypothetical protein
Pcar_0332		conserved hypothetical protein
Pcar_0333		methylmalonyl-CoA epimerase family protein
Pcar_3424		hypothetical protein
Pcar_0334		protein of unknown function DUF190
Pcar_0335		conserved hypothetical protein
Pcar_3190		hypothetical protein
Pcar_0336	*acoR-1*	sigma-54-dependent transcriptional regulator, AcoR family (sigma54 interaction, HTH8)
Pcar_3191		hypothetical protein
Pcar_0337	*thiC-2*	4-amino-5-hydroxymethyl-2-methylpyrimidine synthetase
Pcar_0338	*thiS-1*	thiamin biosynthesis sulfur carrier protein
Pcar_0339	*thiG-1*	carboxythiazole phosphate tautomer synthase
Pcar_0340	*thiH-1*	tyrosine lyase
Pcar_0341	*acoX*	acetoin catabolism protein AcoX
Pcar_0342	*tenI-1*	carboxythiazole phosphate tautomerase
Pcar_3192		hypothetical protein
Pcar_0343	*acoA*	acetoin dehydrogenase complex, E1 protein, alpha subunit
Pcar_0344	*acoB*	acetoin dehydrogenase complex, E1 protein, beta subunit
Pcar_0345	*acoC*	acetoin dehydrogenase complex, E2 protein, dihydrolipoamide acetyltransferase
Pcar_0346	*acoS*	lipoate synthase
Pcar_0347	*acoL*	dihydrolipoamide dehydrogenase
Pcar_0348		ferredoxin and NADH nitroreductase domain protein
Pcar_0349		3-hydroxyacyl-CoA dehydrogenase
Pcar_0350	*lipB*	octanoyl-(acyl carrier protein)--protein octanoyltransferase
Pcar_0351		hydrolase or acyltransferase, alpha/beta fold family
**2,3-butanediol dehydrogenase *budY* gene cluster**		
Pcar_3259		hypothetical protein
Pcar_3258		hypothetical protein
Pcar_0903	*budY*	*(2S,3S)*-2,3-butanediol dehydrogenase, putative
Pcar_0902	*acoR-2*	sigma-54-dependent transcriptional regulator, AcoR family (sigma54 interaction, HTH8)
**2,3-butanediol dehydrogenase *budZ* gene cluster**		

Pcar_2068	*budZ*	*meso*-2,3-butanediol/*(2S,3S)*-2,3-butanediol dehydrogenase, putative
Pcar_2067		oxidoreductase, short-chain dehydrogenase/reductase family
Pcar_2066		histone deacetylase family protein
**acetoin regulator *acoR-3* gene cluster**		
Pcar_1734		sigma-54-dependent transcriptional regulator, AcoR family (sigma54 interaction, HTH8)
Pcar_1733		oxidoreductase, aldo/keto reductase family
**glycerol dehydratase gene cluster**		
Pcar_1400		helix-turn-helix transcriptional response regulator, LuxR family (REC, HTH_LuxR)
Pcar_1399		sensor histidine kinase (Hist_Kin_Sens, PAS, HisKA, HATPase_c)
Pcar_1398		membrane protein, putative
Pcar_1397		glycerol dehydratase
Pcar_1396		glycerol dehydratase-activating enzyme, putative
Pcar_1395		outer membrane channel, putative
Pcar_1394		radical SAM domain iron-sulfur cluster-binding oxidoreductase, DUF4008-related domain-containing
**1,3-propanediol dehydrogenase and SfrB-linked hydrogenase gene cluster**		
Pcar_2515		radical SAM domain iron-sulfur cluster-binding oxidoreductase, DUF4008-related domain-containing
Pcar_R0088		molybdenum cofactor-responsive riboswitch
Pcar_2514		aldehyde:ferredoxin oxidoreductase, tungsten-containing
Pcar_3369	*thiS-3*	thiamin biosynthesis sulfur carrier protein
Pcar_3368		hypothetical protein
Pcar_3367		hypothetical protein
Pcar_2513	*thiF-1*	thiamin biosynthesis thiocarboxylate synthase
Pcar_3477		hypothetical protein
Pcar_2512		outer membrane channel, putative
Pcar_2511		hypothetical protein
Pcar_2510		1,3-propanediol dehydrogenase
Pcar_3366		hypothetical protein
Pcar_2509		membrane protein, putative
Pcar_2508		protein of unknown function DUF190
Pcar_2507		carboxymuconolactone decarboxylase family protein
Pcar_2506		iron-containing alcohol dehydrogenase
Pcar_2505	*iolA*	3-oxopropanoate/2-methyl-3-oxopropanoate dehydrogenase, acylating
Pcar_2504		hypothetical protein, fragment fusion
Pcar_2503	*sfrB*	NADPH oxidoreductase, beta subunit
Pcar_2502	*hndD-1*	cytoplasmic NADPH oxidoreductase-associated [FeFe]-hydrogenase
**radical SAM domain oxidoreductase gene cluster**		

Pcar_2883		SEC-C motif domain protein
Pcar_2884		radical SAM domain iron-sulfur cluster-binding oxidoreductase, DUF4008-related domain-containing
**1,2-ethanediol dehydratase gene cluster**		
Pcar_3440		hypothetical protein
Pcar_0937		1,2-ethanediol dehydratase, putative
Pcar_0938		conserved hypothetical protein
Pcar_0939		kinase, putative
Pcar_0940		helix-turn-helix transcriptional regulator, DeoR family
Pcar_3263		hypothetical protein
Pcar_0941		membrane protein, putative
Pcar_0942		winged-helix DNA-binding domain protein
Pcar_0943		1,2-ethanediol dehydratase-activating enzyme, putative
**ethanolamine ammonia-lyase gene cluster**		
Pcar_0493		conserved hypothetical protein
Pcar_0492		ABC transporter, periplasmic substrate-binding protein
Pcar_0491	*eutBC-1*	ethanolamine ammonia-lyase
Pcar_0490		conserved hypothetical protein
Pcar_R0069		cobalamin-responsive riboswitch
Pcar_0489	*cysG-2*	precorrin-2 dehydrogenase, sirohydrochlorin ferrochelatase, and uroporphyrinogen III *C2*,*C7*-methyltransferase, putative
Pcar_0488	*cobC-3*	adenosylcobalamin-5'-phosphate phosphatase, putative
Pcar_0487	*cobU*	adenosylcobinamide kinase and adenosylcobinamide phosphate guanylyltransferase
Pcar_0486	*cobT*	nicotinate-nucleotide--dimethylbenzimidazole phosphoribosyltransferase
Pcar_0485	*cobS*	cobalamin-5'-phosphate synthase
Pcar_0484	*cobD*, *cbiH-1, cbiP*	L-threonine-*0*-3-phosphate decarboxylase, cobalt-precorrin-3 *C17*-methyltransferase and adenosylcobyric acid synthase
Pcar_0483	*cbiB*	adenosylcobinamide-phosphate synthase
Pcar_0482	*cobA-1*	cob(I)yrinate *a*,*c*-diamide adenosyltransferase
Pcar_0481	*cbiA*	cob(II)yrinate a,c-diamide synthase
Pcar_0480	*cbiC*	cobalt-precorrin-8X methylmutase
Pcar_3203		hypothetical protein
Pcar_0479		radical SAM domain iron-sulfur cluster-binding oxidoreductase, DUF4008-containing
Pcar_0478	*cbiK*	sirohydrochlorin cobaltochelatase, putative
Pcar_0477		conserved hypothetical protein
Pcar_0476	*cbiL*	cobalt-sirohydrochlorin *C20*-methyltransferase
Pcar_0475	*cbiD*	cobalt-precorrin-5B *C1*-methyltransferase
Pcar_0474	*cbiE*	cobalt-precorrin-6B *C5*-methyltransferase, putative
Pcar_0473	*cbiF*	cobalt-precorrin-4 *C11*-methyltransferase
Pcar_0472	*cbiG*	cobalt-precorrin-5A hydrolase

Pcar_0471	*cbiH-2*	cobalt-precorrin-3 *C17*-methyltransferase
Pcar_0470	*cbiJ*	precorrin-6A reductase
Pcar_3201		hypothetical protein
Pcar_0469		conserved hypothetical protein
Pcar_0468		ABC transporter, periplasmic substrate-binding protein
Pcar_0467	*eutBC-2*	ethanolamine ammonia-lyase
Pcar_0466		conserved hypothetical protein
Pcar_0465		metal ABC transporter, ATP-binding protein
Pcar_0464		metal ABC transporter, periplasmic substrate-binding protein
Pcar_0463		metal ABC transporter, membrane protein
Pcar_0462		ATPase, AAA_3 family
Pcar_0461		VWFA superfamily protein, DUF58-containing
Pcar_0460		conserved hypothetical protein
Pcar_0459		VWFA superfamily protein
Pcar_0458		conserved hypothetical protein
Pcar_0457		VWFA superfamily protein
Pcar_R0068		molybdenum cofactor-responsive riboswitch
Pcar_0456	*aorA-2*	aldehyde:ferredoxin oxidoreductase, tungsten-containing
Pcar_0455	*moaD-2*	molybdopterin biosynthesis sulfur carrier protein
Pcar_R0067		cobalamin-responsive riboswitch
Pcar_0454		cobalamin uptake ligand-gated TonB-dependent outer membrane channel
Pcar_0453		periplasmic energy transduction protein, TonB-related
**primary ancestral ATP synthase gene set (major operon)**		
Pcar_3136	*atpX-3*	ATP synthase F_0, B' subunit
Pcar_3135	*atpF-3*	ATP synthase F_0, B subunit
Pcar_3134	*atpH-3*	ATP synthase F_1, delta subunit
Pcar_3133	*atpA-3*	ATP synthase F_1, alpha subunit
Pcar_3132	*atpG-3*	ATP synthase F_1, gamma subunit
Pcar_3131	*atpD-3*	ATP synthase F_1, beta subunit
Pcar_3130	*atpC-3*	ATP synthase F_1, epsilon subunit
**primary ancestral ATP synthase gene set (minor operon)**		
Pcar_0013	*atpZ*	ATP synthase-associated magnesium import membrane protein AtpZ
Pcar_0014	*atpI*	ATP synthase-associated magnesium import membrane protein AtpI
Pcar_0015	*atpB-3*	ATP synthase F_0, A subunit
Pcar_0016	*atpE-3*	ATP synthase F_0, C subunit
**duplicate ancestral ATP synthase gene set (ColR regulon)**		
Pcar_0944	*atpX-1*	ATP synthase F_0, B' subunit
Pcar_0945	*atpF-1*	ATP synthase F_0, B subunit
Pcar_0946	*atpH-1*	ATP synthase F_1, delta subunit
Pcar_0947	*atpA-1*	ATP synthase F_1, alpha subunit
Pcar_0948	*atpG-1*	ATP synthase F_1, gamma subunit

Pcar_0949	*atpD-1*	ATP synthase F_1, beta subunit
Pcar_0950	*atpC-1*	ATP synthase F_1, epsilon subunit
Pcar_0951	*atpB-1*	ATP synthase F_0, A subunit
Pcar_0952	*atpE-1*	ATP synthase F_0, C subunit
**N-type ATP synthase gene set**		
Pcar_2988	*atpD-2*	ATP synthase N, beta subunit
Pcar_2989	*atpC-2*	ATP synthase N, epsilon subunit
Pcar_2990	*atpQ*	ATP synthase N, Q subunit
Pcar_2991	*atpR*	ATP synthase N, R subunit
Pcar_2992	*atpB-2*	ATP synthase N, A subunit
Pcar_2993	*atpE-2*	ATP synthase N, C subunit
Pcar_2994	*atpF-2*	ATP synthase N, B subunit
Pcar_2995	*atpA-2*	ATP synthase N, alpha subunit
Pcar_2996	*atpG-2*	ATP synthase N, gamma subunit
Pcar_2997	*atpD-2*	ATP synthase N, beta subunit
**sodium/proton antiporter complex Mrp gene cluster**		
Pcar_2622	*mrpA*	sodium/proton antiporter complex Mrp, protein A
Pcar_2621	*mrpB*	sodium/proton antiporter complex Mrp, protein B
Pcar_2620	*mrpC*	sodium/proton antiporter complex Mrp, protein C
Pcar_2619	*mrpD*	sodium/proton antiporter complex Mrp, protein D
Pcar_2618	*mrpE*	sodium/proton antiporter complex Mrp, protein E
Pcar_2617	*mrpF*	sodium/proton antiporter complex Mrp, protein F
Pcar_2616	*mrpG*	sodium/proton antiporter complex Mrp, protein G
**sodium/proton antiporter NhaA gene**		
Pcar_1390	*nhaA*	pH-dependent sodium/proton antiporter NhaA
**sodium/proton antiporter NhaD gene cluster**		
Pcar_0111	*nhaD*	sodium/proton antiporter NhaD
Pcar_0110	*hemN*	oxygen-independent coproporphyrinogen III oxidase
**sodium/proton-translocating complex Rnf gene cluster**		
Pcar_0265	*rnfA*	ion-translocating NADH:ferredoxin oxidoreductase complex Rnf, membrane protein subunit RnfA
Pcar_0264	*rnfB*	ion-translocating NADH:ferredoxin oxidoreductase complex Rnf, polyferredoxin membrane protein subunit RnfB
Pcar_0263	*rnfC*	ion-translocating NADH:ferredoxin oxidoreductase complex Rnf, SLBB domain and iron-sulfur cluster-binding subunit RnfC
Pcar_0262	*rnfD*	ion-translocating NADH:ferredoxin oxidoreductase complex Rnf, FMN-binding membrane protein subunit RnfD
Pcar_0261	*rnfG*	ion-translocating NADH:ferredoxin oxidoreductase complex Rnf, FMN-binding membrane protein subunit RnfG
Pcar_0260	*rnfE*	ion-translocating NADH:ferredoxin oxidoreductase complex Rnf, membrane protein subunit RnfE

***nfn* genes (two locations)**		
Pcar_0753	*nfnB-1*	NADH-dependent ferredoxin:NADP+ oxidoreductase, beta subunit
Pcar_0752	*nfnA*	NADH-dependent ferredoxin:NADP+ oxidoreductase, alpha subunit
Pcar_0678	*nfnB-2*	NADH-dependent ferredoxin:NADP+ oxidoreductase, beta subunit
**aspartate ammonia-lyase-linked hydrogenase gene cluster**		
Pcar_1636	*hndA-2*	cytoplasmic [FeFe]-hydrogenase-associated NADPH oxidoreductase, ferredoxin-like subunit
Pcar_1635	*hndB-2*	cytoplasmic [FeFe]-hydrogenase-associated NADPH oxidoreductase, thioredoxin-like subunit
Pcar_1634	*hndC-2*	cytoplasmic [FeFe]-hydrogenase-associated NADPH oxidoreductase, major subunit
Pcar_1633	*hndD-2*	cytoplasmic NADPH oxidoreductase-associated [FeFe]-hydrogenase
Pcar_1632	*hydE-2*	[FeFe]-hydrogenase maturation radical SAM domain iron-sulfur cluster-binding oxidoreductase HydE
Pcar_1631	*hydG*	[FeFe]-hydrogenase maturation radical SAM domain iron-sulfur cluster-binding oxidoreductase HydG
Pcar_1630	*aspA*	aspartate ammonia-lyase
Pcar_1629	*hydF*	[FeFe]-hydrogenase maturation GTPase HydF
**oxidoreductase-linked hydrogenase gene cluster**		
Pcar_1602	*hndA-3*	cytoplasmic [FeFe]-hydrogenase-associated NADPH oxidoreductase, ferredoxin-like subunit
Pcar_1603	*hndB-3*	cytoplasmic [FeFe]-hydrogenase-associated NADPH oxidoreductase, thioredoxin-like subunit
Pcar_1604	*hndC-3*	cytoplasmic [FeFe]-hydrogenase-associated NADPH oxidoreductase, major subunit
Pcar_1605	*hndD-3*	cytoplasmic NADPH oxidoreductase-associated [FeFe]-hydrogenase
Pcar_1606		iron-sulfur cluster-binding oxidoreductase, CCG domain pair-containing
Pcar_1607	*hydE-3*	[FeFe]-hydrogenase maturation radical SAM domain iron-sulfur cluster-binding oxidoreductase HydE
**arsenate reductase-linked gene paralog-linked NADPH oxidoreductase gene cluster**		
Pcar_2710		sensor histidine kinase (PAS, PAS, PAS, PAS, HisKA, HATPase_c)
Pcar_2709		response receiver sensor diguanylate cyclase (REC, PAS, GGDEF)
Pcar_2708	*hndC-5*	NADPH oxidoreductase, major subunit
Pcar_2707		conserved hypothetical protein
Pcar_2706		protein of unknown function DUF502
**formate dehydrogenase gene cluster**		
Pcar_1846	*hndA-1*	cytoplasmic formate dehydrogenase-associated NADPH

		oxidoreductase, ferredoxin-like subunit
Pcar_1845	*hndB-1*	cytoplasmic formate dehydrogenase-associated NADPH oxidoreductase, thioredoxin-like subunit
Pcar_1844	*hndC-1*	cytoplasmic formate dehydrogenase-associated NADPH oxidoreductase, major subunit
Pcar_1843	*fdnG*	cytoplasmic formate dehydrogenase, major subunit, selenocysteine-containing
Pcar_1842		carbonic anhydrase, gamma-family
Pcar_1841		cysteine desulfurase
Pcar_1840	*fdhD*	formate dehydrogenase accessory protein FdhD
Pcar_1839	*mobA*	molybdopterin nucleotidyltransferase
Pcar_1838	*fdhC*	bidirectional formate transporter, putative
**hybrid cluster protein-linked NADPH oxidoreductase gene cluster**		
Pcar_0833	*hndA-4*	NADPH oxidoreductase, ferredoxin-like subunit
Pcar_0834	*hndBC-4*	NADPH oxidoreductase, major, thioredoxin-like and ferredoxin-like subunit
Pcar_0835		iron-sulfur cluster-binding oxidoreductase
Pcar_0836		protein of unknown function DUF72
Pcar_0837	*hcp-1*	iron-sulfur-oxygen hybrid cluster protein (prismane)
**carbon monoxide dehydrogenase gene cluster (shared with *Geobacter*)**		
Pcar_0057	*cooS-2*	carbon monoxide dehydrogenase, catalytic subunit
Pcar_0058	*cooC*	carbon monoxide dehydrogenase accessory protein CooC
Pcar_0059	*cooF-2*	carbon monoxide dehydrogenase-associated iron-sulfur cluster-binding oxidoreductase CooF
Pcar_0060		FAD-dependent pyridine nucleotide-disulfide oxidoreductase family protein
**carbon monoxide dehydrogenase gene cluster (shared with *Clostridium*)**		
Pcar_0888	*cooS-1*	carbon monoxide dehydrogenase, catalytic subunit
Pcar_0887	*cooF-1*	carbon monoxide dehydrogenase-associated iron-sulfur cluster-binding oxidoreductase CooF
Pcar_0886		FAD-dependent pyridine nucleotide-disulfide oxidoreductase family protein
**cytoplasmic thioredoxin and thioredoxin reductase genes (three locations)**		
Pcar_3068	*trxA*	thioredoxin
Pcar_2943	*trxB-1*	thioredoxin reductase
Pcar_0114	*trxB-2*	thioredoxin reductase
**cytoplasmic sulfur reductase and periplasmic thioredoxin gene cluster**		
Pcar_0429		FAD-dependent pyridine nucleotide-disulfide oxidoreductase family protein, rhodanese homology, sulfur carrier, and DsrE-related domain-containing
Pcar_0428		aromatic hydrocarbon degradation outer membrane protein
Pcar_0427		periplasmic thioredoxin, putative
Pcar_0426		periplasmic thioredoxin, putative
**menaquinol:ferricytochrome *c* oxidoreductase complex ACIII or Act gene cluster**		
Pcar_2533		amino acid-binding ACT domain regulatory protein

Pcar_2534	*def-2*	polypeptide formylmethionine deformylase
Pcar_2535	*lpdA-4*	dihydrolipoamide dehydrogenase
Pcar_2536	*cdd*	cytidine deaminase
Pcar_2537	*udp*	uridine phosphorylase
Pcar_2538		diguanylate cyclase (7TMR-DISMED2, 7TMR-DISM_7TM, GGDEF)
Pcar_2539		regulatory protein, CxxC_CxxC_SSSS domain-containing, putative
Pcar_2540	*larG*	lipoyl carrier protein LarG
Pcar_2541		periplasmic energy transduction protein, TonB-related
Pcar_2542	*pta-1*	phosphate acetyltransferase
Pcar_2543	*ackA-1*	acetate kinase
Pcar_2544	*frx-2*	ferredoxin
Pcar_3370	*tatA*	twin-arginine translocation pathway protein, TatA/TatE family
Pcar_2546		protein of unknown function, MJ0042_CXXC and DUF3426 domain-containing
Pcar_2547		hypoxanthine/guanine phosphoribosyltransferase, putative
Pcar_2548		protein of unknown function DUF1573
Pcar_2549	*actE*	menaquinol oxidoreductase complex ACIII, membrane protein and cytochrome *c* subunit ActE, 1 heme-binding site
Pcar_2550	*actA*	menaquinol oxidoreductase complex ACIII, cytochrome *c* subunit ActA, 5 heme-binding sites
Pcar_2551	*actB*	menaquinol oxidoreductase complex ACIII, molybdopterin-binding-like and iron-sulfur cluster-binding subunit ActB
Pcar_2552	*actC*	menaquinol oxidoreductase complex ACIII, menaquinol-binding membrane protein subunit ActC
Pcar_2553	*actD*	menaquinol oxidoreductase complex ACIII, DUF3341 subunit ActD
Pcar_2554	*actF*	menaquinol oxidoreductase complex ACIII, menaquinol-binding membrane protein subunit ActF
