# Supplementary material for: The genome of Pelobacter carbinolicus reveals surprising metabolic capabilities and physiological features
Source: BMC Genomics. 2012 Dec 10;13:690. doi: 10.1186/1471-2164-13-690 (PMC3543383; doi:10.1186/1471-2164-13-690)
Supplement: Additional file 4 — Table S3. Thiamin and cobalamin biosynthesis genes of P.carbinolicus. [file 1471-2164-13-690-S4.pdf]

Additional file 4: Table S3. Thiamin and cobalamin biosynthesis genes of *P. carbinolicus*.

Locus tag	Gene symbol	Annotation
**Common enzymes of thiamin/molybdopterin/iron-sulfur cluster biosynthesis**		
Pcar_0050	*nifS-1*	nitrogen fixation iron-sulfur cluster assembly cysteine desulfurase NifS
Pcar_0234		cysteine desulfurase family protein
Pcar_0304		cysteine desulfurase family protein
Pcar_1729		cysteine desulfurase family protein
Pcar_1841		cysteine desulfurase
Pcar_1860	*nifS-2*	iron-sulfur cluster assembly cysteine desulfurase NifS
Pcar_2513	*thiF-1*	thiamin biosynthesis thiocarboxylate synthase
Pcar_0611	*thiF-2*	thiamin biosynthesis thiocarboxylate synthase
Pcar_0338	*thiS-1*	thiamin biosynthesis sulfur carrier protein
Pcar_0610	*thiS-2*	thiamin biosynthesis sulfur carrier protein
Pcar_3369	*thiS-3*	thiamin biosynthesis sulfur carrier protein
**Thiamin biosynthesis**		
Pcar_0340	*thiH-1*	tyrosine lyase
Pcar_0608	*thiH-2*	tyrosine lyase
Pcar_0603		fumarylacetoacetate hydrolase family protein
Pcar_0339	*thiG-1*	carboxythiazole phosphate tautomer synthase
Pcar_0609	*thiG-2*	carboxythiazole phosphate tautomer synthase
Pcar_0342	*tenI-1*	carboxythiazole phosphate tautomerase
Pcar_0607	*tenI-2*	carboxythiazole phosphate tautomerase
Pcar_2235	*thiE, thiD*	4-amino-5-hydroxymethyl-2-methylpyrimidine-phosphate kinase and thiamin monophosphate synthase
Pcar_0028	*thiL*	thiamin monophosphate kinase
Pcar_0035		thiamin biosynthesis protein ApbE
**Common enzymes of cobalamin/siroheme/heme biosynthesis**		
Pcar_3064	*hemA*	glutamyl-tRNA reductase
Pcar_0266	*hemL*	glutamate-1-semialdehyde 2,1-aminomutase
Pcar_3061	*hemB*	porphobilinogen synthase
Pcar_3063	*hemC*	hydroxymethylbilane synthase
Pcar_3062	*hemD*	uroporphyrinogen III *C2*,*C7*-methyltransferase and uroporphyrinogen III synthase
**Cobalamin biosynthesis**		
Pcar_3066	*cysG-1*	precorrin-2 dehydrogenase and sirohydrochlorin ferrochelatase, putative
Pcar_0489	*cysG-2*	precorrin-2 dehydrogenase, sirohydrochlorin ferrochelatase, and uroporphyrinogen III *C2,C7*-methyltransferase, putative
Pcar_2740	*cbiX*	sirohydrochlorin cobaltochelatase
Pcar_0478	*cbiK*	sirohydrochlorin cobaltochelatase, putative
Pcar_0476	*cbiL*	cobalt-sirohydrochlorin *C20*-methyltransferase
Pcar_0484	*cobD*, *cbiH-*	L-threonine-*0*-3-phosphate decarboxylase, cobalt-precorrin-

	1, cbiP	3 *C17*-methyltransferase and adenosylcobyric acid synthase
Pcar_0471	*cbiH-2*	cobalt-precorrin-3 *C17*-methyltransferase
Pcar_0473	*cbiF*	cobalt-precorrin-4 *C11*-methyltransferase
Pcar_0472	*cbiG*	cobalt-precorrin-5A hydrolase
Pcar_0475	*cbiD*	cobalt-precorrin-5B *C1*-methyltransferase
Pcar_0470	*cbiJ*	precorrin-6A reductase
Pcar_2739	*cbiET*	cobalt-precorrin-6B *C5*,*C15*-methyltransferase and *C12*-decarboxylase
Pcar_0474	*cbiE*	cobalt-precorrin-6B *C5*-methyltransferase, putative
Pcar_0480	*cbiC*	cobalt-precorrin-8X methylmutase
Pcar_0481	*cbiA*	cob(II)yrinate a,c-diamide synthase
Pcar_0482	*cobA-1*	cob(I)yrinate *a*,*c*-diamide adenosyltransferase
Pcar_3106	*cobA-2*	cob(I)yrinate *a*,*c*-diamide adenosyltransferase
Pcar_0483	*cbiB*	adenosylcobinamide-phosphate synthase
Pcar_0487	*cobU*	adenosylcobinamide kinase and adenosylcobinamide phosphate guanylyltransferase
Pcar_0486	*cobT*	nicotinate-nucleotide--dimethylbenzimidazole phosphoribosyltransferase
Pcar_0485	*cobS*	cobalamin-5'-phosphate synthase
Pcar_2741	*cobC-1*	adenosylcobalamin-5'-phosphate phosphatase, putative
Pcar_0229	*cobC-2*	adenosylcobalamin-5'-phosphate phosphatase, putative
Pcar_0488	*cobC-3*	adenosylcobalamin-5'-phosphate phosphatase, putative
