# Supplementary material for: The genome of Pelobacter carbinolicus reveals surprising metabolic capabilities and physiological features
Source: BMC Genomics. 2012 Dec 10;13:690. doi: 10.1186/1471-2164-13-690 (PMC3543383; doi:10.1186/1471-2164-13-690)
Supplement: Additional file 5 — Table S4. Cytochrome c proteins and biogenesis factors, TonB-dependent transport systems, tetrapyrrole methyltransferases, and outer surface features of P.carbinolicus: genes for biogenesis of geopilin pili, Msh pili, Pih pili, type II secretion systems, Flp pili, sigma-fimbriae, flagella, autotransporters, and the type VI secretion system. [file 1471-2164-13-690-S5.pdf]

Additional file 5: Table S4. Cytochrome *c* proteins and biogenesis factors, TonB-dependent transport systems, tetrapyrrole methyltransferases, and outer surface features of *P. carbinolicus*: genes for biogenesis of geopilin pili, Msh pili, Pih pili, type II secretion systems, Flp pili, sigma-fimbriae, flagella, autotransporters, and the type VI secretion system.

**Locus tag**	**Gene symbol**	**Annotation**
	**c-type cytochromes**	
Pcar_0152		cytochrome *c*, 1 heme-binding site
Pcar_0181		sensor histidine kinase response regulator (PAS, PAS, HisKA, HATPase_c, REC), putative heme-binding site
Pcar_0192		molybdopterin-binding tetrapyrrole methyltransferase, one heme-binding site, putative
Pcar_0558		lipoprotein cytochrome *c*, 1 heme-binding site
Pcar_1628	*ppcA*	cytochrome *c*, 3 heme-binding sites
Pcar_1961		cytochrome *c*, 1 heme-binding site
Pcar_2069		protein disulfide bond isomerase, DsbC/DsbG-like, one heme-binding site
Pcar_2529	*coxB*	cytochrome *c* oxidase, coo_3-type, cytochrome *c* subunit II, one heme-binding site
Pcar_2549	*actE*	menaquinol oxidoreductase complex ACIII, membrane protein and cytochrome *c* subunit ActE, 1 heme-binding site
Pcar_2550	*actA*	menaquinol oxidoreductase complex ACIII, cytochrome *c* subunit ActA, 5 heme-binding sites
Pcar_2570		cytochrome *c*, 1 heme-binding site
Pcar_2745		cytochrome *c*, 3 heme-binding sites
Pcar_2767		cytochrome *c*, 3 heme-binding sites
Pcar_2866	*nrfA*	cytochrome *c* nitrite and sulfite reductase, catalytic subunit, 5 heme-binding sites
Pcar_2867	*nrfH*	cytochrome *c* nitrite and sulfite reductase, menaquinol-oxidizing subunit, 4 heme-binding sites
Pcar_2984		lipoprotein cytochrome *c*, 2 heme-binding sites
	**cytochrome c biogenesis factors**	
Pcar_1954	*resA*	apocytochrome *c* disulfide reductase lipoprotein ResA
Pcar_1953	*ccdA*	cytochrome *c* biogenesis protein CcdA
Pcar_0193		ResB-like family cytochrome *c* biogenesis protein
Pcar_2229		ResB-like family cytochrome *c* biogenesis protein
Pcar_0194		ResC/HemX-like cytochrome *c* biogenesis membrane protein
Pcar_2228		ResC/HemX-like cytochrome *c* biogenesis membrane protein
Pcar_3065		ResC/HemX-like cytochrome *c* biogenesis membrane protein

**TonB-dependent outer membrane transport system components**		
Pcar_0151		ligand-gated TonB-dependent outer membrane channel
Pcar_0160		ligand-gated TonB-dependent outer membrane channel
Pcar_0195		ligand-gated TonB-dependent outer membrane channel
Pcar_0454	*btuB*	cobalamin uptake ligand-gated TonB-dependent outer membrane channel
Pcar_0852	*cirA*	Fe(III) uptake ligand-gated TonB-dependent outer membrane channel
Pcar_3436		TonB-dependent outer membrane channel, C-terminal fragment
Pcar_2397		ligand-gated TonB-dependent outer membrane channel
Pcar_2970		ligand-gated TonB-dependent outer membrane channel
Pcar_0453		periplasmic energy transduction protein, TonB-related
Pcar_0845		periplasmic energy transduction protein, TonB-related
Pcar_2389		periplasmic energy transduction protein, TonB-related
Pcar_2541		periplasmic energy transduction protein, TonB-related
Pcar_2976		periplasmic energy transduction protein, TonB-related
Pcar_0846		biopolymer transport membrane protein, TolR-related
Pcar_1506		biopolymer transport membrane protein, TolR-related
Pcar_2390		biopolymer transport membrane protein, TolR-related
Pcar_2977		biopolymer transport membrane protein, TolR-related
Pcar_0847		biopolymer transport membrane proton channel, TolQ-related
Pcar_0848		biopolymer transport membrane proton channel, TolQ-related
Pcar_1505		biopolymer transport membrane proton channel, TolQ-related
Pcar_1655		biopolymer transport membrane proton channel, TolQ-related
Pcar_2391		biopolymer transport membrane proton channel, TolQ-related
Pcar_2978		biopolymer transport membrane proton channel, TolQ-related
Pcar_2975		biopolymer transport periplasmic beta-propeller repeat protein, TolB-related
Pcar_2974		peptidoglycan-binding outer membrane lipoprotein Pal, OmpA family
Pcar_2973		TPR domain lipoprotein
**tetrapyrrole methyltransferases**		
Pcar_0191		molybdopterin-binding tetrapyrrole methyltransferase, putative
Pcar_0192		molybdopterin-binding tetrapyrrole methyltransferase, one heme-binding site, putative
Pcar_0851		tetrapyrrole methyltransferase, putative
**geopilin gene cluster**		

Pcar_2161		sensor histidine kinase (HAMP, HisKA, HATPase_c)
Pcar_2160		sigma-54-dependent transcriptional response regulator (REC, sigma54 interaction, HTH8)
Pcar_2159	*fimU*	type IV pilus minor pilin FimU
Pcar_2158	*pilV*	type IV pilus minor pilin PilV
Pcar_2157	*pilW*	type IV pilus minor pilin PilW
Pcar_2156	*pilX*	type IV pilus minor pilin PilX
Pcar_2155	*pilY1*	type IV pilus assembly protein PilY1
Pcar_2154	*pilE*	type IV pilus minor pilin PilE
Pcar_2153		protein of unknown function DUF1015
Pcar_2152	*rnr*	exoribonuclease R
Pcar_2151	*ribF*	riboflavin kinase and FAD synthetase
Pcar_2150	*aroE-1*	shikimate 5-dehydrogenase
Pcar_2149	*pilB*	type IV pilus biogenesis ATPase PilB
Pcar_2148	*pilT-4*	twitching motility pilus retraction ATPase
Pcar_2147	*pilC*	type IV pilus inner membrane protein PilC
Pcar_2146	*pilS*	sensor histidine kinase PilS (PAS, HisKA, HATPase_c)
Pcar_2145	*pilR*	sigma-54-dependent transcriptional response regulator PilR (REC, sigma54 interaction, HTH8)
Pcar_2144	*pilA-1*	geopilin
Pcar_2143	*pilA-2*	geopilin
Pcar_2142		ABC transporter, ATP-binding protein
Pcar_2141		membrane protein, putative
Pcar_2140		conserved hypothetical protein
Pcar_2139	*pilD*	type IV prepilin-like proteins leader peptide processing enzyme
Pcar_2138	*pilM*	type IV pilus biogenesis ATPase PilM
Pcar_2137	*pilN*	type IV pilus biogenesis protein PilN
Pcar_2136	*pilO*	type IV pilus biogenesis protein PilO
Pcar_2135	*pilP*	type IV pilus assembly lipoprotein PilP
Pcar_2136	*pilQ*	type IV pilus secretin PilQ
Pcar_2133	*aroC*	chorismate synthase
Pcar_2132	*aroB*	3-dehydroquinate synthase
Pcar_2131		TPR domain protein
Pcar_2130		GTPase-activating protein, putative
**geopilin domain membrane protein gene**		
Pcar_2773		geopilin domain membrane protein
**Msh pilin gene cluster**		
Pcar_0381	*mshI1*	type IV pilus biogenesis ATPase MshI1
Pcar_0382	*mshI2*	type IV pilus biogenesis protein MshI2
Pcar_0383	*mshJ*	type IV pilus biogenesis protein MshJ, putative
Pcar_0384	*mshK*	type IV pilus biogenesis protein MshK, putative
Pcar_0385	*mshL*	type IV pilus secretin MshL
Pcar_0386	*mshM*	type IV pilus retraction ATPase MshM
Pcar_0387	*mshN*	type IV pilus biogenesis protein MshN, putative

Pcar_0388	*mshE*	type IV pilus biogenesis ATPase MshE
Pcar_0389	*mshG*	type IV pilus inner membrane protein MshG
Pcar_0390	*mshB*	type IV pilus minor pilin MshB, putative
Pcar_0391	*mshA*	type IV pilus major pilin MshA, putative
Pcar_0392	*mshC*	type IV pilus minor pilin MshC, putative
Pcar_0393	*mshD*	type IV pilus minor pilin MshD, putative
Pcar_0394	*mshO*	type IV pilus minor pilin MshO, putative
Pcar_0395	*mshP*	type IV pilus minor pilin MshP, putative
Pcar_0396	*mshQ*	type IV pilus biogenesis protein MshQ, putative
Pcar_0397	*proC*	*Δ*1-pyrroline-5-carboxylate reductase
**Pih pilin gene cluster**		
Pcar_3248	*pihC*	type IV pilus biogenesis protein PihC
Pcar_0855		ABC transporter, ATP-binding protein
Pcar_0856		ABC transporter, membrane protein
Pcar_0857		ABC transporter, periplasmic substrate-binding protein
Pcar_3436		TonB-dependent outer membrane channel, C-terminal fragment
Pcar_0858		conserved hypothetical protein
Pcar_0859		helix-turn-helix SAM-dependent methyltransferase
Pcar_0860		flavodoxin, putative
Pcar_0861		SAM-dependent methyltransferase, putative
Pcar_0862		helix-turn-helix transcriptional activator, AraC family
Pcar_0863	*pihD*	type IV pilus minor pilin PihD
Pcar_0864	*pihO*	type IV pilus biogenesis protein PihO
Pcar_0865	*pihP*	type IV pilus minor pilin PihP
Pcar_0866	*pihQ*	type IV pilus minor pilin PihQ
Pcar_0867	*pihI*	type IV pilus biogenesis ATPase and membrane protein PihI
Pcar_0868	*pihJ*	type IV pilus biogenesis protein PihJ
Pcar_0869	*pihK*	type IV pilus biogenesis protein PihK
Pcar_0870	*pihL*	type IV pilus secretin PihL
Pcar_0871	*pihM*	type IV pilus retraction ATPase PihM
Pcar_0872	*pihE*	type IV pilus biogenesis ATPase PihE
Pcar_0873	*pihG*	type IV pilus inner membrane protein PihG
Pcar_0874	*pihF*	peptidylprolyl *cis-trans* isomerase PihF, PpiC-type
Pcar_0875	*pihB*	type IV pilus biogenesis protein PihB
Pcar_0876	*pihA*	type IV pilus major pilin PihA
Pcar_0877	*pihH*	type IV pilus prepilin peptidase PihH, putative
**type II protein secretion system Pul-1 gene cluster**		
Pcar_1685	*pulF-1*	type II secretion system inner membrane protein PulF
Pcar_1684	*pulE-1*	type II secretion system ATPase PulE
Pcar_1683	*pulM-1*	type II secretion system ATPase PulM, putative
Pcar_1682	*pulN-1*	type II secretion system protein PulN, putative
Pcar_1681	*pulO-1*	type II secretion system protein PulO, putative
Pcar_1680	*pulP-1*	type II secretion system protein PulP, putative

Pcar_1679	*pulQ-1*	type II secretion system secretin lipoprotein PulQ
Pcar_1678	*pulG-1*	type II secretion system pseudopilin PulG
Pcar_3311	*oxpG-1*	type II secretion system pseudopilin OxpG
Pcar_1676	*ftsE*	cell division ATP-binding protein FtsE
Pcar_1675	*ftsX*	cell division ABC transporter, membrane protein FtsX, putative
Pcar_1674		zinc metalloendopeptidase M23 domain protein
Pcar_1673	*ctpA*	periplasmic carboxy-terminal processing protease lipoprotein
Pcar_1672	*yibQ*	protein of unknown function YibQ
**type II protein secretion system pseudopilin TklG-1 gene cluster**		
Pcar_0592	*polA*	DNA polymerase I
Pcar_0593		hypothetical protein
Pcar_0594		lipoprotein, putative
Pcar_0595	*tklG-1*	type II secretion system pseudopilin TklG
Pcar_0596	*mgtE*	magnesium transporter
Pcar_0597	*recO*	DNA repair protein RecO
**type II protein secretion system Pul-2 gene cluster**		
Pcar_0127	*comM-C1*	competence ATPase ComM, C-terminal fragment
Pcar_0128	*tklG-2*	type II secretion system pseudopilin TklG
Pcar_3156		hypothetical protein
Pcar_0129		RHS repeat protein
Pcar_0130	*pulF-2*	type II secretion system inner membrane protein PulF
Pcar_0131	*pulE-2*	type II secretion system ATPase PulE
Pcar_0132	*pulM-2*	type II secretion system ATPase PulM, putative
Pcar_0133	*pulN-2*	type II secretion system protein PulN, putative
Pcar_0134	*pulO-2*	type II secretion system protein PulO, putative
Pcar_0135	*pulP-2*	type II secretion system protein PulP, putative
Pcar_0136	*pulQ-2*	type II secretion system secretin lipoprotein PulQ
Pcar_0137	*pulG-2*	type II secretion system pseudopilin PulG
Pcar_0138	*oxpG-2*	type II secretion system pseudopilin OxpG
Pcar_0139	*comM-C2*	competence ATPase ComM, C-terminal fragment, frameshifted
**type IVb pilus Flp gene cluster**		
Pcar_1743		membrane protein, GtrA superfamily
Pcar_3315	*flp-1*	Flp pilus major pilin
Pcar_3316	*flp-2*	Flp pilus major pilin
Pcar_1745	*tadV*	Flp pilus prepilin peptidase, putative
Pcar_1746	*rcpC*	Flp pilus assembly protein RcpC
Pcar_1747	*rcpA*	Flp pilus secretin RcpA
Pcar_1748		lipoprotein, putative
Pcar_1749	*tadE*	Flp pilus minor pilin TadE
Pcar_1750		conserved hypothetical protein
Pcar_1751	*tadZ*	Flp pilus polar localization response receiver ATPase TadZ (REC, FlhG)

Pcar_1752	*tadA*	Flp pilus assembly ATPase TadA
Pcar_1753	*tadB*	Flp pilus inner membrane protein TadB, putative
Pcar_1754	*tadC*	Flp pilus inner membrane protein TadC, putative
Pcar_1755	*tadD*	Flp pilus assembly TPR domain protein TadD, putative
Pcar_1756		conserved hypothetical protein
**sigma-fimbria gene cluster**		
Pcar_R0084		cyclic diguanylate-responsive riboswitch
Pcar_R0083		cyclic diguanylate-responsive riboswitch
Pcar_2060	*csuA*	sigma-fimbria adhesin, putative
Pcar_2059	*csuB*	sigma-fimbria adhesin, putative
Pcar_2058	*csuC*	sigma-fimbria biogenesis chaperone protein
Pcar_2057	*csuD*	sigma-fimbria biogenesis outer membrane usher protein
Pcar_2056	*csuE*	sigma-fimbria pilin
Pcar_2055	*csuF*	sigma-fimbria biogenesis protein, putative
**long flagellin gene**		
Pcar_0811	*fliC-3*	flagellin
**flagellar biogenesis gene cluster 1 (including glycosylation genes)**		
Pcar_1172	*motA*	flagellar basal body stator protein MotA
Pcar_1171	*motB*	flagellar basal body stator protein MotB
Pcar_1170	*fliL*	flagellar basal body-associated protein FliL
Pcar_1169	*fliM*	flagellar motor switch protein FliM
Pcar_1168	*fliN*	flagellar motor switch protein FliN
Pcar_1167	*fliO*	flagellar biogenesis protein FliO
Pcar_1166	*fliP*	flagellar biogenesis protein FliP
Pcar_1165	*fliQ*	flagellar biogenesis protein FliQ
Pcar_1164	*fliR*	flagellar biogenesis protein FliR
Pcar_1163	*flhB*	flagellar biogenesis protein FlhB
Pcar_1162	*flhA*	flagellar biogenesis protein FlhA
Pcar_1161	*flhF*	flagellar biogenesis protein FlhF
Pcar_1160	*flhG*	flagellar biogenesis ATPase FlhG
Pcar_1159	*fliA*	RNA polymerase sigma-28 factor for flagellar operon
Pcar_1158		hypothetical protein
Pcar_1157	*flgF*	flagellar basal body rod protein FlgF
Pcar_1156	*flgG*	flagellar basal body rod protein FlgG
Pcar_1155	*flgA*	flagellar basal body P-ring formation protein FlgA
Pcar_1154	*flgH*	flagellar L-ring lipoprotein FlgH
Pcar_1153	*flgI*	flagellar P-ring protein FlgI
Pcar_1152	*flgJ*	flagellar rod-binding protein FlgJ
Pcar_1151	*flgM*	negative regulator of flagellin synthesis FlgM
Pcar_1150	*flgN-1*	flagellar biogenesis chaperone FlgN
Pcar_1149	*flgK-1*	flagellar hook-associated protein FlgK
Pcar_1148	*flgL*	flagellar hook-filament junction protein FlgL
Pcar_1147	*csrA*	RNA-binding protein CsrA
Pcar_1146	*fliW*	flagellin-stabilizing protein FliW
Pcar_1145		protein of unknown function DUF115

Pcar_1144		conserved hypothetical protein
Pcar_1143		protein of unknown function (DUF115, TPR)
Pcar_1142	*pseB*	UDP-*N*-acetylglucosamine 4,6-dehydratase and UDP-2-acetamido-2,6-dideoxy-alpha-D-xylo-4-hexulose 5-epimerase
Pcar_1141		UDP-2-acetamido-2,6-dideoxy-beta-L-arabino-4-hexulose 4-aminotransferase, putative
Pcar_1140		*N*-acetylneuraminate synthase family protein
Pcar_1139		oxidoreductase, short-chain dehydrogenase/reductase family
Pcar_1138		oxidoreductase, aldo/keto reductase family
Pcar_1137		glycosyltransferase, SpsF domain-containing
Pcar_1136		glycosyltransferase
Pcar_1135		SAM-dependent methyltransferase
Pcar_1134		radical SAM domain iron-sulfur cluster-binding oxidoreductase with cobalamin-binding-like domain
Pcar_1133		hypothetical protein
Pcar_1132	*ddhA*	glucose-1-phosphate cytidylyltransferase
Pcar_1131	*ddhB*	CDP-glucose 4,6-dehydratase
Pcar_1130	*ddhC*	CDP-4-dehydro-6-deoxyglucose dehydratase/reductase
Pcar_1129		dTDP-4-dehydrorhamnose 3,5-epimerase
Pcar_1128		NAD-dependent nucleoside diphosphate-sugar epimerase/dehydratase
Pcar_1127		SAM-dependent methyltransferase, putative
Pcar_1126		radical SAM domain iron-sulfur cluster-binding oxidoreductase
Pcar_1125		radical SAM domain iron-sulfur cluster-binding oxidoreductase with cobalamin-binding-like domain and TPR domain
Pcar_1124		radical SAM domain iron-sulfur cluster-binding oxidoreductase
Pcar_1123		glycosyltransferase, putative
Pcar_1122		glycosyltransferase
Pcar_1121		2-oxoacid decarboxylase/dehydrogenase/transferase, putative
Pcar_1120	*fabG-5*	3-oxoacyl-(acyl carrier protein) reductase
Pcar_1119		conserved hypothetical protein
Pcar_1118		*N*-acetylglycoside deacetylase, LmbE family
Pcar_1117		glycoside formyltransferase
Pcar_1116		conserved hypothetical protein
Pcar_1115	*fliC-1*	flagellin
Pcar_1114	*fliC-2*	flagellin
Pcar_1113	*flaG*	flagellar protein FlaG
Pcar_1112	*fliD*	flagellar filament cap protein FliD
Pcar_1111	*fliS*	flagellin export facilitator protein FliS

**flagellar biogenesis gene cluster 2 (including chemotaxis genes)**		
Pcar_1208	*fgrM*	flagellar biogenesis master sigma-54-dependent transcriptional response regulator (REC, sigma54 interaction, HTH8)
Pcar_1207		response regulator (REC)
Pcar_1206	*cheX36H*	protein phosphoaspartate phosphatase CheX associated with MCPs of class 36H
Pcar_1205	*cheY36H-2*	response receiver CheY associated with MCPs of class 36H
Pcar_1204		conserved hypothetical protein
Pcar_1203	*cheY36H-1*	response receiver CheY associated with MCPs of class 36H
Pcar_1202		metal-dependent phosphohydrolase (HDOD)
Pcar_1201	*cheD36H*	protein glutamine deamidase and protein glutamate methylesterase CheD associated with MCPs of class 36H
Pcar_1200	*cheB36H*	protein glutamate methylesterase CheB associated with MCPs of class 36H, response receiver domain-containing
Pcar_1199	*cheR36H*	protein glutamate methyltransferase CheR associated with MCPs of class 36H
Pcar_1198	*cheW36H-1*	scaffold protein CheW associated with MCPs of class 36H
Pcar_1197	*cheA36H*	sensor histidine kinase CheA associated with MCPs of class 36H
Pcar_1196		conserved hypothetical protein
Pcar_1195	*flgB*	flagellar basal body rod protein FlgB
Pcar_1194	*flgC*	flagellar basal body rod protein FlgC
Pcar_1193	*fliE*	flagellar hook-basal body complex protein FliE
Pcar_1192	*fliF*	flagellar M-ring mounting plate protein FliF
Pcar_1191	*fliG*	flagellar motor switch protein FliG
Pcar_1190	*fliH*	flagellar assembly protein FliH
Pcar_1189	*fliI*	flagellum-specific ATPase FliI
Pcar_1188	*fliJ*	flagellar export protein FliJ
Pcar_1187		conserved hypothetical protein
Pcar_1186	*fliK*	flagellar hook-length control protein FliK
Pcar_1185	*flgD*	flagellar hook capping protein FlgD
Pcar_1184		flagellar operon protein of unknown function DUF3766
Pcar_1183	*flgE*	flagellar hook protein FlgE
**flagellar hook-associated protein and chaperone genes**		
Pcar_2444	*flgN-2*	flagellar biogenesis chaperone FlgN
Pcar_2445	*flgK-2*	flagellar hook-associated protein FlgK
**autotransporters**		
Pcar_0046		autotransporter domain outer membrane protein and cysteine peptidase
Pcar_0519		autotransporter domain outer membrane protein, putative
Pcar_1176		autotransporter domain outer membrane protein
Pcar_2803		autotransporter domain outer membrane protein, putative
**type VI secretion system gene cluster**		
Pcar_2807		hypothetical protein

Pcar_2808	*tssJ*	type VI secretion system outer membrane lipoprotein TssJ
Pcar_2809	*tssK*	type VI secretion system protein TssK
Pcar_2810	*tssL*	type VI secretion system inner membrane protein TssL
Pcar_2811	*tssM*	type VI secretion system ATPase and inner membrane protein TssM
Pcar_2812		type VI secretion system protein of unknown function DUF2094
Pcar_2813		type VI secretion system ImpA-related domain protein
Pcar_2814	*tssB*	type VI secretion system needle sheath protein TssB
Pcar_2815	*tssC*	type VI secretion system needle sheath protein TssC
Pcar_3483		hypothetical protein
Pcar_2816	*tssD*	type VI secretion system needle tube protein TssD
Pcar_2817	*tssE*	type VI secretion system needle hub protein TssE
Pcar_2818	*tssF*	type VI secretion system protein TssF
Pcar_2819	*tssG*	type VI secretion system protein TssG
Pcar_2820	*tssH*	type VI secretion system ATPase TssH, putative chaperone
Pcar_3484		hypothetical protein, fragment
Pcar_2821	*tssI-1*	type VI secretion system needle syringe protein TssI
Pcar_2822	*tssI-2*	type VI secretion system needle syringe protein TssI
Pcar_2823		hypothetical protein
Pcar_2824		pentapeptide repeat domain protein
Pcar_2825		conserved hypothetical protein
Pcar_2826		hypothetical protein
Pcar_2827		protein of unknown function DUF2169
Pcar_2828		conserved hypothetical protein
Pcar_2829		protein of unknown function DUF4150
Pcar_2830		conserved hypothetical protein
Pcar_2831		hypothetical protein
Pcar_3485		hypothetical protein, N-terminal fragment, frameshifted
Pcar_2832		hypothetical protein
