# Supplementary material for: The genome of Pelobacter carbinolicus reveals surprising metabolic capabilities and physiological features
Source: BMC Genomics. 2012 Dec 10;13:690. doi: 10.1186/1471-2164-13-690 (PMC3543383; doi:10.1186/1471-2164-13-690)
Supplement: Additional file 2 — Figure S1. Alignments of multicopy nucleotide sequences of the P.carbinolicus genome. For the locus tags and functional annotations of adjacent genes, see Additional file 1: Table S1. [file 1471-2164-13-690-S2.pdf]

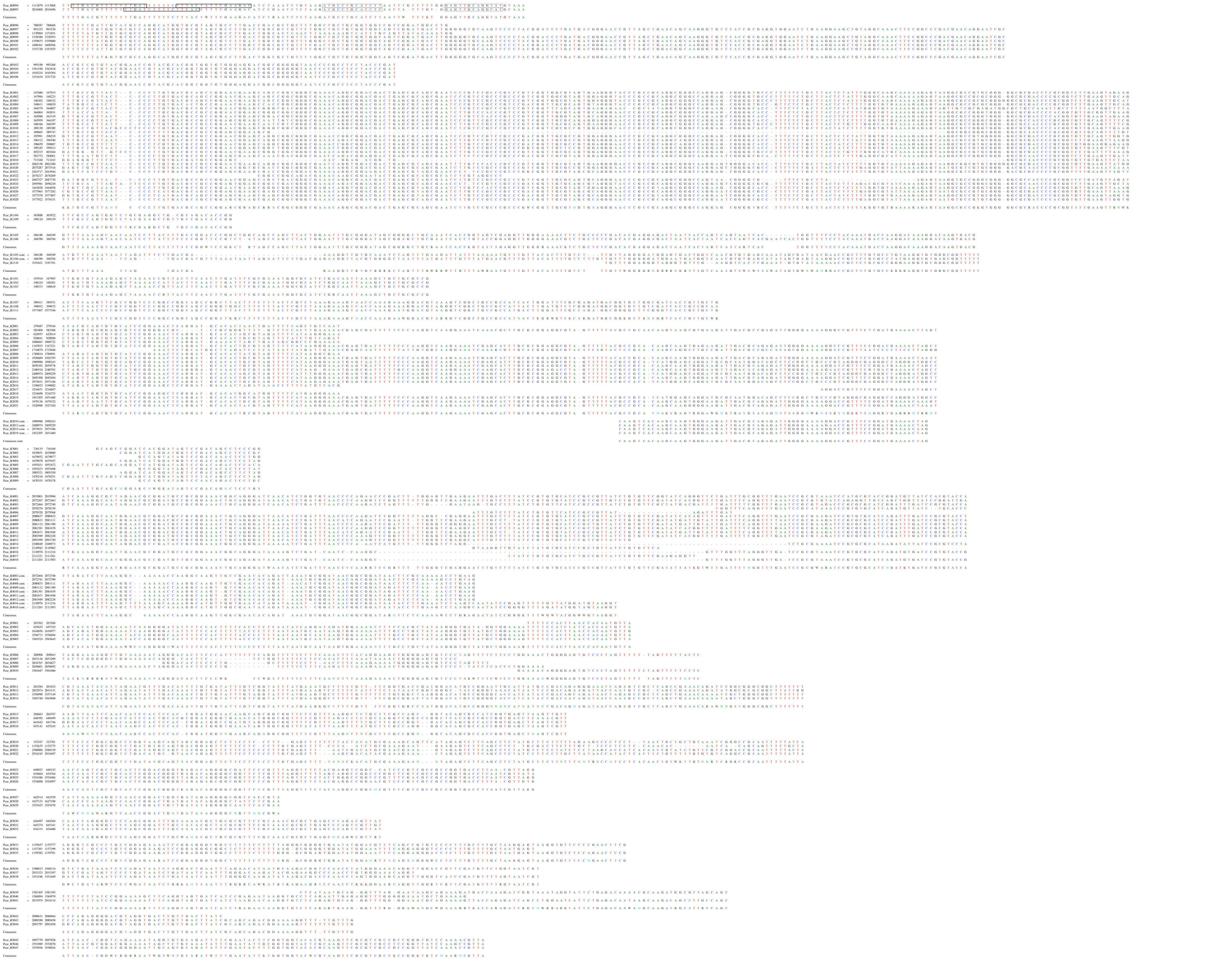

```
Pcar_R5048  -  354642  354682   A A A A C A C A A A A T C C C C G C C C G G A G G C G G G G A T T T T A C T A T C
Pcar_R5049  +  3545191 3545231  A A A A C A C A A A G T C C C C G C C C G G A G G C G A G G A C T T T A T T A T C

Consensus                       A A A A C A C A A A R T C C C C G C C C G G A G G C G R G G A Y T T T A Y T A T C

Pcar_R5050  -  648005  648058   C C A G G C T C T T G C A G C G G A T C G C A A A A G G C C G C G C C C G C T G A A G A G C G A C G T T A G
Pcar_R5051  -  3552718 3552771  C C A G G C T C T T G C A G C G G A T C G C A A A A G G C C G C G C C C G C T G A A G A G C A A C G T T A G

Consensus                       C C A G G C T C T T G C A G C G G A T C G C A A A A G G C C G C G C C C G C T G A A G A G C R A C G T T A G

Pcar_R5052  +  933704  933746   C G G G G G T G A C G C G A A G T G C C C G A T C T T T C G C T A G A T A G A G G T C
Pcar_R5053  -  1837679 1837721  C G G G G G T G A C G C G C A G A T C C C G G A C T T T T G C T A G A T G G A G G T C

Consensus                       C G G G G G T G A C G C G M A G W K C C C G R W C T T T Y G C T A G A T R G A G G T C

Pcar_R5054  -  954851  954989   C T T C A A A C G C C T G A T A T T A G G G C G T T T T T T C G T T T C T A G T T T T C C T T A T T T T C C A T C A C C T T T C G T A A C [illegible]
Pcar_R5055  -  958200  958338   C T T C A A A C G T C C G A T A T T A G G G C G T T T T T T T G T T T C T G A T T C C C C T T A T T T T T C A T C A A C T T T C G C A A A [illegible]

Consensus                       C T T C A A A C G Y C Y G A T A T T A G G G C G T T T T T T Y G T T T C T R R T T Y Y C C T T A T T T T Y C A T C A M C T T T C G Y A A M [illegible]

Pcar_R5056  +  1079697 1079737  T T T A T T A C T T G A C T A T T C A A T T G A A C A A T T G T A T C A A T A T G
Pcar_R5057  +  2934179 2934219  T T T A A T G C T T G A C T A T T C A A T T G A A T A C T A G T A T A G A T A T G

Consensus                       T T T A W T R C T T G A C T A T T C A A T T G A A Y A M T W G T A T M R A T A T G

Pcar_R5058  +  1141705 1141786  C G C G A T T G T T A C T C A A T T T A C G G C C G T A T T T T T A G A C C T G T A A A G C A G T C C A C C T C T T G C C C G G A G G T G [illegible]
Pcar_R5059  +  1141932 1142010  C G C G A T G G T T C G T C A - T T G A T G G C C T T A T T - T A A G G C C T G T A A A G C A G T C C A C C T - T T G C C C G G G G T G G [illegible]

Consensus                       C G C G A T K G T T M S T C A - T T K A Y G G C C K T A T T - T W A G R C C T G T A A A G C A G T C C A C C T - T T G C C C G G R G K K [illegible]

Pcar_R5060  +  1573657 1573751  C T G A A A C T T A G G A T T A C A G T A T G C A C A - A A T A A G A C G G C T C C C C C C T G T T T C A G C C A G T G G C A A G T C G T [illegible]
Pcar_R5061  +  1584789 1584877  C T G A A A C T C A G G A C T G C A G C A T G C A A A A A A T A A G A C G G C T C T T C C - - G G G T C G G C - - - - G G T A A G T C G T [illegible]

Consensus                       C T G A A A C T Y A G G A Y T R C A G Y A T G C A M A A A A T A A G A C G G C T C Y Y C C - - G K K T C R G C - - - - G G Y A A G T C G T [illegible]

Pcar_R5062  +  1719547 1719580  G T A G G A T G T G G T G A G C C T G C G A A C C G C A T C G A T C
```

[illegible]
